# Supplementary material for: Emerging roles of the RNA modifications N6-methyladenosine and adenosine-to-inosine in cardiovascular diseases
Source: Mol Ther Nucleic Acids. 2022 Jul 20;29:426–61. doi: 10.1016/j.omtn.2022.07.018 (PMC9366019; doi:10.1016/j.omtn.2022.07.018)
Supplement: Document S1. Tables S4 and S5 [file mmc1.pdf]

OMTN, Volume *29*

Supplemental information

Emerging roles of the RNA modifications N6-methyladenosine and adenosine-to-inosine in cardiovascular diseases

Vilbert Sikorski, Antti Vento, Esko Kankuri, and on behalf of the IHD-EPITRAN Consortium

TABLE S1. Summary of observational study results assessing m^6A and its core regulators in cardiovascular physiology and diseases

Rough methodological classification: ***, interventional study with experimental evidence either *in vivo,* with multiple lines *in vitro*, or both; *, mainly observational study with direct samples from the target tissue or organ, possibly few interventional methods *in vitro*; *o*, associative/phenotypic study with indirect samples (such as blood), no interventional methods. The degree of certainty for summarized potential therapeutic effect is divided here to be either high (experimental evidence *in vivo*), intermediate† (experimental evidence *in vitro*), or light†† (associative/phenotype evidence). To review the Table S1, please see the Supplementary excel file. Abbreviations are listed within the Supplementary excel as well.

TABLE S2. **Summary of observational and interventional discoveries regarding A-to-I editing in cardiovascular physiology and diseases**

Methodological classification: ***, interventional study with experimental evidence either *in vivo,* with multiple lines *in vitro* or both; *, mainly observational study with direct samples from the target tissue or organ, possibly few interventional methods *in vitro*; *o*, associative/phenotypic study with indirect samples (such as blood), no interventional methods. The degree of certainty for summarized potential therapeutic effect is divided here to be either high (experimental evidence *in vivo*), intermediate† (experimental evidence *in vitro*), or light†† (associative/phenotype evidence). Blue arrows indicate either overexpression or knockdown. To review the Table S2, please see the Supplementary excel file. Abbreviations are listed within the Supplementary excel as well.

TABLE S3. Summary of studies assessing the effects of m^6A manipulation in cardiovascular pathologies or related bioprocesses. While upwards arrow indicates upregulation or increase in the given process in general, downwards arrow indicates downregulation or decrease. To review the Table S3, please see the Supplementary excel file. Abbreviations are listed within the Supplementary excel as well.

TABLE S4. Summary of molecular pathways involving m^{6}A and A-to-I modifications in CVDs. Upwards and downwards arrows indicate upregulation (activation) or downregulation (inhibition), respectively. Blunt arrows represent inhibition.

Abbreviations: ***ADAR1***, adenosine deaminase RNA specific; ***ADAR2***, adenosine deaminase RNA specific B1; ***AGO2***, argonaute RISC catalytic component 2; ***AKT***, AKT serine/threonine kinase; ***ALKBH5***, alkB homolog 5, RNA demethylase; ***AngII***, angiotensin II; ***ANK2***, ankyrin 2; ***ATF4***, activating transcription factor 4; ***AUF1***, ARE/poly(U)-binding/degradation factor 1, alias ***HNRNPD***, heterogeneous nuclear ribonucleoprotein D; ***AVC***, aortic valve calcification; ***BCL2***, BCL2 apoptosis regulator; ***CCL2***, C-C motif chemokine ligand 2; ***CDC42***, cell division cycle 42; ***CDR1as***, cdr1 antisense (a long non-coding RNA); ***CHAPIR***, Cardiac-hypertrophy-associated piRNA (piwi-interacting RNA); ***Chast***, cardiac hypertrophy–associated transcript (a long non-coding RNA); ***CHOP***, C/EBP homologous protein; ***circCELF1***, circular RNA CUGBP Elav-like family member 1; ***CM***, cardiomyocyte; ***CMYA5***, cardiomyopathy associated 5; ***CPT-1A***, carnitine palmitoyltransferase 1A; ***CTNND1***, catenin delta 1; ***CTSL***, cathepsin L; ***CTSS***, cathepsin S; ***CUX1p110***, cut like homeobox 1, isoform p110; ***CXCL8***, C-X-C motif chemokine ligand 8; ***DCM***, dilated cardiomyopathy; ***DDX6***, DEAD-box helicase 6; ***DGCR8***, DGCR8 microprocessor complex subunit; ***DHCR24***, 24-dehydrocholesterol reductase; ***DKK2***, dickkopf WNT signaling pathway inhibitor 2; ***DSP***, desmoplakin; ***DTX1***, deltex E3 ubiquitin ligase 1; ***DTX3L***, deltex E3 ubiquitin ligase 3L; ***EGFR***, epidermal growth factor receptor; ***EIF3A***, eukaryotic translation initiation factor 3 subunit A; ***eNOS***, endothelial nitric oxide synthase; ***ER***, endoplasmic reticulum; ***ET***, electron transport; ***EV***, extracellular vesicle; ***FAK***, focal adhesion kinase; ***FBXO32***, F-box protein 32; ***FOXO1/-3***, forkhead box O1/-3; ***FTO***, FTO alpha-ketoglutarate dependent dioxygenase; ***GLUT4***, insulin-responsive glucose transporter type 4; ***GPX4***, glutathione peroxidase 4; ***GSK3β***, glycogen synthase kinase 3 β; ***HFpEF***, heart failure with preserved ejection fraction; ***HFrEF***, heart failure with reduced ejection fraction; ***H3K4me3***, tri-methylation at the 4th lysine residue of the histone H3 protein; ***HNRNPA2B1***, heterogeneous nuclear ribonucleoprotein A2/B1; ***H/R***, hypoxia-reoxygenation (injury); ***HuR***, human antigen R; ***ICAM-1***, intercellular adhesion molecule 1; ***IGF2BP1/-2/-3***, insulin-like growth factor 2 mRNA binding protein 1/2/3; ***INF-γ***, interferon γ; ***IL-6***, interleukin 6; ***I/R***, ischemia-reperfusion (injury); ***JAK2***, Janus kinase 2; ***KDM5A***, lysine demethylase 5A; ***KIAA1429***, alias ***VIRMA***, vir-like m^{6}A methyltransferase associated; ***KLF4/-5***, krüppel-like factor 4/-5; ***LATS1/-2***, Large tumor suppressor kinase 1/-2; ***L-PGDS***, prostaglandin D synthase; ***LPS***, lipopolysaccharide; ***MAGED1***, MAGE family member D1; ***MAVS***, mitochondrial antiviral-signaling protein; ***MDA5***, melanoma differentiation-associated protein 5; ***METTL3***, methyltransferase 3, N^6-adenosine-methyltransferase complex catalytic subunit; ***METTL14***, methyltransferase 14, N^6-adenosine-methyltransferase subunit; ***METTL16***, methyltransferase 16, N^6-methyladenosine ***Mhrt***, myosin heavy chain associated RNA transcript (a long non-coding RNA); ***MIAT***, myocardial infarction associated transcript (a long non-coding RNA); ***MST1***, macrophage stimulating 1; ***MYH7/-9***, myosin heavy chain 7/-9; ***NACAD***, NAC alpha domain containing; ***NEAT1***, nuclear paraspeckle assembly transcript 1 (a long non-coding RNA); ***NFATC4***, nuclear factor of activated T cells 4; ***NF-κB***, nuclear factor kappa B; ***NLRP1/-3***, NLR family pyrin domain containing 1/-3; ***NPPA***, natriuretic peptide A; ***PARP***, Poly-(ADP-ribose) polymerase; ***PM***, pulmonary hypertension; ***PMD***, pulmonary microvascular dysfunction; ***RYR2***, ryanodine receptor 2; ***SERCA2A***, sarcoplasmic/endoplasmic reticulum Ca^{2+}ATPase 2a; ***SLC7A5***, solute carrier family 7 member 5; ***SLC16A3***, solute carrier family 16 member 3; ***STAT3***, signal transducer and activator of transcription 3; ***PARP10***, poly(ADP-ribose) polymerase family member 10; ***PCNA***, proliferating cell nuclear antigen; ***PFKFB2***, 6-phosphofructo-2-kinase/fructose-2,6-biphosphatase 2; ***PGAM2***, phosphoglycerate mutase 2; ***PGD_2***, prostaglandin D2; ***PIWIL4***, piwi-like RNA-mediated gene silencing 4; ***PM2.5***, fine particulate matter, diameter <2.5 µm; ***PRKCE,*** protein kinase C epsilon type; ***p300***, E1A binding protein p300; ***SIRT1***, sirtuin 1; ***SPHK1***, sphingosine kinase 1; ***SULF2***, sulfatase 2; ***TanIIA***, Tanshinone IIA, ***TCA***, tricarboxylic acid (cycle); ***TGF-β1***, transforming growth factor β1; ***TFEB***, transcription factor EB; ***TINCR***, terminal differentiation-induced non-coding RNA (a long non-coding RNA); ***TNF-α,*** Tumor necrosis factor α; ***TSP1***, thrombospondin-1; ***TWIST1***, twist family bHLH transcription factor 1; ***VCAM-1***, vascular cell adhesion molecule 1; ***WNT1***, wnt family member 1; ***WNT5A***, Wnt family member 5A; ***WTAP***, WT1 associated protein; ***YAP1***, Yes1 associated transcriptional regulator; ***YTHDC1***, YTH domain containing 1; ***YTHDF1/-2***, YTH N^6-methyladenosine RNA binding protein 1/-2.

Table S4 Select summary of identified molecular pathways regarding m^{6}A and A-to-I RNA modifications and their key regulators in CVDs

Class	Process		Pathway / molecular associations: Upstream m^{6}A or A-to-I regulators		Inter-stream (e.g. target RNA and reader complex)		Downstream RNAs / proteins	Reference
CARDIAC	**Cardiogenesis**	↑	ADAR1p150			⊣	MDA5 → MVAS → INF → ER stress	[130,172-174]
	**Regeneration, CM proliferation**	↑	ALKBH5 → *YTHDF1* → YTHDF1	→	(m^{6}A-*YAP1* / YTHDF1)	→	YAP1	Han et al.[54]
			FTO	⊣	(m^{6}A-*Cdc42* / miR-133a / IGF2BP2 / AGO2)	⊣	CDC42	Qian et al.[124]
			METTL3	→	(pri-miR-17-3p / DGCR8)	→	miR-17-3p	Zhao et al.[147]
			ADAR2	→	edited-pri-miR-43a ⊣ miR-43a	⊣	SIRT1, Cyclin D1, BCL2	Wu et al.[146]
		↓	METTL3	→	(m^{6}A-pri-miR-143 / DGCR8) → miR-143-3p ⊣ *Yap1* / *Ctnnd1*	→	YAP1 / CTNND1	Gong et al.[140]
		↔	METTL3	→	*Dhcr24, Nacad, Slc16a3, Slc7a5*			Yang et al.[55]
				⊣	*Ank2, Cmya5, Fbxo32, Pfkfb2*			
	**Cardiogenesis**	↔	ADAR2	→	let-7 miRNA family / miR-29b			Altaf et al.[132]
	**Hypertrophy**	↑	(CHAPIR / PIWIL4) ⊣ METTL3	→	(m^{6}A-*Parp10* / YTHDF2)	→	PARP10 ⊣ GSK3β ⊣ NFATC4	Gao et al.[123]
			MIAT → *Ythdf2* → YTHDF2	→	(m^{6}A-*Cpt-1a* / YTHDF2)	⊣	CPT-1A	Yang et al.[129]
			USP12 → p300	→	*Mettl3*	→	METTL3	Lu et al.[126]
			Leptin	→	p-JAK2 → p-STAT3 → CTSL → CUX1 p110	→	FTO	Gan et al.[118]
		↓	FTO	→	(miR-133a / IGF2BP2 / AGO2)			Qian et al.[124]
			Maslinic acid	⊣	*Mettl3*	→	METTL3	Fang et al.[128]
			Tanshinone IIA ⊣ *Alkbh5* → ALKBH5	⊣	m^{6}A-*Galectin-3*	⊣	Galectin-3	Zhang et al.[141]
	**Myocardial infarction**	↑	KDM5A → H3K4me3 → METTL3	→	(m^{6}A-pri-miR-503/ HNRNPA2B1) → EV-packed miR-503	⊣	PGC-1β , SIRT1 → ET chain, TCA cycle	Sun et al.[148]
		↓	FTO	⊣	m^{6}A-(*Nppa, Myh7, Serca2a, Myh9, Ryr2, Ttn, Chast, Mhrt*)	→	SERCA2A	Mathiyalagan et al.[107]
	**I/R, H/R injury**	↑	WTAP	→	m^{6}A-*ATF4*	→	ATF4 → WTAP	Wang et al.[136]
			METTL3	→	(m^{6}A-*Tfeb* / HNRNPD)	→	TFEB → *Alkbh5* → ALKBH5; ⊣ *Mettl3* → METTL3	Song et al.[134]
			METTL3	→	(m^{6}A-pri-miR-143-3p / DGCR8) → miR-143-3p	⊣	PRKCE	Wang et al.[142]
		↓	METTL14	→	m^{6}A-*Wnt1*	→	WNT1 → β-catenin	Pang et al.[143]
			FTO	⊣	m^{6}A-*Mhrt* (lncRNA)	⊣	*Mhrt* (lncRNA)	Shen et al.[135]
			FTO	⊣	m^{6}A-*Yap1*	⊣	YAP1	Ke et al.[145]
	**Cardiac fibrosis**	↑	AngII → circCELF1	→	FTO ⊣ (m^{6}A-*Dkk2* / miR-363)	⊣	DKK2	Li et al.[149]
	**HFpEF**	(↓)	FTO	⊣	m^{6}A-*Pgam2*	⊣	PGAM2	Zhang et al.[175]
				→	p-AKT → *Glut4*	→	GLUT4	
	**HFrEF**	↔	FTO	⊣	m^{6}A-*Mhrt* (lncRNA)	⊣	*Mhrt* (lncRNA)	Shen et al.[135]
	**DCM**	↓			(m^{6}A-*Titin* / YTHDC1) → (alternative splicing) → *N2B-Titin*	→	N2B-Titin	Gao et al.[150]
	**Diabetic cardiomyopathy**	↑	ALKBH5	⊣	(m^{6}A-*Foxo3* / YTHDF2) ⊣ FOXO3	⊣	*CDR1as* (lncRNA) → MST1 → LATS1/2 ⊣ YAP1	Shao et al.[153]
		↓	METTL14	→	(m^{6}A-*TINCR* / YTHDF2) ⊣ *TINCR* (lncRNA)	→	*Nlrp3* → NLRP3	Meng et al.[152]
	**AVC**	↑	METTL3	→	(m^{6}A-*TWIST1* / YTHDF2)	⊣	TWIST1	Zhou et al.[157]
	**CM inflammation**	↑	Palmitic acid	→	CD36 [⊢ (*Cd36*-m^{6}A) ⊢ FTO]	→	TNF-α, IL-6, p-P65	Yu et al.[154]

Cont'd

Cont'd Table S4

Class	Process		Pathway / molecular associations: Upstream m^{6}A or A-to-I regulators		Inter-stream (e.g. target RNA and reader complex)		Downstream RNAs / proteins	Reference
**VASCULAR**	**Atherosclerosis, endotheliopathy**	↑	TNFα → METTL14	→	(m^{6}A-*FOXO1* / YTHDF1)	→	FOXO1 → VCAM-1, ICAM-1	Jian et al.[90]
			Oscillatory shear → METTL3*	→	(m^{6}A-*NLRP* / YTHDF1)	→	NLRP, (+ NF-κB)	Chien et al.[92]
				→	(m^{6}A-*KLF4* / YTHDF2)	⊣	KLF4	
			oxLDL → METTL3	→	(m^{6}A-*JAK2* / IGF2BP1)	→	JAK2 → p-STAT3	Dong et al.[97]
			METTL14	→	(m^{6}A-*pri-miR-19a* +DGCR8)	→	miR-19a	Zhang et al.[91]
			TNF-α → ADAR1	→	(edited-*NEAT1* (lncRNA) / AUF1)	→	*CCL2, CXL8, ICAM-1, VCAM-1*	Vlachogiannis et al.[103]
		↓	Oscillatory shear ⊣ METTL3*	→	m^{6}A-*EGFR*	⊣	EGFR	Li et al.[95]
		↔	TNF-α / INF-γ / hypoxia → ADAR1	→	(edited-*CTSS* / HuR)	→	CTSS	Stellos et al.[50]
	**Hypertension**	↑	Obesity → FTO	⊣	*L-Pdgs*	→	L-PDGS → PGD_2 → myogenic tone	Krüger et al.[117]
	**PH**	↑	WTAP	→	m^{6}A-*Gpx4*	→	GPX4 PREPRINT	Wei et al.[79]
			METTL3	→	(m^{6}A-*MAGED / YTHDF1*)	→	MAGED1 → PCNA	Hu et al.[80]
	**PMD**	↑	PM2.5 → METTL16	→	m^{6}A-*Sulf2*	→	SULF2	Guo et al.[81]
	**Angiogenesis**	↑	ADAR1	→	(edited-CTSS / HuR)	→	CTSS	Stellos et al.[50]
			METTL3	→	mature let-7e-5p / miR-17-92 clusters ⊣ *TSP1*	⊣	TSP1	Chamorro-Jorganes et al.[56]
			METTL3	→	(m^{6}A-*DTXL3* / IGF2BP1,-3)	→	DTXL3 → DTX1/DTXL3 heterodimer	Wang et al.[106]
			WTAP, METTL3	→	(m^{6}A-*DSP* / IGF2BP1-3)	→	DSP	Wang et al.[105]
			WTAP	⊣	Wnt signaling (WT1-TBL1)	⊣	β-catenin	Wang et al.[105]
			LPS + hypoxia → ALKBH5	⊣	m^{6}A-*SPHK1*	⊣	SPHK1→ p-AKT → p-eNOS	Kumari et al.[109]
			FTO	⊣	(m^{6}A-*FAK / YTHDF2*)	⊣	FAK	Shan et al.[58]
		↓	Hypoxia → ALKBH5	⊣	m^{6}A-*Wnt5a*	→	WNT5A	Zhao et al.[104]
	**Aortic aneurysm**	↑	METTL3	→	(m^{6}A-pri-miR-34a / DGCR8)	→	miR-34a ⊣ SIRT1	Zhong et al.[86]
			ALKBH5	⊣	(m^{6}A-pri-miR-143-3p / DGCR8)	→	miR-143-3p ⊣ DDX6	Wang et al.[88]
			FTO	⊣	m^{6}A-*Klf5*	→	KLF5	Ma et al.[87]
				→	GSK3β phosphorylation (inactivation)	⊣	KLF5	
		↓	KIAA1429	→	(m^{6}A-pri-miR-143-3p / DGCR8)	→	miR-143-3p ⊣ DDX6	Wang et al.[88]

TABLE S5. Selected summary of identified small molecules targeting m^{6}A regulators.

Abbreviations: ***A375***, human amelanotic melanoma cell line (RRID:CVCL_0132); A549, human lung adenocarcinoma cell line (RRID:CVCL_0023); ***ALKBH5***, alkB homolog 5, RNA demethylase; ***AML***, acute myeloid leukemia; ***ASB2***, ankyrin repeat and SOCS box containing 2; ***CEBPA***, CCAAT/enhancer-binding protein alpha; ***CCRF-CEM***, childhood T cell acute lymphoblastic leukemia cell line (RRID:CVCL_0207); ***FTO***, FTO alpha-ketoglutarate dependent dioxygenase; ***G0***, resting phase (cell cycle), ***G1***, period from mitosis to replication (cell cycle); ***HeLa***, human papillomavirus-related endocervical adenocarcinoma cell line (RRID:CVCL_0030); ***HL-60***, human adult acute myeloid leukemia cell line (RRID:CVCL_0002); ***K-562***, adult chronic myeloid leukemia cell line (RRID:CVCL_0004); ***KNS81***, human glioblastoma cell line (RRID:CVCL_2799); ***LN229***, human glioblastoma cell line (RRID:CVCL_0393); ***LSCs***, leukemia stem cells; ***MDA-MB-231***, human breast adenocarcinoma cell line (RRID:CVCL_0062); ***METTL3***, methyltransferase 3, N^6-adenosine-methyltransferase complex catalytic subunit; ***METTL14***, methyltransferase 14, N^6-adenosine-methyltransferase subunit; ***MIA PaCa-2***, adult human pancreatic cancer cell line (RRID:CVCL_0428); ***MOLM-13***, adult acute human myeloid cell line (RRID:CVCL_2119); ***MONOMAC6***, adult human acute monocytic leukemia cell line (RRID:CVCL_1426); ***MYC***, MYC proto-oncogene; ***NB4***, childhood human acute promyelocytic leukemia cell line (RRID:CVCL_0005); ***NOMO-1***, adult human acute monocytic leukemia cell line (RRID:CVCL_1609); ***PDX-AML***, patient-derived xenograft acute myeloid leukemia; ***RARA***, retinoic acid receptor alpha; ***SOCS1***, suppressor of cytokine signaling 1.

Table S5. A selection of identified compounds targeting enzymes regulating m⁶A

Compound	Specific target	Disease / Model	Results	Reference
- STM2457	METTL3-14 inhibitor	Acute myeloid leukemia	Demethylation of leukaemogenic mRNAs inducing translational defect. Prolonged survival of various mice models of AML.	Yankova et al.[1]
- Eltrombopag	METTL3-14 inhibitor	AML cell line (MOLM-13)	Inhibited proliferation	Lee et al.[2]
- Quercetin	METTL3 inhibitor	MIA PaCa-2 Pancreatric cancer cell line	Dose-dependently decreased of m6A and inhibited proliferation	Du et al.[3]
- Methyl Piperidine-3-carboxylate Hydrochloride<br>- Tert-butyl 6-methylpiperidine-3-carboxylate<br>- Methyl 6-methylpiperidine-3-carboxylate<br>- Methyl Piperazine-2-carboxylate	METTL3-14-WTAP activators	HEK293 cells	Increased (1pM, **1-4**; 1μM; **2,4**) and decreased (10μM, **1-4**) RNA m^6A content, increase of G0/G1 cells (**1-3**), increased proliferation (**3-4**), delayed increase in DNA synthesis (**4**)	Selberg et al.[4]
- 4-amino-8-chloroquinoline-3-carboxylic acid<br>- 8-aminoquinoline-3-carboxylic acid	FTO inhibitors	Midbrain mouse dopaminergic neurons	Enhanced survival during growth factor deprivation, good penetrance through blood-brain barrier	Selberg et al.[5]
- Meclofenamic acid (MA)	FTO inhibitor	HeLa cells	Selective FTO inihibition and increased m^6A content	Huang et al.[6]
- Fluorescein derivatives	FTO inhibitor			Wang et al.[7]
- FB23 (designed MA)<br>- Benzohydroxamic acid (FB23-2)	FTO inhibitors	NB4 + MONOMAC6 cells<br><br>Mice (xeno-MONOMAC6)<br>Mice (PDX AML)	Weak effect on proliferation (low cellular uptake)<br>Anti-proliferative, prodifferentiative, proapototic, m6A increase<br>Suppressed leukemia progression and enhanced survival<br>Prolonged latency and survival, elimination of LSCs	Huang et al.[8]
- Rhein<br>- FB23-2	FTO inhibitors	Human obesity and hypertensive arteries	Increased myogenic tonus	Kruger et al.[9]
- 18077<br>- 18097	FTO inhibitors	Cancer cell lines (HeLa, MDA-MB-231, A549, A375)<br>Mice (xeno-MDA-MB-231)	Increase m6A and FTO stability, chemosensitivity, suppress proliferation, lipogenesis and invasive properties involving SOCS1<br>Smaller tumor volumes, reduced pulmonary colonization	Xie et al.[10]
- CS1 (Hydrophobic with micelles / β-cyclodextrin)<br>- CS2	FTO inhibitors	AML cell lines (multiple)<br>Mice (Leukemia stem cells)<br>NOMO-1 cells<br>Mice (PDX AML)	Reduced viability, increased apoptosis, differentiation, m6A level<br>Decreased levels, inhibited repopulation capacity<br>Decreased *MYC* + *CEBPA*, increased *RARA* + *ASB2* translation<br>Prolonged survival, reduced infiltratiton (above FB23-2)	Su et al.[11]
- FTO-4	FTO inhibitor	Patient-derived glioblastoma stem cells	Prevented neurosphere formation while no effect on neurosphere formation of healthy stem cells	Huff et al.[12]
- 2-[(1-hydroxy-2-oxo-2-phenylethyl)sulfanyl]acetic acid<br>- 4-{[(furan-2-yl)methyl]amino}-1,2-diazinane-3,6-dione	ALKBH5 inhibitors	Leukemia cells (HL-60, CCRF-CEM, K562)	Inhibited cell proliferation	Selberg et al.[13]
- Ena15<br>- Ena21	ALKBH5 inhibitors	Human glioblastoma multiforme cell lines KNS81 and LN229	Inhibited growth activity	Takahashi et al.[14]

A review discussing both FTO and ALKBH5 inhibitors identified by 22.5.2022: You et al.[15]

Supplemental references

1. Yankova, E., Blackaby, W., Albertella, M., Rak, J., De Braekeleer, E., Tsagkogeorga, G., Pilka, E.S., Aspris, D., Leggate, D., Hendrick, A.G., et al. (2021). Small-molecule inhibition of METTL3 as a strategy against myeloid leukaemia. Nature 593, 597-601. 10.1038/s41586-021-03536-w.
2. Lee, J.H., Choi, N., Kim, S., Jin, M.S., Shen, H., and Kim, Y.C. (2022). Eltrombopag as an Allosteric Inhibitor of the METTL3-14 Complex Affecting the m(6)A Methylation of RNA in Acute Myeloid Leukemia Cells. Pharmaceuticals (Basel) 15. 10.3390/ph15040440.
3. Du, Y., Yuan, Y., Xu, L., Zhao, F., Wang, W., Xu, Y., and Tian, X. (2022). Discovery of METTL3 Small Molecule Inhibitors by Virtual Screening of Natural Products. Front Pharmacol 13, 878135. 10.3389/fphar.2022.878135.
4. Selberg, S., Blokhina, D., Aatonen, M., Koivisto, P., Siltanen, A., Mervaala, E., Kankuri, E., and Karelson, M. (2019). Discovery of Small Molecules that Activate RNA Methylation through Cooperative Binding to the METTL3-14-WTAP Complex Active Site. Cell Rep 26, 3762-3771 e3765. 10.1016/j.celrep.2019.02.100.
5. Selberg, S., Yu, L.Y., Bondarenko, O., Kankuri, E., Seli, N., Kovaleva, V., Herodes, K., Saarma, M., and Karelson, M. (2021). Small-Molecule Inhibitors of the RNA M6A Demethylases FTO Potently Support the Survival of Dopamine Neurons. Int J Mol Sci 22. 10.3390/ijms22094537.
6. Huang, Y., Yan, J., Li, Q., Li, J., Gong, S., Zhou, H., Gan, J., Jiang, H., Jia, G.F., Luo, C., et al. (2015). Meclofenamic acid selectively inhibits FTO demethylation of m6A over ALKBH5. Nucleic Acids Res 43, 373-384. 10.1093/nar/gku1276.
7. Wang, T., Hong, T., Huang, Y., Su, H., Wu, F., Chen, Y., Wei, L., Huang, W., Hua, X., Xia, Y., et al. (2015). Fluorescein Derivatives as Bifunctional Molecules for the Simultaneous Inhibiting and Labeling of FTO Protein. J Am Chem Soc 137, 13736-13739. 10.1021/jacs.5b06690.
8. Huang, Y., Su, R., Sheng, Y., Dong, L., Dong, Z., Xu, H., Ni, T., Zhang, Z.S., Zhang, T., Li, C., et al. (2019). Small-Molecule Targeting of Oncogenic FTO Demethylase in Acute Myeloid Leukemia. Cancer Cell 35, 677-691 e610. 10.1016/j.ccell.2019.03.006.
9. Kruger, N., Biwer, L.A., Good, M.E., Ruddiman, C.A., Wolpe, A.G., DeLalio, L.J., Murphy, S., Macal, E.H., Jr., Ragolia, L., Serbulea, V., et al. (2020). Loss of Endothelial FTO Antagonizes Obesity-Induced Metabolic and Vascular Dysfunction. Circ Res 126, 232-242. 10.1161/CIRCRESAHA.119.315531.
10. Xie, G., Wu, X.N., Ling, Y., Rui, Y., Wu, D., Zhou, J., Li, J., Lin, S., Peng, Q., Li, Z., et al. (2022). A novel inhibitor of N (6)-methyladenosine demethylase FTO induces mRNA methylation and shows anti-cancer activities. Acta Pharm Sin B 12, 853-866. 10.1016/j.apsb.2021.08.028.
11. Su, R., Dong, L., Li, Y., Gao, M., Han, L., Wunderlich, M., Deng, X., Li, H., Huang, Y., Gao, L., et al. (2020). Targeting FTO Suppresses Cancer Stem Cell Maintenance and Immune Evasion. Cancer Cell 38, 79-96 e11. 10.1016/j.ccell.2020.04.017.
12. Huff, S., Tiwari, S.K., Gonzalez, G.M., Wang, Y., and Rana, T.M. (2021). m(6)A-RNA Demethylase FTO Inhibitors Impair Self-Renewal in Glioblastoma Stem Cells. ACS Chem Biol 16, 324-333. 10.1021/acschembio.0c00841.
13. Selberg, S., Seli, N., Kankuri, E., and Karelson, M. (2021). Rational Design of Novel Anticancer Small-Molecule RNA m6A Demethylase ALKBH5 Inhibitors. ACS Omega 6, 13310-13320. 10.1021/acsomega.1c01289.
14. Takahashi, H., Hase, H., Yoshida, T., Tashiro, J., Hirade, Y., Kitae, K., and Tsujikawa, K. (2022). Discovery of two novel ALKBH5 selective inhibitors that exhibit uncompetitive or competitive type and suppress the growth activity of glioblastoma multiforme. Chem Biol Drug Des. 10.1111/cbdd.14051.
15. You, Y., Fu, Y., Huang, M., Shen, D., Zhao, B., Liu, H., Zheng, Y., and Huang, L. (2022). Recent Advances of m6A Demethylases Inhibitors and Their Biological Functions in Human Diseases. Int J Mol Sci. 23, 5815. 10.3390/ijms2310581
