# Supplementary material for: Emerging roles of the RNA modifications N6-methyladenosine and adenosine-to-inosine in cardiovascular diseases
Source: Mol Ther Nucleic Acids. 2022 Jul 20;29:426–61. doi: 10.1016/j.omtn.2022.07.018 (PMC9366019; doi:10.1016/j.omtn.2022.07.018)
Supplement: Document S2. Article plus supplemental information [file mmc3.pdf]

Molecular Therapy
Nucleic Acids
Review

Emerging roles of the RNA modifications N6-methyladenosine and adenosine-to-inosine in cardiovascular diseases

Vilbert Sikorski,[1] Antti Vento,[2] Esko Kankuri,[1] and on behalf of the IHD-EPITRAN Consortium

[1]Department of Pharmacology, Faculty of Medicine, University of Helsinki, 00014 Helsinki, Finland; [2]Heart and Lung Center, Helsinki University Hospital, 00029 Helsinki, Finland

Cardiovascular diseases lead the mortality and morbidity disease metrics worldwide. A multitude of chemical base modifications in ribonucleic acids (RNAs) have been linked with key events of cardiovascular diseases and metabolic disorders. Named either RNA epigenetics or epitranscriptomics, the post-transcriptional RNA modifications, their regulatory pathways, components, and downstream effects substantially contribute to the ways our genetic code is interpreted. Here we review the accumulated discoveries to date regarding the roles of the two most common epitranscriptomic modifications, N^6-methyl-adenosine (m^{6}A) and adenosine-to-inosine (A-to-I) editing, in cardiovascular disease.

INTRODUCTION

Cardiovascular diseases

Cardiovascular diseases (CVDs) cause more than one-third of all deaths worldwide. Almost half of the 18.6 million people that die annually to CVDs are due to ischemic heart disease (IHD), making it the leading single cause of death.[1–2] Altogether, a total of 523 million people suffer from these diseases—including 197 million patients with IHD—and their disease burden is manifested as an annual loss of 393 million disability-adjusted life years.[2] In the United States, this translates into an annual expense of $352 billion in direct health care costs and lost productivity.[3] In the European Union, this cost is approximated as $255 billion.[4] Moreover, further contributing factors to the snowballing effect of CVDs are major other global phenomena, such as the increasing world population, westernization of life habits, and the increased proportion of aged individuals, as recently reviewed for atherosclerosis, the common underlying disease of most CVDs.[5] In more than every 10th person over 65 years of age, CVDs, IHD in particular, eventually manifest as heart failure (HF),[6] a severe syndrome associated with 5-year mortality rates of 43.3%–48.5%.[7–8] Alarmingly, the prevalence of HF in the elderly population is expected to be over 30% by the year 2030.[9]

The high morbidity and mortality attributable to CVDs have initiated massive efforts to reduce their burden. Many revolutionary inventions, such as new molecular entity drugs and biological therapies,[10–12] non-invasive imaging methods,[13–14] sophisticated endovascular interventions,[15–16] and implantable devices,[17] have helped to improve disease prognosis in terms of relative reduction in morbidity and, in some instances, mortality.

However, the fact that CVDs remain the single most fatal and morbid group of pathologies forces us to reach further. Generally, this quest is divided into stages of primary, secondary, and tertiary prevention.[18] The contemporary advances in cardiovascular medicine have predominantly concentrated on either secondary or tertiary prevention; i.e., to diagnose and treat CVDs after their earliest possible manifestation or to stall symptomatic diseases from development of further complications, respectively. Effective primary prevention of disease, on the other hand, requires identification and intervention at the level of upstream factors causally responsible for initiating the development of disease.

Atherosclerosis manifests as fatty, inflamed, and calcified deposits in the walls of arteries. It is the underlying pathologic process in most CVDs, jointly termed atherosclerotic CVDs or atherosclerotic cardiovascular diseases (ACVDs).[19] Distinct pathological entities arise based on the affected principal anatomic sites (Table 1).

Factors such as smoking, hypertension, high cholesterol, obesity, systemic inflammation, and genetics all contribute to the development of CVDs. Nevertheless, the causative factor triggering ACVD development has not yet been identified.[5,19] Hypotheses on the etiology of atherosclerosis include, for example, infectious agents,[20] as well as gut-microbiota-produced circulating metabolites,[21] such as trimethylamine-N-oxide[22] and phenylacetylglutamine.[23]

Ribonucleic acids (RNAs) constitute a critical upstream hub for cellular response control at the intersection of our genetic code and its translation. RNA is subject to multiple levels of processing, including both canonical and alternative splicing,[24] tailing,[25] and biochemical modifications.[26–27] All of these processes are not only

https://doi.org/10.1016/j.omtn.2022.07.018.

Correspondence: Esko Kankuri, M.D. Ph.D., Faculty of Medicine, Department of Pharmacology, PO Box 63 (Haartmaninkatu 8), FIN-00014 University of Helsinki, 00014 Helsinki, Finland.
E-mail: esko.kankuri@helsinki.fi

 © 2022 The Author(s).
This is an open access article under the CC BY license (http://creativecommons.org/licenses/by/4.0/).

Table 1. Main types of ACVDs with respective common abbreviations, anatomic sites, and typical clinical entities with typical symptoms

ACVD	Abbreviation(s)	Anatomic site	Typical presentation(s)
Stroke, cerebrovascular accident	None, CVA	Intracranial arteries (also thromboemboli from extracranial arteries, heart, or shunting from the venous system)	Sudden unilateral paralysis or paresthesia in any part of the body; abrupt trouble to speak or understand speech; sudden disturbance of either posture or sight (homonymous hemianopsia), sudden first-of-its-kind severe headache
Carotid artery disease	CAD	Carotid arteries	As in stroke with an addition of a relatively pathognomonic sudden unilateral loss of sight (*amaurosis fugax*)
Ischemic heart disease, coronary artery disease	IHD, CAD	Coronary arteries	*Stable:* exertion-inducible chest pain (*angina pectoris*), dyspnea, fatigue, dizziness, lower extremity edema<br>*Unstable:* abrupt pressing chest pain not relieved at rest, reflective pain in upper body, severe fatigue, dizziness, light-headedness, nausea, cold sweats, variable (malignant) arrythmias, syncope, sudden unexpected death
Aortic aneurysms	TAA, TAAA, AAA	Aorta	*Chronic/subclinical:* asymptomatic, dyspnea on exertion or in specific positions (thoracic), striking abdominal pulsating mass<br>*Acute:* dissection or rupture of the sickened aortic wall, harrowing pain across back, dizziness, nausea, syncope, massive both hyperacute and acute mortality
Mesenteric ischemia	None	Visceral arteries	*Chronic*: unwanted weight loss, diarrhea, idiopathic consistent temporary postprandial stomach pain<br>*Acute*: severe abdominal pain, nausea, vomiting, fever, organ necrosis, sepsis, high acute mortality
Peripheral artery disease, arteriosclerosis obliterans	PAD, ASO	Arteries of the lower extremity	*Chronic:* disability, vascular claudication (reduced walking distance due to ischemic muscle pain)<br>*Critical:* rest pain, ischemic ulcers, gangrenes, cold extremities, amputations<br>*Acute obstruction:* intense pain, loss of distal muscle functions and numbness, white and cold extremity

critical for governing RNA function, cellular homeostasis, and physiological responses but, when dysregulated, they also lead and contribute to the development of disease.

Epitranscriptomics and the common internal RNA adenosine modifications: m^{6}A and A-to-I

In the 1940s, Conrad Waddington introduced dynamic chemical modifications to nucleic acids, initially recognized in deoxyribonucleic acid (DNA) as epigenetic alterations.[28] However, nitrogen-5′-methylated cytosine was first discovered in 1925 in a living organism as an integral part of tuberculinic acid, a toxic noncanonical nucleic acid produced by *Mycobacterium tuberculosis*.[29] Compared with DNA modifications, the first reports regarding epitranscriptomics, or RNA epigenetics—the field of research on post-transcriptional biochemical modifications of RNA bases—were obtained decades later in the 1960s and 1970s. First, methionine-dependent methylation of pre-ribosome RNA was identified to be mandatory for its functional maturation in the HeLa cancer cell line.[30] Multiple different types of methylations in messenger RNAs (mRNAs) were first observed in the Novikoff hepatoma cell line.[31] Thereafter, due to methodological limitations, epitranscriptomic research stagnated considerably. Only the methodological breakthroughs of the last decade, first the antibody-based enrichment of methylated RNA prior to sequencing (meRIP-seq),[32–33] followed by both enzyme-based identifications[34–35] and recently base-calling algorithms coupled with third-generation direct sequencing methodologies,[36] have made the accurate characterization of some of these epitranscriptomic modifications increasingly feasible. Over 170 post-transcriptional modifications have been identified in nearly all RNA species.[37,38] However, while numerous RNA decorations have been identified, only a few have been assigned a functional role so far.

Of these, the nitrogen-6-methyl-adenosine (m^{6}A) and adenosine-to-inosine (A-to-I) RNA modification and editing, respectively, are the most common and most intensively studied.[31,39] m^{6}A has been shown to favor a consensus sequence DR(A/m^{6}A)CH.[32,40,41] (D = A,G, or U, R = A or G, and H = A, C or U). On average, three such sites are found in each mammalian mRNA molecule. A-to-I editing primarily occurs in the primate-specific ~300-nucleotide-long *Alu* sequences when such repeats align and pair after transcription to form double-stranded RNA (dsRNA) structures.[39] *Alu* sequences constitute 10% of the human genome and are enriched to gene-rich regions of the genome. The abundance and effects of these modifications are governed by designated enzyme families acting either as writers, erasers, or readers, and are summarized in Figure 1.

Writers of m^{6}A to mRNA, methyltransferases, include both methyltransferase 16, N^6-methyladenosine (METTL16), and the major writer complex that involves methyltransferase 3, N^6-adenosine-methyltransferase complex catalytic subunit (METTL3), alongside its catalytically inactive methyltransferase 14, N^6-adenosine-methyltransferase (METTL14) subunit, WT1-associated protein (WTAP),

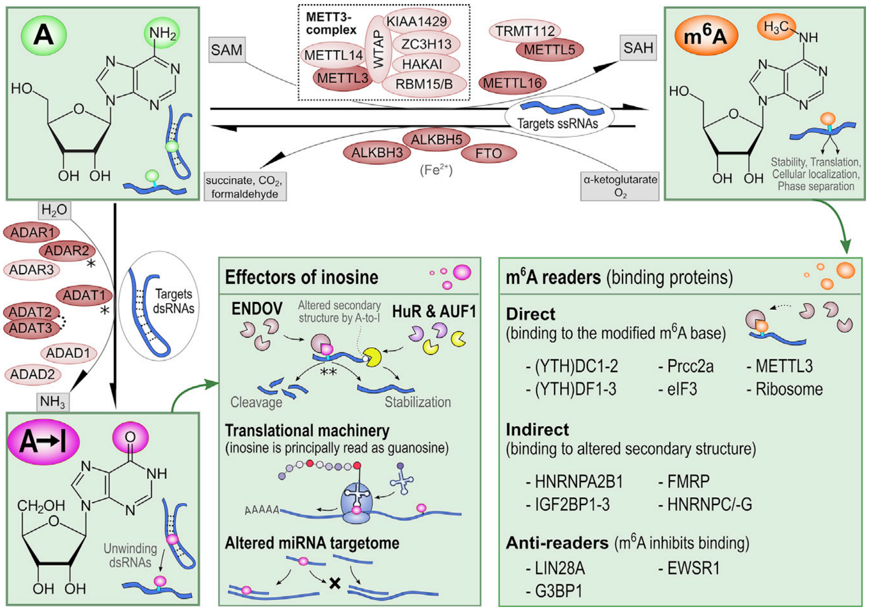

Figure 1. Depiction of the contributors responsible for A-to-I editing and m^{6}A modification, respective downstream effectors, and the key effects on RNA biology

*Inositol hexakisphosphate (cofactor). **While ENDOV has been recently suggested to protect inosine-bound transcripts from degradation *in vivo*, it acts to target them for cleavage *in vitro* (see section "atherosclerosis"). While red-colored molecules harbor catalytic activity, the light-colored molecules act as non-catalytic subunits. The abbreviations are listed within the text.

as well as their interacting partners such as vir-like m^{6}A methyltransferase-associated (VIRMA) protein, zinc-finger CCCH-type containing 13 (ZC3H13) protein, E3 ubiquitin-protein ligase hakai (HAKAI), and RNA-binding motif protein 15 (RBM15).[38] Moreover, a heterodimeric complex of methyltransferase 5, N^6-adenosine (METTL5) and tRNA methyltransferase activator subunit 11-2 (TRMT112) write m^{6}A specifically on 18S ribosomal RNAs (rRNAs).[42] During the methylation process, S-adenosyl methionine (SAM) acts as a methyl donor and converts to S-adenosylhomocysteine (SAH). To date, three m^{6}A erasers, demethylases, have been identified: widespread RNA-acting FTO alpha-ketoglutarate dependent dioxygenase (FTO) and testes-enriched alkB homolog 5, RNA demethylase (ALKBH5),[38] as well as tRNA-targeting alkB homolog 3, alpha-ketoglutarate dependent dioxygenase (ALKBH3).[43] FTO has also been described as a major eraser of N^6,2′-*O*-dimethyladenosine (m^{6}A$_m$) nucleotide and thus regulator of small nuclear RNA processing.[44] On the other hand, ALKBH3 also demethylates N^1-methyladenosines in both mRNAs and transfer RNAs (tRNAs).[45–47] ALKBH5 is currently understood as an m^{6}A-dedicated eraser principally localizing to nuclear speckles.[38] All these erasers depend on both α-ketoglutarate and molecular oxygen as co-substrates and Fe^{2+} as a cofactor. The readers of m^{6}A, crucial for mediating its downstream effects, fall into three major categories based on their principal ways of binding to m^{6}A-RNA: direct binders to the m^{6}A, indirect binders to the m^{6}A-dependently altered RNA secondary structures, and binders that are specifically repelled from their binding sites in RNA following m^{6}A deposition (Figure 1). The two m^{6}A reader families that contain an m^{6}A-binding YT521-B homology (YTH) domain; the YTH N^6-methyladenosine RNA-binding proteins 1, 2, and 3 (YTHDF1, YTHDF2, and YTHDF3, respectively); and YTH domain containing 1 and 2 (YTHDC1 and YTHDC2, respectively) constitute a major set of investigated direct readers.[38] These also include proline-rich coiled-coil 2 A (PRCC2A) protein, eukaryotic initiation factor 3 (eIF3), METTL3, and ribosomes themselves.[38] Several indirect readers have been identified: heterogeneous nuclear ribonucleoproteins A2/B1, C, and G (HNRNPA2/B1, HNRNPC, and HNRNPG, respectively); insulin-like growth factor 2 mRNA-binding proteins 1, 2, and 3 (IGF2BP1–3); and fragile X mental retardation protein (FMRP).[38] Last, the lin-28 homolog A (LIN28A), EWS RNA-binding protein 1 (EWSR1), and G3BP stress granule assembly factor 1 (G3BP1) have been described to be repelled from their RNA-binding site following m^{6}A methylation.[38]

In vertebrates, A-to-I editing is carried out by three families of deaminases acting on dsRNA: ADAR (adenosine deaminase RNA specific) family in all tissues, ADAD (adenosine deaminase domain-containing) family principally in testes or brain and ADAT (tRNA adenosine deaminase) family solely targeting tRNAs.[27] While no cofactors for these writers have been identified, inositol hexakisphosphate has been shown to complex within the enzymatic core of adenosine deaminase RNA-specific B1 (ADAR2) and thus to be imperative for its (as well as proper editing function of ADAT1 [adenosine deaminase tRNA specific 1]).[48]

While the ADAD family contains two members, *ADAD1* (adenosine deaminase domain containing 1) and *ADAD2* (adenosine deaminase domain containing 2), the ADAR family consists of three members: *ADAR1* (adenosine deaminase RNA specific), *ADAR2*, and *ADAR3* (adenosine deaminase RNA specific B2 [inactive]). Only ADAR1 and ADAR2 proteins have catalytic activity.[27] *ADAR1* gene is transcribed from two start sites to produce two N-terminally distinct isoforms, a longer and interferon (INF)-inducible ADAR1 p150 and a shorter constitutively expressed ADAR1 p110 isoform. *ADAR2* mRNA can undergo extensive alternative splicing in a tissue-specific manner.[49] All ADARs can directly bind dsRNA. For effective deamination, ADAR1 and ADAR2 undergo homodimerization. However, ADAR3 cannot homodimerize, which has been postulated as a reason for its lack of A-to-I editing activity.[27] ADAR2 is predominantly localized to the nucleus, but the ADAR1 isoforms exhibit specifically regulated nucleocytoplasmic shuttling.[27]

No enzymes converting inosine back to adenosine have been described. However, human antigen R (HuR), or ELAV-like RNA-binding protein 1 (ELAVL1), inosine-dependently binds RNA,[50] and endonuclease V (ENDOV)[51] has been reported to cleave specifically at highly inosine-modified *Alu* sequences functioning thus as readers or effectors.

Current literature assigns diverse functions to m^{6}A ranging from regulation of RNA secondary structures,[39] stability,[52] translation efficiency,[53] compartmentalization, and degradation[39] to regulation of proliferation,[54,55] motility,[56–58] paracrine signaling,[59] phenotype,[60] and cell fate decisions.[61] In addition, m^{6}A RNA has been implicated as a critical contributor to numerous pathologies, including cancer, immunological and metabolic diseases, as well as CVDs.[62,63] Indeed, m^{6}A has emerged as a tissue- and context-specific hub that mediates cellular stress responses, as recently reviewed.[64] Also, A-to-I modifications participate in a multitude of RNA-related processes, including RNA stability, secondary structure and accessibility modifications, exon and intron editing, and both microRNA (miRNA) maturation and subsequent target specifications.[27,65–68] The formed inosines are capable of altering the RNA secondary structure by disrupting the Watson-Crick base pairing to unwind the dsRNAs and form more immune-tolerable single-stranded RNAs (ssRNAs).[27] Indeed, ADAR1 deficiency has been linked with accumulation of intracellular dsRNAs, activation of interferon production, and various auto-inflammatory diseases.[27] A-to-I editing has also proved essential for the maintenance of hematopoiesis and has been linked with regulation of innate immune responses,[69] development of cancer,[70] and maintenance of neurologic functions.[27]

N^6-METHYLADENOSINE AND A-TO-I MODIFICATIONS IN CARDIOVASCULAR DISEASES

We begin this section by discussing RNA m^{6}A and A-to-I modifications in heart development and regeneration. Next, we move on to hypertension in its various forms and its most common cardiac complications, cardiac hypertrophy, and HF. We then discuss m^{6}A and A-to-I modifications in atherosclerosis, myocardial ischemia, hypoxia, fibrosis, and angiogenesis. The concluding sections consider the accumulated observations regarding aortic valve calcification and aortic aneurysms.

Figure 2 offers an overall summary of studies that have assessed either m^{6}A or A-to-I RNA modifications in cardiovascular development, physiology, or disease.[50,54–58,60,65–68,71–166,171,175] Figures 3 and 4 offer more detailed mechanistic summaries of the molecular interactions and pathways involving m^{6}A and A-to-I modifications within pathophysiology of the most common vasculopathies and according to IHD pathophysiology toward HF, respectively. The current understanding of molecular pathways involved in obesity and diabetic cardiomyopathy is presented in Figure 5, in atherosclerosis in Figure 6, and pathways involved in monocyte/macrophage activation, inflammation, and foam cell formation in Figure 7.

The key observations on the roles of m^{6}A modification and A-to-I editing in the cardiovascular system are listed in Tables S1 and S2, respectively. Table S3 further details the interventional results regarding m^{6}A regulators in CVD models. Finally, Table S4 provides a molecular-level view into the known interactions and pathways involving the epitranscriptomic m^{6}A and A-to-I modifications in cardiovascular disease.

Cardiogenesis and cardiac regeneration

Heart development begins early during organogenesis, and a four-chamber heart is already established at weeks 5–8 of gestation.[167] While in adult mammals the heart grows in size through hypertrophic adaptation and increased cell volume, in cardiogenesis the cardiomyocyte precursors proliferate and increase in number before differentiating into mature cardiac tissue.

A-to-I editing

The global knockouts of either *Adar1*$^{-/-}$ or its cytosolic isoform *Adar p150*$^{-/-}$ are nonviable due to multiple organ failures and massive global apoptosis dominating especially in the heart.[69,168–170] Cardiomyocyte-directed ADAR1 knockouts also die because of massive cardiomyocyte apoptosis.[161] On the other hand, ADAR p110 has been shown to be redundant for the viability of human embryonal stem cells.[171] Deletion of either the dsRNA sensor, a melanoma differentiation-associated protein 5 (MDA5), or its downstream effector, a mitochondrial antiviral-signaling protein (MAVS), can extend the survival of *Adar1*$^{-/-}$ mice to an immediate postpartum period. Moreover, no cardiac abnormalities were reported in the double-knockout *Adar1*$^{-/-}$ *Mavs*$^{-/-}$ mice. Hence, ADAR1-induced and A-to-I editing-mediated unwinding of dsRNAs seem to act as a cardiomyocyte survival pathway by keeping the dsRNA-triggered INF–MDA5–MAVS–endoplasmic reticulum (ER) stress-axis activation downstream at bay.[172–175] El Azzouzi et al. demonstrated that knocking down ADAR1 in a cardiomyocyte-specific manner after birth induced a robust unfolded protein response (UPR)-dependent cardiomyocyte apoptosis and ventricular remodeling, which culminated in rapidly deteriorating cardiac contractile function and death.[130] In light of the above findings and considering activation of ER stress response and UPR is central in not only IHD,[176,177] HF,[178,179] but also in CVDs in general,[180] the contribution of ADAR1 p150 in controlling the MDA5–MAVS–INF-axis, ER stress, and activation of UPR in the myocardium warrants further investigation.

Unlike ADAR1, ADAR2 appears redundant for cardiogenesis. *Adar2*$^{-/-}$ mice selectively retaining A-to-I modifications only in glutamate ionotropic receptor AMPA type subunit 2 (*GluA2*) mRNA, mandatory for murine embryogenesis and immediate postpartum development,[181,182] had no alterations in heart morphology, relative weight, blood pressure, or atrial natriuretic peptide expression.[132,162] Although the functional role of ADAR3 remains to be characterized in cardiogenesis, its expression in the heart greatly diminishes after birth.[171]

Interestingly, ADAR1 expression is upregulated in the regenerating hearts of tailed amphibians, and the protein is translocated from

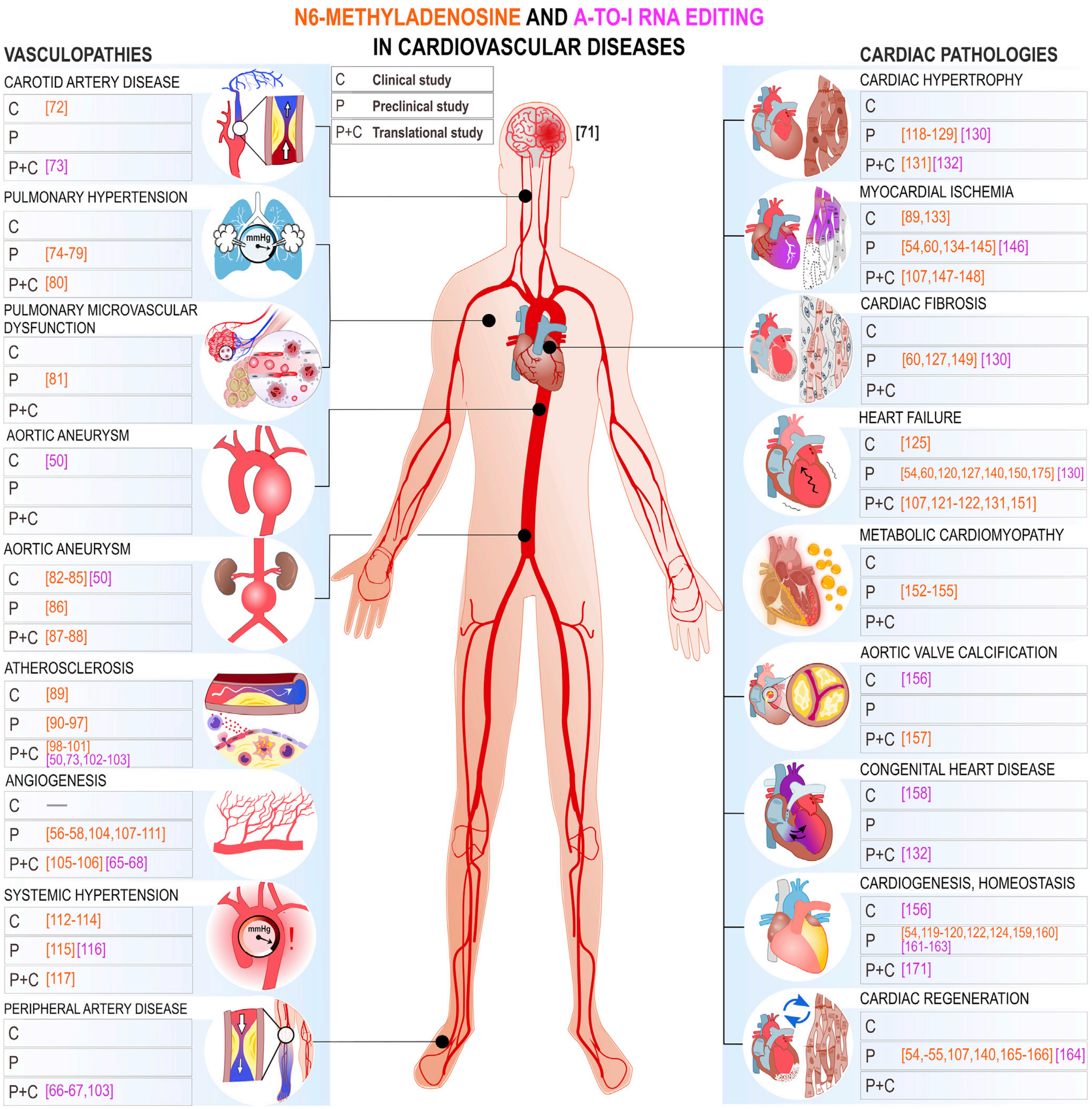

Figure 2. A schematic overview of the studies assessing m^{6}A modification and A-to-I editing in CVDs to date

Colored numbers denote specific original publication reference. The black-colored reference[71] forwards interested readers to a recent review specifically discussing the role epitranscriptomic modifications in brain physiology and diseases, which is out of topic of the present review.

the nucleus to the cytoplasm.[164] Moreover, in ADAR1 knockouts, the ability for cardiac regeneration is lost.[164] In human nerve cells *in vitro*, analogous exportin-5-dependent nucleus-to-cytoplasm translocation of ADAR p110 (a mammalian counterpart for newts' ADAR1[164]) is controlled through its phosphorylation by MKK6–p38–MSK1/2 kinases (MAP kinase kinase 6–p38 kinase–mitogen- and stress-activated protein kinases 1 and 2).[183] In the cytoplasm, ADAR p110 then acts as a stress-response mediator preserving antiapoptotic mRNAs from Staufen1-mediated degradation by editing their dsRNA segments.[183] As such, the role of MKK6–p38–MSK1/2–ADAR

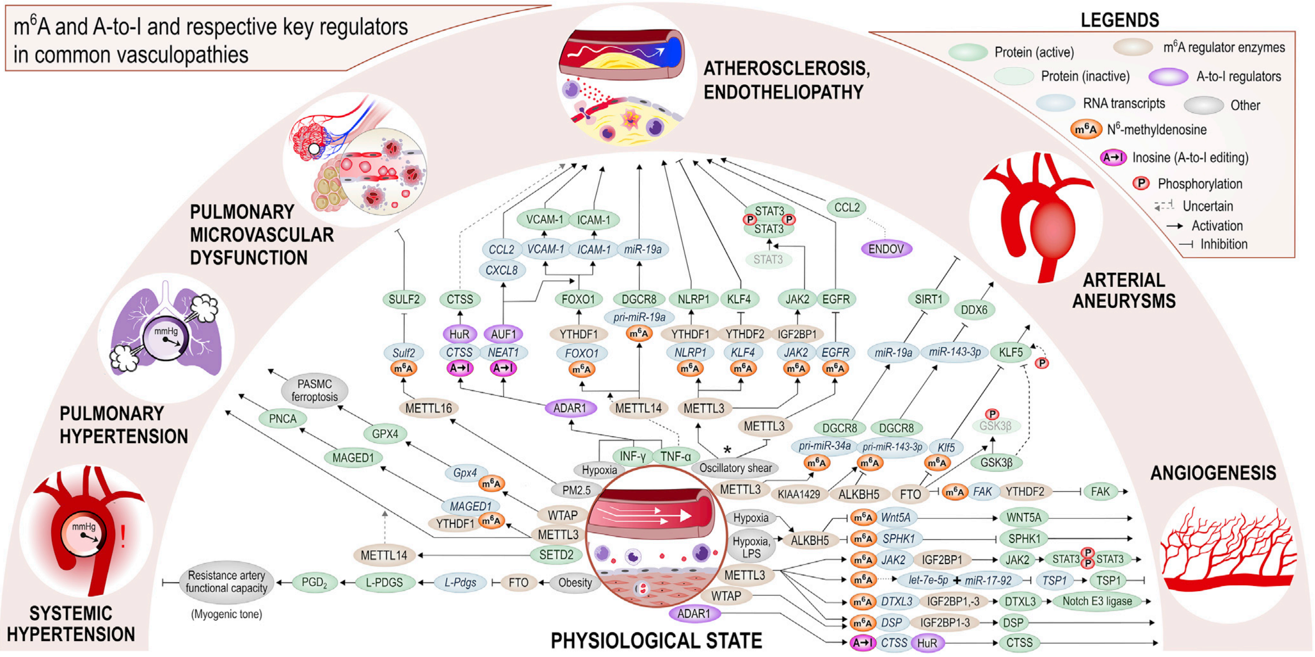

Figure 3. The unveiled molecular interactions involving m^{6}A and A-to-I or respective key regulators in common vasculopathies and non-malignant angiogenesis

The number of blunted arrows for a given pathway can be used as a guide for assessing the overall effect of the pathway. *METTL3 has been described both as proatherogenic and antiatherogenic factor in endothelium subjected to oscillatory shear stress, see later discussion in section "atherosclerosis." **The direct role of m^{6}A upregulating the respective downstream miRNAs remains putative. The role of m^{6}A and A-to-I editing in atherosclerosis pathophysiology is presented in greater detail in Figures 6 and 7. References are listed within Table S4 according to molecular pathways illustrated here. PM2.5, fine particulate matter, diameter <2.5 μm; SULF2, sulfatase 2.

p110–Staufen1 merits further investigation as a putative mechanistic regeneration target pathway. In humans, ADAR p110 expression is enriched in the atria,[132] and its expression is increased most in congenital septal defects.[132]

ADAR2 also appears to be a tentative target to instigate cardiac regeneration as its overexpression stimulates proliferation and suppresses apoptosis in rat cardiomyocytes.[146] Regarding a putative underlying molecular mechanism, ADAR2-mediated pri-miR-34a editing, which inhibited the formation of mature miR-34a via a yet veiled mechanism, induced an upregulation of its downstream proliferation-related targets Sirtuin1, Cyclin D1, and B-cell leukemia/lymphoma 2 (Bcl2) protein.[146] The negative regulation of *Adar2* promoter was suggested to be due to binding of transcription factor CCAAT/enhancer-binding protein β (C/EBPβ).[146] As discussed later, these effects were later recapitulated in a model of myocardial infarction (MI) *in vivo*.

m^{6}A modification

Akin to A-to-I editing, m^{6}A has been shown to be imperative for embryogenesis.[61] Without the m^{6}A writer METTL3, embryonal[184] and hematopoietic stem cells (HSCs)[185] lose their self-renewal ability and accumulate cytosolic dsRNA (albeit contrasting roles have also been reported[186]). No such similarity between these modifications is seen during cardiogenesis or imminent postnatal growth. Cardiomyocyte-specific METTL3-knockout mice demonstrate no signs of altered cardiac histopathology, hypertrophy, or dysfunction up to 3 months after birth.[120] At 8 months of age, however, they develop dilated, relatively thin-walled hearts (eccentric hypertrophy), cardiac dysfunction, and major lethality, a classic pathophenotype of dilated cardiomyopathy (DCM).[120] *Mettl14*$^{+/-}$ mice have also demonstrated with normal cardiac structure and function at 10 weeks of age.[143] Nonetheless, some focused m^{6}A activity appears indispensable for postnatal cardiac development as heart-specific conditional knockout YTHDC1 m^{6}A reader protein has been described to result in premature death of mice at 2–3 months of age due to disrupted m^{6}A-dependent splicing of *Titin* pre-mRNA, accompanied by destructed sarcomere organization, DCM, and ultimately HF.[150] On the other hand, cardiogenesis and postnatal development seem to proceed normally in knockout mice lacking YTHDF1,[150,187] YTHDF2,[188] YTHDF3,[150] ALKBH5,[54,189] or either global[119] or cardiomyocyte-targeted FTO knockout.[122] *In vitro*, however, YTHDF1 promotes embryonic stem cell (ESC)-derived cardiomyocyte differentiation, and YTHDF3 preserves their pluripotency via a mechanism that seems unrelated to the established key transcriptional regulation pathway including transcription factors nanog homeobox (NANOG), SRY-box transcription factor 2 (SOX2), and POU class 5 homeobox 1 (POU5F1).[160] The expression of METTL3 and METTL14, as well as the abundance of m^{6}A in RNAs, are evenly distributed in embryonic hearts, and their expression is increased by the histone deacetylase inhibitors valproic acid and Trichostatin A.[159]

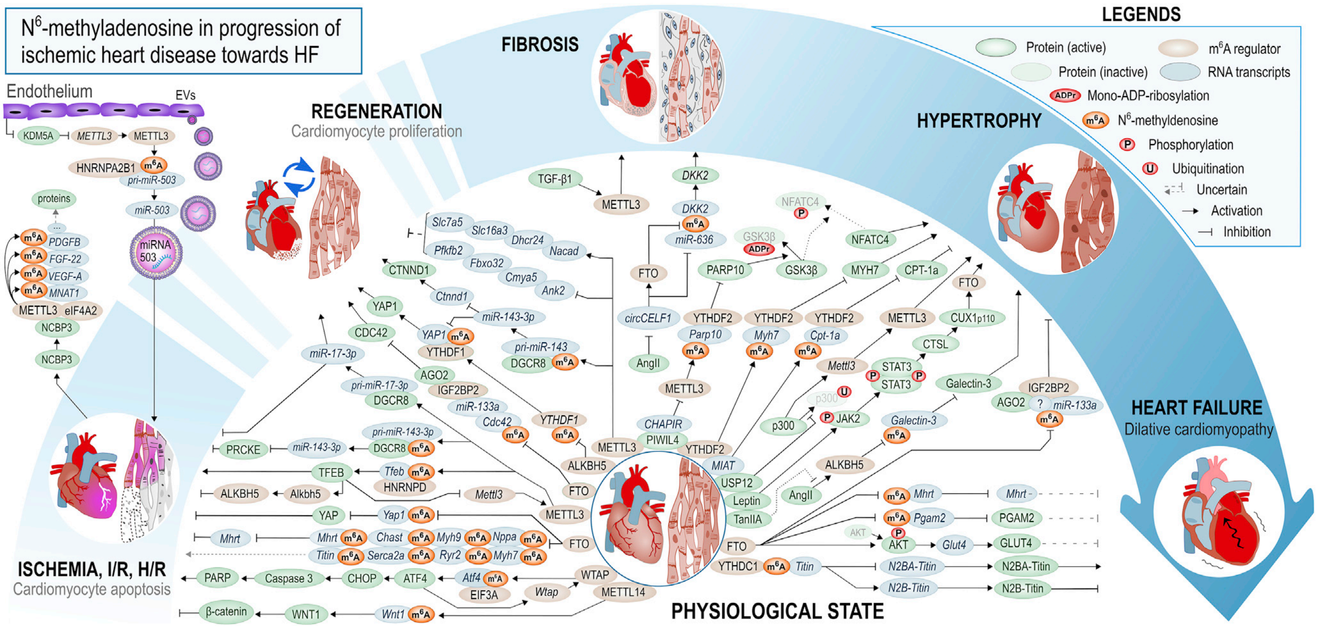

Figure 4. The unveiled molecular interactions involving m^{6}A modification or its key regulators according to various stages of IHD pathophysiology
The number of blunted arrows for a given pathway can be used as a guide for assessing the overall effect of the pathway. The break within the blue rounded arrow represents the putative regenerative ability of adult mammals (in rodents and perhaps in humans, the relevant ability for myocardium to regenerate is lost within the first week of life). References are listed in Table S4 according to molecular pathways illustrated here. AGO2, argonaute RNA-induced silencing complex (RISC) catalytic component 2; CHOP, C/EBP homologous protein; CTNND1, catenin delta 1; CTSL, cathepsin L; KDM5A, lysine demethylase 5A; MYH9, myosin heavy chain 9; NPPA, natriuretic peptide A; SLC7A5, solute carrier family 7 member 5.

The robust cardiac regenerative ability observed in rodents diminishes rapidly during the first week after birth.[190,191] Within the first postpartum week in C57BL/6J mice, the mRNA m^{6}A content has been measured to triple, METTL3 and YTHDF1 to upregulate, and the levels of *Igf2bp1*, *Igf2bp3*, *Alkbh5*, ALKBH5, FTO, and IGF2BP3 to reduce.[54,165,166] In friend leukemia virus B (FVB)-background mice, however, myocardial total RNA m^{6}A content has been reported unchanged all the way from embryonic day 14.5 (E14.5) to 12 months of age, with concurrent, and contrary to the above, upregulation of only FTO, which suggests FTO is the main m^{6}A eraser of adult mice myocardium.[124] Similarly, the adult human myocardium-extracted cardiomyocytes express FTO over the other m^{6}A regulators.[107] In rats, the myocardial METTL3 expression and stromal ALKBH5 and FTO expressions decrease during this time, accompanied by a reduction in total RNA m^{6}A content.[55]

Interestingly, the systematic mapping of mRNA m^{6}A methylome in C57BL/6J-background mice myocardium during the first month after birth exhibited 4,961 m^{6}A peaks in mRNAs from 3,062 annotated genes on their first day postpartum (P1) with corresponding numbers at a week (P7) and a month (P28) after birth soaring to 19,389 and 13,201 peaks in 7,404 and 5,721 genes, respectively.[165] While only 0.26% and 0.12% of the original m^{6}A peaks at P1 were conserved at P7 and P28, of the peaks measured at P28, 76.8% were already present at P7. Yang and colleagues characterized methylated m^{6}A-enriched mRNAs and long non-coding RNAs (lncRNAs) from the P0 and P7 rat myocardia and, well in line with reduced METTL3 and total m^{6}A content, up to 1,553 m^{6}A-peaks were identified downregulated (440 downregulated genes), but only 84 upregulated (520 upregulated genes).[55] Overall, the number of m^{6}A peak differences during P1–P7 in rats appear considerably less than those noted in mice myocardium within the same time frame.[165] Taken together, these observations suggest a major, and thus probably coordinately regulated, reorganization of the murine myocardial m^{6}A methylome concurrent with the closure of the cardioregenerative window during the first week after birth. Future investigations might elucidate both the mechanistic and functional implications of such methylome reorganization, as they may provide novel avenues to rewire the heart's ability to regenerate also in adulthood.

Mechanistically, Han et al. demonstrated that the actions of ALKBH5 and the YTHDF1 reader converge to promote yes-associated protein 1 (YAP1) expression,[54] a downstream nuclear effector of the Hippo signaling pathway stimulating cardiomyocyte proliferation.[192,193] In detail, while cardiomyocyte-specific ALKBH5 knockouts presented with reduced regenerative ability at P21 after P1 apex resection with concomitant hypertrophy and reduced cardiomyocyte proliferation, overexpression in both P7 and adult mice enhanced regeneration and functional recovery after MI.[54] Intriguingly, m^{6}A and YTHDF2 are both crucial for mitotic cytokinesis in mice oocytes.[188] Moreover, an YTHDF family orthologue has been described to restrict endocycling in the plant

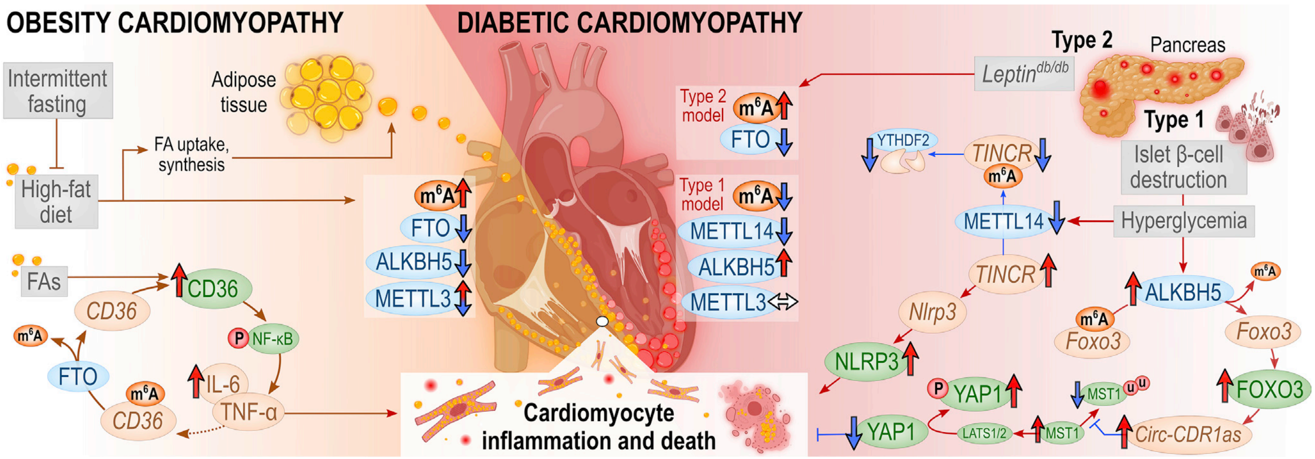

Figure 5. Summary of accumulated discoveries regarding m^{6}A and its key regulators in obesity-related and diabetic cardiomyopathy, cardiomyocyte inflammation, and death

Red upward arrows indicate upregulated expression, red horizontal arrows indicate activation, blue downward arrows denote downregulated expression, and blunt-end arrows indicate inhibition. Brown, green, and blue ellipses denote RNAs, proteins, and m^{6}A regulators, respectively. Red ellipse: "p" denotes phosphorylation, "u" ubiquitination. FAs, fatty acids; IL-6, interleukin-6.

kingdom.[195,196] These are both links to processes opposing polyploidy, which is considered a roadblock for the re-entry of adult cardiomyocytes to the cell cycle, a process associated with cardiac regeneration.[197] The ALKBH5–m^{6}A–YTHDF1–Hippo–YAP1 pathway,[54] and regulation of Hippo-mediated S-phase kinase associated protein 2 (Skp2),[198] can link these associations with suppression of polyploidy, cytokinesis failure,[199] and thus enhanced cardiac regeneration and cardiomyocyte proliferation after ischemia.[200] Interestingly, downregulation of IGF2BP3, another RNA m^{6}A-binding reader that also controls the cell cycle regulator MYC proto-oncogene via m^{6}A-dependent respective mRNA stabilization,[201] was observed in cardiac transcriptome profiling of regeneration-competent P1 and regeneration-compromised P8 mice, and was linked with modulated innate immune responses.[166] Its overexpression, on the other hand, extended this 1-week cardioregenerative window.[166] The molecular targets responsible for this IGF2BP3-enhanced regenerative ability were not studied.

Like ALKBH5, overexpression of the FTO m^{6}A eraser has also been associated with improved myocardial regeneration in mice.[107] Mathiyalagan et al. reported that FTO overexpression could salvage viable myocardium, increase angiogenesis, and preserve cardiac function after MI.[107] They observed a 96-fold hypermethylation of myocardial periostin mRNA, an integrin ligand supporting cell motility and migration.[202] Intriguingly, prior research has implicated periostin not only to act as a regenerative cardiac mitogen[203] but also to upregulate following MI when simultaneously treated with a regeneration-promoting epicardial patch encasing atrial appendage micrografts.[204] However, it has been also suggested to be a profibrotic mediator in ischemic heart.[205–207] Mechanistically, periostin has further been shown to be regulated upstream by the interleukin-13–Janus kinase–signal transducer and activator of transcription 3 (IL-13–JAK–STAT3) pathway in regenerating neonatal mice hearts.[208] Combined, it can be speculated that m^{6}A hypermethylation of periostin mRNA may promote its stability in ischemic myocardium, but this requires experimental verification. Closing the circle back to the FTO, JAK–STAT3 has been demonstrated to induce nuclear FTO upregulation to ultimately promote cardiomyocyte hypertrophy, as later described in more detail.[118]

Silencing of METTL3 in neonatal rat cardiomyocytes blocked their proliferation and altered the stability of several mRNAs. Of these, ankyrin 2, cardiomyopathy associated 5 (*Cmya5*) (associated also with muscle regeneration[194]), F-box protein 32 (*Fbxo32*), and 6-phosphofructo-2-kinase/fructose-2,6-biphosphatase 2 (*Pfkfb2*) mRNAs were stabilized, while 24-dehydrocholesterol reductase (*Dhcr24*), NAC alpha domain containing (*Nacad*), and solute carrier family 16 member 3 (*Slc16a3*) mRNAs were destabilized within hours after METTL3 silencing.[55] In line with this, METTL3 overexpression has recently been unveiled to also promote neonatal rat cardiomyocyte proliferation after hypoxia and to ameliorate ischemic myocardial damage in adult rats by promoting pri-miR-17-3p maturation in a m^{6}A-DGCR8 microprocessor complex subunit-(DGCR8)-dependent manner.[147]

On the other hand, in mice, global METTL3 knockout has been described to enhance regeneration-related markers and enhance cardiac function after MI via m^{6}A-dependently inhibited pri-miR-143-3p maturation.[140] The muscle-specific cardiac miRNA, miR-133a, was found to harbor a complementary motif CCUG for the DR-m^{6}A-CH m^{6}A consensus sequence within its seed sequence, thus making it exquisitely prone to bind m^{6}A-modified mRNAs.[124] The m^{6}A-dependent targets for this m^{6}A-oriented miRNA include the cardiomyocyte proliferation regulating cell division cycle 42

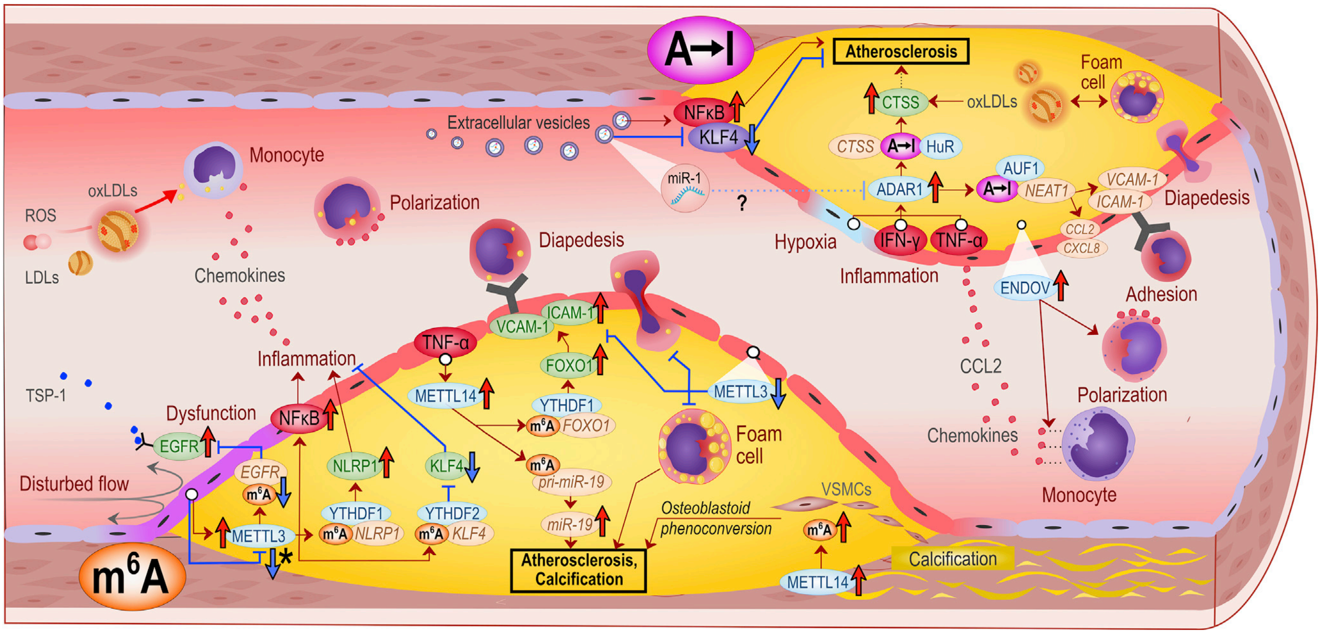

Figure 6. A summary of the key discoveries regarding adenosine-targeted epitranscriptomic alterations in atherosclerosis and arteriosclerosis to date
Red upward arrows indicate upregulated expression, red horizontal arrows indicate activation, blue downward arrows denote downregulated expression, and blue blunt-end arrows indicate inhibition. . Brown, green, and blue ellipses denote RNAs, proteins, and m^{6}A regulators, respectively. Question mark represents a putative connection based on evidence from other than atherosclerotic tissues. *METTL3 has been associated with contrasting functions and expression responses in a model of early atherosclerosis with endothelial oscillatory shear stress. See section "atherosclerosis" for further discussion. The abbreviations are listed within the text.

(*Cdc42*) mRNA in its three prime untranslated region (3′UTR).[124,209] An FTO-regulated m^{6}A- and IGF2BP2-dependent increase in miR-133a repression of *Cdc42* mRNA was shown to inhibit mouse neonatal cardiomyocyte proliferation.[124] Interestingly, the myocardial expression of miR-133a increases notably at 1 week after birth, at time of closure of the mouse regenerative window.[124]

Taken together, while overexpression of m^{6}A erasers has been shown to increase cardiomyocyte proliferation, preserve myocardial function, and promote cardiac regeneration, the role of the METTL3 m^{6}A writer in these processes appear more complex. While METTL3 knockout decreases RNA m^{6}A content and promotes regenerative cardiac healing in mice, similar to eraser overexpression,[140] the role in rats appears to be the opposite.[55,147] It is clear that these findings stress the need for species-specific considerations, but further efforts to identifying downstream responsive molecular pathways for potential therapeutic intervention to promote cardiac regeneration are also warranted.

Congenital heart disease

Developmental heart malformations are found in approximately 0.8% of births.[210] From whole-blood-derived RNA extracts collected from children with congenital heart disease and cyanosis, Borik et al. linked increased A-to-I levels of mediator complex subunit 13 (*MED13*) mRNA with reduced ADAR2 expression.[158] MED13 is associated with hypertrophy and angiogenesis, and is regulated upstream by miR-208, which is abundantly expressed in the heart.[211] miR-208 has been further described as a promising target for therapeutic inhibition in failing heart[212] and crucial for cardiac expression of GATA-binding protein 4 (GATA4),[213] a well-established transcription factor regulating cardiomyocyte phenotype, cardiogenesis, and regeneration.[214] In fruit flies, loss-of-function mutation of ADAR abates their ability to survive for hours in severe hypoxia due to impaired editing of various central nervous system (CNS)-expressed ion channel mRNAs.[215] Moreover, ADAR2 is repressed during mammalian CNS hypoxia.[216] Combined, increased A-to-I editing of blood *MED13* mRNA might represent a coping mechanism for cyanosis in children with congenital heart disease. Further, the repression in ADAR2 expression could offer access to more editing sites for the ADAR1, thus providing a possible explanation for the increased editing in *MED13* but concomitantly reduced ADAR2. *MED13* mRNA has later been shown to undergo variable transcript site-specific A-to-I editing within the transcript's *Alu* repeat in a lymphoblastoid hypoxia cell model.[217] Consistent with the above findings, a markedly reduced expression of ADAR2 (~90%) and up to 8-fold increases in both ADAR1 p150 and p110 isoforms were reported in the blood cells of children suffering from either cyanotic or acyanotic congenital heart disease.[132] Remarkably, based on mRNA expression analyses from samples derived from the Genotype-Tissue Expression (GTEx) project, the same study also measured both *ADAR1 p150* and *p110* isoforms to be upregulated 2- to 14-fold and *ADAR2* to be markedly downregulated (~75%–95%) specifically within human

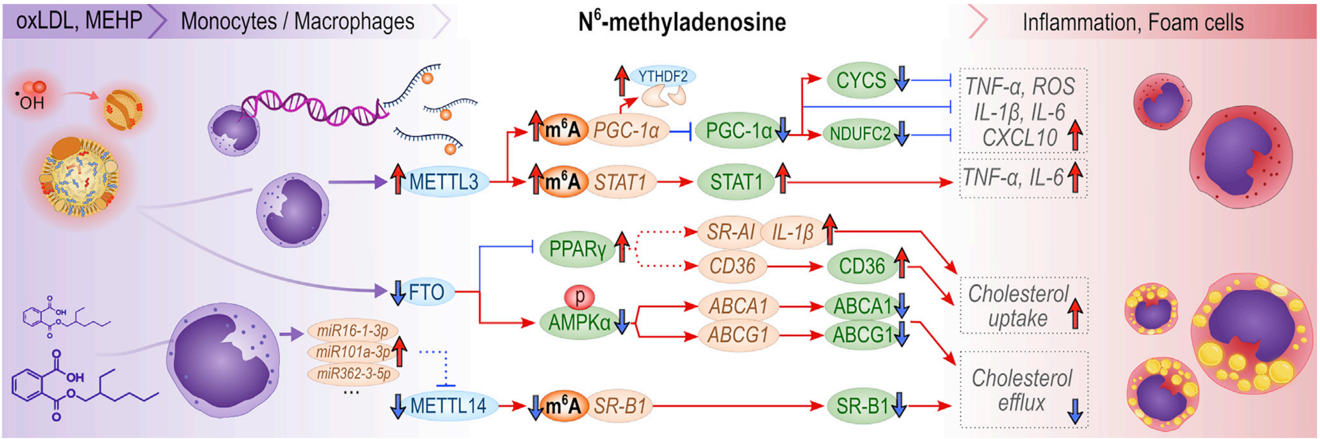

Figure 7. Currently known molecular mechanisms involving m^{6}A and its key regulators during macrophage inflammation and foam cell formation

Red upward arrows indicate upregulated expression, red horizontal arrows indicate activation, blue downward arrows denote downregulated expression, and blue blunt-end arrows indicate inhibition. Dashed line represents putative relationship. Brown, green, and blue ellipses denote RNAs, proteins, and m^{6}A regulators, respectively. Red ellipse denotes phosphorylation. ABCA1, ATP-binding cassette subfamily A member 1; ABCG1, ATP-binding cassette subfamily G member 1; AMPKα, AMP-activated protein kinase α; CXCL10, C-X-C motif chemokine ligand 10; PPAR-γ, peroxisome proliferator-activated receptor γ; SR-A1, scavenger receptor class A member 1; STAT1, signal transducer and activator of transcription 1.

hearts in variable congenital heart diseases. The most pronounced upregulations have been found in different septal defects.[132]

Cardiovascular homeostasis

Regulation of cardiovascular homeostasis is crucial due to its absolute necessity for complex mammalian life. In humans, regulatory tracts from high- and low-pressure chemo- and baroreceptors converge upstream in the medulla to signal through sympathetic nerves and the cardiac plexus to both the heart[218] and its vessels.[219] The endocrine and paracrine regulation dominantly comprise myocardium-secreted natriuretic peptides,[220] renin-angiotensin-aldosterone axis,[221] pituitary antidiuretic hormone, and oxytocin,[222] as well as catecholamines from adrenal medullae.[223] While the sinus node governs autonomous cardiac contractions,[224] the cardiac sarcomeres provide further functional contributions by modulating their contractility based on their level of stretching.[225] Moreover, arterial flows are autoregulated in several organs, including brain[226] and kidneys,[227] to ensure stable flow of oxygen and nutrients despite otherwise varying systemic blood pressure.

m^{6}A modification

The expression levels of m^{6}A writers and erasers in heart have been reported to differ across species and according to age.[107,122,124,134] However, a study specifically aiming to characterize the murine baseline cardiac distribution of the various m^{6}A writers observed a prominent downregulation of METTL3 and METTL14—and abolished METTL16—expressions within adult myocardium compared with the embryonic state.[159] Interestingly, single-cell sequencing has unveiled the m^{6}A writing complex's subunit WTAP to be widely expressed within adult human heart with highest enrichment within myocardial endothelium.[133] Moreover, the m^{6}A eraser FTO seems to hold the highest expression levels of the core m^{6}A governing enzymes within both human and murine myocardium.[107,122,124] The abundance of m^{6}A-methylated RNAs in the human myocardium (14.6%, 1,239 modified transcripts) is less than that in adult mice (24.1%, 3,208 modified transcripts).[121,122] Moreover, myocardial m^{6}A residues are potently—up to 10-fold—enriched on mRNAs compared with total RNA.[107,120] For the sake of perspective, in adult pig livers,[228] mice brain,[229] and isolated basal skin progenitor cells,[230] corresponding fractions of m^{6}A-methylated mRNAs have measured 33% (∼1.3 residues/modified gene, 4,339 modified transcripts), 53%–83% (∼1.8–2.4 residues/gene, 704–1,392 modified transcripts), and 11,420 modified transcripts (∼13.8 residues/modified transcript), respectively. These findings suggest that the activity of myocardial m^{6}A erasers dominate over that of the m^{6}A writers in the human adult heart. Such postulation is further supported when considering the preceding stoichiometric estimations suggesting each mRNA to harbor ∼1–3 sites for m^{6}A DR(A/m^{6}A)CH consensus sequence and residues as well.[41,42,231] Interestingly, the myocardial m^{6}A residues are enriched within the translation end-sites within coding sequence (CDS) and in the beginning of the 3′UTR,[122] a key region for translational control.[232] Although there are considerable differences in the nature of these methylated transcripts between humans and mice, they generally associate with such pathways as cardiogenesis, vasculogenesis, and energy deprivation-related oxidation.[122]

As myocardial m^{6}A modifications correlate poorly with the overall transcript abundance in the physiological state,[122] the role of m^{6}A readers is emphasized. Indeed, following METTL3 overexpression, there is an overall increase in cardiomyocyte transcriptome m^{6}A content, which induces contrasting effects in terms of transcript stability at level of single transcripts. Namely, both decreased (rho guanine nucleotide exchange factor 3 [*Arhgef3*]) and increased (myosin light chain 2 [*Myl2*]) mRNA transcript half-lives have been measured.[121]

Similarly, there are variable effects on transcript stability at the single-transcript level in response to METTL3 silencing.[55] Hence, a better understanding is warranted regarding the still considerably veiled functions of the m^6A readers, such as the YTHDF family and the highly expressed IGF2BP2 within baseline myocardium.[233]

A-to-I editing

As discussed above, ADAR1 is imperative for adult cardiac homeostasis, as conditional ADAR1 knockout induces 60% mortality within 3 weeks after knockout induction due to severe cardiac dysfunction with both ER stress and UPR activation as partial underlying mechanisms to the phenotype.[130] Both ADAR p110 and ADAR2 are enriched in the atria.[132] In a comprehensive comparison panel of tissue expressions, ADAR p110 was measured high in the nervous system and ADAR p150 dominated in vascular tissues, including aorta, and coronary as well as tibial arteries.[132] ADAR2 is enriched in arterial tissues[132] and its expression is reduced in various congenital cardiac malformations.[132] In *Adar2*$^{-/-}$ mice myocardia, rescued from embryonic lethality via introduction of a pre-edited *GluA2* mRNA,[181,182] multiple heart-related miRNAs were downregulated, but ADAR1 expression was not induced.[132] The most repressed miRNAs were miR-29b, miR-451b, and miR-451a, leading to increased transcription of genes including collagen type I alpha 2 chain (*Col1a2*) and insulin-like growth factor 1 (*Igf-1*).[132] Moreover, based on the decreased A-to-I editing rate of myocardial filamin B, the authors hypothesized filamin B editing to play a still-hidden function in cardiovascular system,[132] similarly as filamin A (FLNA) has been unveiled in hypertension.[116] ADAR2 has been measured with identical expression levels in *ex vivo* extracted cardiac fibroblasts and cardiomyocytes.[146]

Hypertension

Hypertension, or sustainedly elevated blood pressure, either triggers or associates with multiple cardiovascular disease processes, such as atherosclerosis,[234] cardiac hypertrophy,[235] coronary microvascular dysfunction,[236] IHD, MI, stroke, and HF,[237–240] as well as kidney disease and failure.[241] It is considered the leading cardiovascular disease to cause premature deaths.[242] While hypertension represents a prototypic multifactorial disease with multiple risk factors and varying etiologies, essential hypertension, where no specific etiology is identified, comprises 90% of cases and has been defined as a vascular pandemic due to its estimated staggering worldwide prevalence of 1.39 billion individuals.[243]

m^6A modification

Emerging evidence from human functional genome-wide association studies suggest that m^6A-related single-nucleotide polymorphisms (m^6A-SNPs) are associated with elevated blood pressure.[112] These are linked to blood mononuclear cells' expression of hypertension-associated molecules, including zinc-finger protein 589 (ZNF589), β1-adrenergic receptor, and Golgi SNAP receptor complex member 2 (GOSR2).[113] ZNF589 is a member of Krüppel-associated box domain zinc-finger family of epigenetic regulators known to maintain pluripotency in HSCs,[244] and adrenergic β1-receptor is an independent factor in predicting the treatment outcome for hypertension with β-blockers.[245] The hypertension-associated m^6A-SNP (Lys67Arg) in the *GOSR2* gene is the same as previously associated with the disease.[246] However, experimental approaches are imperative to properly evaluate whether a functional role exists for these target gene m^6A-SNPs in hypertension.

As an additional link between epitranscriptomics and hypertension, an SNP-variant of FTO has been associated with obesity and elevated systolic blood pressure.[114] The contribution of FTO to vascular tone was hypothesized to be governed by two specific hypothalamic nuclei,[247] which are known to substantially express FTO.[248] However, a more pertinent and peripheral mechanism of action for FTO in hypertension has recently been identified. Conditional endothelium-targeted knockout of FTO during continuous lipid-diet-induced obesity, vascular dysfunction, and hypertension was found to be protective against hypertensive phenotypes via a novel FTO-mediated pathway controlling myogenic tone.[117] Specifically, the loss of FTO upregulated endothelial prostaglandin D_2 (PGD_2) production via overexpression of its main synthase, lipocalin-type prostaglandin D synthase (L-PGDS), in resistance arteries, and thus alleviated specifically obesity-induced vascular dysfunction and hypertension but did not alter the baseline blood pressure.[117] It is of translational and therapeutic interest that human artery specimens from obese individuals have been reported to overexpress FTO, and its pharmacological inhibition with either rhein or FB23-2 *ex vivo* also exerted favorable increases in both prostaglandin D_2 production and myogenic tone.[117] In addition, considering that the upstream regulatory pathway responsible for the noted FTO upregulation in endothelium remains veiled, it is interesting to combine a notion that leptin, a major adipocyte-secreted systemic adipokine, has been shown to upregulate FTO in cardiomyocytes.[118] In contrast to its beneficial role in ischemic HF (discussed later), upregulated FTO has been described as detrimental in hyperlipidemia-induced cardiomyopathy.[154] Hence, investigations assessing the role of leptin possibly also regulating endothelial and cardiomyocyte FTO expression in obesity-related hypertension and cardiomyopathy might reveal an unrecognized mechanism within their development.

Last, in pericytes of spontaneously hypertensive rats, the overall m^6A methylome has been reported to be hypomethylated, which not only suggests either increased m^6A eraser or decreased writer activity but also underlines the putative role of also other vascular cell types within hypertension development from an epitranscriptomic point of view.[115] Taken together, although reports regarding m^6A in hypertension remain limited, it is evident that targeted investigations to promote our understanding of m^6A in hypertension control are needed.

A-to-I editing

Interestingly, hypoxic A-to-I editing of miR-27a-3p, which has been established to regulate endothelial *GOSR2* mRNA expression,[249] has been shown to induce a major shift on its targetome.[250] In aortas from hypertensive patients and mice, major ADAR2-mediated A-to-I editing events were identified in the vasculature in the actin crosslinking protein *Flna* mRNA.[116] *FLNA* A-to-I editing is scarce

in human fetal hearts (3%) and increases considerably in adulthood (15%).[171] Reduced *FLNA* mRNA editing, as found in human postmortem aortic-arterial samples, strongly correlated with left ventricular hypertrophy, a strong indicator of significant hypertension during life.[251] Moreover, when *Flna* mRNA was rendered uneditable by deletion of its 228-bp intronic region, transgenic mice demonstrated increased perivascular fibrosis, diastolic blood pressure, and left ventricular hypertrophy that finally progressed to cardiac dysfunction.[116] In hemizygotic *Flna*$^{0/+}$ mice, vascular smooth muscle cell (VSMC)-restricted and tamoxifen-induced *Flna* knockout (*smFlna*$^{0/-}$) led to a drop in basal blood pressure due to impaired calcium influx and mechanotransduction.[252] However, global ADAR2 knockout mice have been reported to have unaltered blood pressure profiles.[162]

Pulmonary hypertension

Hypertension of the pulmonary circulation is estimated to affect 1% of the global population.[253] Increased pulmonary pressure greatly increases the workload of the right ventricle, which is prone to develop irreversible dilatation and failure, *cor pulmonale*, which is associated with up to 60% mortality when acute and unstable.[254] Histopathologically, pulmonary hypertension is hallmarked by overt proliferation of VSMCs with consequent muscularization of the pulmonary artery walls. Current drugs fall short in both tackling its underlying pathophysiology and managing its disabling symptoms.[255]

m^{6}A modification

While m^{6}A has emerged as a regulator and potential therapeutic target in pulmonary hypertension,[81] to the best of our knowledge—excluding an indirectly-relevant report observing ADAR1 to promote VSMCs proliferation and neointima formation[256]—no reports currently exist describing A-to-I editing in pulmonary hypertension. In a hypoxic pulmonary hypertension rat model, Su et al. identified several m^{6}A-modified circular RNAs (circRNAs) affecting circRNA–miRNA–mRNA interactions.[74] Hyperproliferation of rat pulmonary artery smooth muscle cells (PASMCs) was associated with increased METTL3 expression. Increased m^{6}A on phosphatase and tensin homolog (*Pten*) mRNA led to reduced PTEN expression in a YTHDF2-dependent manner involving the phosphoinositide 3-kinase–AKT serine/threonine kinase 1 (PI3K–Akt) pathway.[75] Moreover, YTHDF1 is upregulated alongside increased m^{6}A contents in hypertensive pulmonary arteries, which were shown to promote hyperproliferation of human PASMCs *in vitro* as well as pulmonary hypertension development *in vivo* by increasing m^{6}A-dependently translation of melanoma antigen gene (MAGE) family member D1 (*MAGED1*) mRNA, expression of subsequent protein, which finally led to upregulation of proliferating cell nuclear antigen (PCNA).[80] Knockdown of METTL3 abrogated all these effects.[80] Interestingly, a recent report suggests WTAP to promote PASMC ferroptosis,[79] a recently discovered morphologically (mitochondrial diminution), biochemically (iron-dependent reactive oxygen species [ROS] production), and genetically (independent of proapoptotic genes) distinct form of programmed cell death that, as recently reviewed, has been found to operate in many CVDs.[257] Specifically, WTAP was pinpointed as proferroptotic in PASMCs via m^{6}A-dependent enhanced translation of glutathione peroxidase 4 (*Gpx4*) mRNA and subsequent GPX4 expression.[79] Further, administration of an ferroptosis inhibitor, ferrostatin-1, ameliorated pulmonary hypertension *in vivo*.[79] Moreover, WTAP has also been implicated in VSMCs hyperproliferation, a key process in pulmonary hypertension by promoting artery wall muscularization. Namely, Panax notoginseng saponin was shown to inhibit VSMC hyperproliferation via upregulating WTAP and m^{6}A.[258]

Histopathologically, while the pulmonary artery-isolated rat PASMCs upregulate METTL3 writer in hypoxia, and the m^{6}A erasers FTO and ALKBH5 are downregulated, m^{6}A writer complex subunits METTL14 and WTAP remain unaltered.[75] On the other hand, no alterations at a level of mRNA in either *Mettl4*, *Wtap*, *Fto*, or *Alkbh5* were observed in hypoxic and hypertensive rat pulmonary arteries *in vivo*.[75] Upregulation of METTL13 and YTHDF1, and downregulation of FTO and ALKBH5, have been reported in both murine and human adult hypertension-tormented pulmonary arteries and parenchyma.[78,80] No changes were observed in the expression of the majority of other m^{6}A regulators, including METTL14, VIRMA, RBM15, YTHDF2-3, YTHDC1-2, or IGF2BP1-3.[78] Contrary to the above findings, lung tissue of rat pups with hypoxic pulmonary hypertension showed downregulated levels of m^{6}A residues in RNAs, and decreased expression of METTL3, METTL14, FTO, and ALKBH5.[77]

Together, the above differences delineate age-, tissue-, and cell-specific alterations of m^{6}A regulators in pulmonary hypertension, thus highlighting the need for more cell-type-specific investigations. These investigations could include pulmonary endothelial cells (which seem to be enriched with YTHDF1 in human idiopathic pulmonary hypertension and fibrosis[80]), fibroblasts, resident leukocytes, and pulmonary cells of the lung parenchyma. A recent study by Zhou et al. elegantly unveiled a cell-specific function for an epigenetic regulator SET domain containing 2, histone lysine methyltransferase (SETD2), in hypoxic pulmonary hypertension with its VSMC-targeted knockout as a pulmonary hypertension-promoting regulator and positive upstream regulator of METTL14 *in vivo*.[76]

Altogether, as the m^{6}A erasers are consistently downregulated within various pulmonary hypertension tissue specimens, and METTL3 knockdown effectively abrogates pathology development,[80] reducing overall m^{6}A content could constitute an avenue for therapeutic benefit.

Cardiac hypertrophy and failure

Adult differentiated cardiomyocytes react to increased workload by increasing their size and the number of sarcomeres for better contractility.[251] Over time with, for example, increased ischemic myocardial damage and loss of cells, such hypertrophic compensation for the reduced functionality eventually fails. Decompensated hypertrophy is characterized by interstitial fibrosis, cardiomyocyte apoptosis, inadequate angiogenesis, increased ROS production, mitochondrial dysfunction, and activation of fetal gene expression programmes.[251,259] This perilous sequence of events often culminates

in HF.[260,261] The ensuing cardiac dysfunction is often divided into HF with either reduced (systolic dysfunction) or preserved (diastolic dysfunction) ejection fraction (HFrEF and HFpEF, respectively).

m^{6}A modification

A rapidly accumulating and prominent body of evidence indicates that epitranscriptomics, and especially m^{6}A, influence not only the initiation of hypertrophy but also progression toward dysfunction and ultimately HF.[118–123,131]

Cardiac hypertrophy. While transcript hypomethylation has been shown to predominate in pressure-overloaded hypertrophic murine hearts, the number of transcripts with overall changes in m^{6}A modifications measures greater than the amount of differentially expressed transcripts, suggesting notable functional role for m^{6}A regulating machinery in hypertrophy.[122] However, the functional role of METTL3 writer in hypertrophy does not appear to be straightforward. An initial study by Kmietczyk et al. revealed that at the transitory point from the acute phase of adaptation to the early hypertrophic remodeling two days after pressure-overload induction, the expression of METTL3 and the m^{6}A content of several hundred mRNAs were downregulated.[121] In concert, when METTL3 was overexpressed, the hypertrophic response to pressure overload was attenuated.[121] However, pressure-overload-induced hypertrophy has also been associated with increased cardiomyocyte total RNA m^{6}A content *in vitro*. Here, METTL3 overexpression *in vivo*, with a different mouse strain and expression method, was demonstrated to act as a spontaneous activator of hypertrophy with no external triggers, but not to affect the hypertrophic adaptation in response to pressure overload.[120] Interestingly, spontaneous hypertrophy also occurs in skeletal muscles following METTL3 overexpression suggesting conserved mechanisms.[262] As the obvious cause(s) responsible for the noted discrepancy within the myocardium remain unknown, validation of the findings with parallel identification of various operant downstream mechanisms can be expected to ultimately shed light on the matter.

The identified molecular mechanisms involving METTL3 in hypertrophy are complex. First, Gao et al. identified and named a novel piwi-interacting RNA, greatly overexpressed in response to cardiac hypertrophy, as cardiac-hypertrophy-associated piwi-interacting RNA (CHAPIR) and reported it to suppress METTL3 expression to ultimately promote development of pathological hypertrophy.[123] Hence, METTL3-mediated m^{6}A methylation was proposed to be cardioprotective against pathologic growth. In finer detail, pressure-overload-induced hypertrophy was associated with increased complexing of CHAPIR with piwi-like RNA-mediated gene silencing 4 (PIWIL4), which subsequently suppressed METTL3 via direct binding, decreased poly(ADP-ribose) polymerase family member 10 (*Parp10*) mRNA m^{6}A methylation, and consequently upregulated PARP10 protein via relieved YTHDF2-dependent degradation. Further downstream, increased PARP10 promoted mono-ADP-ribosylation of glycogen synthase kinase-3 β (GSK3β), which ultimately resulted in nuclear accumulation of the transcription factor nuclear factor of activated T cells 4 (NFATC4) and transcription induction of hypertrophy-related genes.[123] However, in another experimental study, with yet another stimulus for murine hypertrophy induction via long-lasting subcutaneous infusion of angiotensin II (AngII), Lu et al. pinpointed METTL3 as a potent prohypertrophic downstream player.[126] The authors showed that the deubiquitinating enzyme ubiquitin-specific peptidase 12 (USP12) is upregulated in hypertrophy, stabilizes E1A-binding protein p300 (p300), and enables it to upregulate METTL3.[126] Furthermore, as insights from skeletal muscles also suggest METTL3 to drive spontaneous hypertrophy via an m^{6}A-induced YTHDF2-dependent degradation of activin A receptor type 2A (*Acvr2a*) mRNA, consequently blocking a muscle-conserved anti-hypertrophic myostatin (an ACVR2A ligand) signaling pathway, it is tempting to speculate that such epitranscriptomic control also operates within myocardium.[262] Indeed, ACVR2A inhibition appears therapeutic after MI by (1) promoting early-stage compensatory hypertrophy (concentric hypertrophy) via activated Akt signaling, (2) reducing myocardial fibrosis, and (3) inhibiting dilative late-stage pathologic cardiac remodeling (eccentric hypertrophy).[263]

Aiming to delve further into the methodological differences to pinpoint putative mechanisms for the observed discrepant roles of METTL3 in hypertrophy, the prohypertrophic association for METTL3[120,126] arises from studies that used robustly cardiomyocyte-targeted overexpression methods and distinct murine strains from those observing beneficial effects, including opposed pathological hypertrophy, preserved contractility, and post-MI angiogenesis.[56,121,123,140] Additional studies addressing the cell-type-specific nature of the findings in various myocardial cell lineages in hypertrophy are warranted.

In addition, the direct downstream effectors of m^{6}A, the m^{6}A readers, are important as their altered regulation might considerably affect the way METTL3-mediated m^{6}A modification is interpreted by the cells. Albeit recently challenged,[264] the major scheme of YTHDF m^{6}A reader family functions denote YTHDF2 as a repressor of m^{6}A-methylated mRNAs, YTHDF1 a stabilizer of m^{6}A-bound transcripts, and YTHDF3 to act in both directions.[38] Such divergent functions provide a functional basis for a conjecture that these readers might be differently regulated within different hypertrophy models, and underly the observed distinct phenotypes. As an indirect support for such speculation are notions that the YTHDF2-dependent *Parp10* mRNA degradation appears protective from pathological hypertrophy,[123] and YTHDF2 has also been denoted with similar protective role in mice and specified in mice primary cardiomyocytes to operate via m^{6}A-dependent *Myh7* mRNA decay.[125] In contrast, YTHDF2 was recently revealed to promote rat cardiomyocyte hypertrophy with lncRNA MIAT (MI-associated transcript) acting as a direct positive upstream regulator of YTHDF2-mediated m^{6}A-dependent degradation of carnitine palmitoyltransferase 1A (*Cpt-1a*) mRNA downregulating subsequently CPT-1a protein, a rate-limiting enzyme in mitochondrial fatty acid oxidation related to PPARα signaling.[129] Such divergent functions for a single YTHDF paralog during qualitatively varied modeling species and conditions add an another layer of regulation to

be considered. Furthermore, as upregulation of both *Ythdf2* and YTHDF2 in 0.2μM AngII-treated rat cardiomyocytes seems to wane with more potent 1 μM AngII induction, the quantitative aspects also warrant standardization.[129]

No targeted genetic interventions against either YTHDF1 or YTHDF3 in hypertrophy have yet been reported, not to mention the almost totally veiled role of the litany of other established m^{6}A readers (Figure 1). Indeed, the antihypertrophic effects of miR-133a, targeting effectively m^{6}A-methylated RNAs via its complementary m^{6}A-motif in its seed sequence, has been reported to depend on IGF2BP2 complexing with the m^{6}A-methylated target transcripts.[124] As IGF2BP2 is a major myocardial paralog of the IGF2BP m^{6}A reader family[233] with established upstream regulators lncRNA Airn (antisense of IGF2R non-protein coding RNA) in cardiomyocytes[265] and high-mobility group AT-hook 2 (HMGA2) protein in skeletal myoblasts[266] controlling migration, apoptosis, and proliferation of these cells, targeted investigations toward this m^{6}A reader may also yield some clarification. Last, the role of cardiac hypertrophy-promoting mitogen-activated protein kinase/extracellular regulated MAP kinase (MAPK/ERK) pathway[267] also warrants attention, as it was recently shown to positively regulate m^{6}A methylation through phosphorylation-dependent stabilization of the METTL3 writer complex.[268] Maslinic acid, a pentacyclic triterpenoid known to inhibit the ERK pathway activation, has recently been unveiled to protect against pressure-overload cardiac hypertrophy via an as-yet unclear mechanism of METTL3 downregulation.[128]

FTO m^{6}A eraser has also been observed with contrasting but tissue- and cell-type specific functions in hypertrophy. The first report assessing FTO in hypertrophy by Gan et al. pinpointed upregulated FTO in hypertrophic cardiomyocytes treated with leptin, a pro-satiety and prohypertrophic adipokine,[269] through JAK–STAT3–cut-like homeobox 1 pathway p110 isoform (CUX1p110).[118] Here, FTO silencing unveiled its prohypertrophic function *in vitro*,[118] a finding later recapitulated with phenylephrine treatment,[121] albeit the FTO-regulated downstream mRNAs responsible for the phenotype in these cell cultures remained veiled. As an interesting link, JAK–STAT3 signaling has been implicated in cardiac anti-apoptosis, cell-cycle re-entry, differentiation, regeneration, fibrosis, hypertrophy, MI, HF,[270,271] and in the regulation of induced pluripotency by acting through m^{6}A-YTHDF1/YTHDF2 and suppressor of cytokine signaling 3 (SOCS3).[272] Congruent with these prohypertrophic findings, Tanshinone IIA (TanIIA), an active compound from *Salvia miltiorrhiza*, was shown to inhibit pressure-overload-induced myocardial hypertrophy, the mechanism, as evaluated in AngII-stressed cardiomyocyte culture, of which was suggested to operate via downregulation of ALKBH5 to downregulate Galectin-3 via respective mRNA m^{6}A methylation.[141]

Contrasting results have also been obtained, however. While FTO knockout in a model of pressure-overload-induced HFrEF decreases contractility and increases ventricular dilatation,[121] its overexpression in a model of diabetic cardiomyopathy has been shown to inhibit fibrosis and hypertrophy.[127] On the other hand, global knockout of FTO, unlike the above cardiomyocyte-targeted interventions, has been reported to result in promoted hypertrophy.[119] This finding receives weak support from a positive correlation observed among a small case series of patients with congenital FTO deficiency and hypertrophic cardiomyopathy.[273] Much like that for METTL3 m^{6}A writer, the contrasting findings regarding m^{6}A erasers may be explained by the diversity of the models and hypertrophic stimuli used, as these will yield distinct transcriptomes available for modification. Further, the expressed m^{6}A reader profiles, concurrent with availability of needed functional subunits, cofactors, or substrates, all may affect how the m^{6}A is interpreted by the cells. The use of standardized methodologies with broader concurrent consideration of m^{6}A readers may help to crystallize this rapidly developing field.

Last, to identify conserved epitranscriptomic pathways in hypertrophy, Hinger et al. utilized a rat-to-human cross-species comparison approach from myocardium samples of human non-ischemic hypertrophy against that of isolated rat hypertrophied cardiomyocytes. Intriguingly, they found a set of 38 mRNAs with conserved m^{6}A enrichment.[131] Of these, five contained conserved m^{6}A sequence loci, and only repressor element silencing transcription factor 1 (*Rest1*) and splicing factor 3b subunit 4 (*Sf3b4*) mRNAs were modified at their CDS. Moreover, the baseline comparison of non-hypertrophic human myocardium against rat cardiomyocytes revealed 11 m^{6}A-enriched transcripts, of which only *coronin 6*, a transcript encoding an actin filament-binding protein,[274] emerged as a conserved m^{6}A-modified transcript at a specific sequence locus within its 3′ UTR,[131] a known critical RNA regulatory hub.[232] Intriguingly, while the function of coronin 6 has not yet been studied in the heart, its protein levels were shown to correlate with those of METTL3 and to be downregulated in hypertrophic cardiomyocytes.[131]

Ischemic and hypertrophic cardiomyopathy. Akin to hypertrophy, the roles of post-transcriptional regulation in hypertrophy in both murine HF models and human ischemic HF and DCM specimens are highlighted as the number of differentially m^{6}A-methylated mRNA transcripts seem to outweigh up to 5- to 7-fold the differentially expressed genes.[121,122] Further, mice-to-human cross-species-conserved m^{6}A-altered transcripts in HF models have been associated with regulation of calcium fluxes, cardiac contraction, and VSMC differentiation.[122]

Experimental studies targeting FTO expression suggest it to be cardioprotective against development of HF and fibrosis.[107,122,127] While FTO expression has been described as either repressed[107,116,131] or unaltered[121,122] in HFrEF, it has been reported to be upregulated in HFpEF.[151] Based on a combination of measurements from hypoxic cardiomyocytes, ischemic myocardium, and clinical HFrEF samples, such activity has been suggested, at least partially, to relate to demethylation of sarcoplasmic/ER Ca^{2+}ATPase 2a (*Serca2a*) mRNA m^{6}A, resulting in increases in the amount of SERCA2A protein and improved Ca^{2+} signaling.[107] Such findings link the m^{6}A-mediated

regulation of mRNA translation and respective protein production to cardiomyocyte contraction kinetics and more generally with Ca^{2+} dynamics in HF.[107] According to lessons from neurons, FTO can also demethylate Ca^{2+}/calmodulin-dependent protein kinase II (*CaMKII*) mRNA, a key mediator of cardiomyocyte Ca^{2+}-dependent contraction,[275] to increase its expression.[276] In addition, decreased m^{6}A methylation of both mouse and human *Calmodulin 1* mRNAs (a core member of the CaMKII pathway) lead to its reduced protein expression in the failing myocardium.[122] On the other hand, hypermethylation of the high-conductance intracellular calcium channel ryanodine receptor 2 (*Ryr2*) and *RYR2* mRNAs has been observed in mice post MI and human ischemic HFrEF myocardial specimens, respectively. These modifications may thus also contribute to disturbances in intracellular calcium signaling during ischemia and proneness for arrythmias, which is ameliorated with FTO overexpression in hypoxic cardiomyocytes *in vitro*.[107] Finally, FTO has recently been proposed to antagonize the development of pressure-overload cardiac dysfunction via duplex mechanism converging to promote glycolysis.[175] Namely, FTO was shown to upregulate phosphoglycerate mutase 2 (PGAM2) in cardiomyocytes, a key enzyme in glycolysis, via m^{6}A hypomethylation of *Pgam2* mRNA, and promote AKT phosphorylation, which led to enhanced insulin-responsive glucose transporter type 4 (*Glut4*) gene transcription, GLUT4 expression, and glucose intake.[175]

Despite varying ALKBH5 expressions in HFrEF,[107,121,122,131,151] its overexpression has also been shown to be cardioprotective against the development of ischemic HF.[54] Taken together with the above notions also for FTO, akin to cardiac regeneration, upregulation of FTO and ALKBH5 emerges as a putative therapeutic handle to antagonize HF development and progression. However, mechanistic insights remain limited.

METTL3 levels have been observed to be repressed in both experimental HFpEF[151] and pressure-overload hypertrophic HFrEF,[122] but overexpressed[131] or unaltered in clinical samples of ischemic HF[107] or DCM.[121,122] In preclinical models, knockdown of METTL3 has been shown to reduce fibrosis,[60,121] preserve cardiac function,[60,140] and enhance both autophagy[134] and regeneration-associated markers.[140] In concert, METTL3 overexpression has been shown to drive progressive eccentric remodeling, ventricular ballooning, and ultimately systolic dysfunction.[120] Hence, the observed downregulation of METTL3 in murine HF models may act as an active, but insufficient, compensation mechanism. However, the measured both unaltered and upregulated METTL3 in many small sets of human HF samples[107,121,122,131] highlight the need to keep in mind the probable species-specific differences.

Dominant hypomethylation of the m^{6}A-methylomes in both experimental and human HFrEF have been reported.[122] At the same time, the still-m^{6}A-enriched transcripts were positively correlated with polysome occupancy and enhanced translation, an interesting finding not recapitulated in the baseline myocardium.[122] Hence, it can be speculated that the downstream m^{6}A reader milieu undergoes notable reorganization within the failing myocardium with as-yet veiled functional consequences.

To date, only the YTHDF2 m^{6}A readers have had their protein expression evaluated in failing myocardium in a targeted fashion. Namely, while human failing dilative cardiomyopathy samples upregulate YTHDF2 protein,[125] the *Ythdf2*, alongside *Ythdf1*, *Ythdf3*, and *Ythdc1*, mRNA levels have been measured unaltered in experimental models of HFrEF and human DCM.[122,151] Moreover, YTHDF2 overexpression has been shown to be cardioprotective in pressure-overloaded failing myocardium.[125] As such, the dominating hypomethylation in failing murine and human myocardium[122] may be a consequence of an active compensation mechanism where aberrantly m^{6}A-modified transcripts are degraded by YTHDF2 to enable effective positive selection of a smaller subset of cardioprotective m^{6}A-methylated mRNAs for recruitment to polysomes and enhanced translation by other m^{6}A readers, such as YTHDF1.

Multiple mechanisms, most probably in a synergistic fashion, tend to promote m^{6}A in mRNAs in failing myocardium. Indeed, as discussed later in future perspectives, hypoxic metabolism in general may hamper m^{6}A eraser function, but FTO and ALKBH5 eraser levels have also been measured to downregulate in ischemic myocardium,[107,131,135] and their overexpression—as well as METTL3 knockout—has proved beneficial against the development of HF.[140] Furthermore, the relationship between YTHDF1 protein and*Ythdf1* mRNA levels might be complex within the failing myocardium.[122] For example, the post-MI cardioprotection of ALKBH5 against HF development seem to be conveyed by hypomethylation-dependent stabilization of *Ythdf1* mRNA, thus upregulating YTHDF1 protein without altering its transcription.[54] Moreover, recent evidence suggests most m^{6}A to be non-functional enzymatic noise, also in myocardium.[277] An *in vivo* HF model with YTHDF1 overexpression, in conjunction with YTHDF2 knockout, and vice versa, come with power to address such speculations.

Despite myocardial YTHDF3-targeted experimental studies remaining to be published, an intersection with HF exists, as bioinformatic reanalysis of published protein expression datasets has revealed YTHDF3 to be downregulated in human ischemic failing myocardium.[133] Interestingly, YTHDF3 seems to promote translation of m^{6}A transcripts common also for YTHDF1 via recruitment to polysomes, but to also perform a contrasting role for other transcripts.[278] Intriguingly, YTHDF3 has thus been suggested as a modulatory pivot for the effects of YTHDF1 and other m^{6}A binders.[278,279] Furthermore, YTHDF3 has been suggested to suppress YTHDF1 in ESC-derived differentiating cardiomyocytes *in vitro* with an as-yet veiled mechanism.[160] Finally, lessons from the fruit fly suggest that its single YTHDF orthologue binds Fmr1, an orthologue of the mammalian m^{6}A reader FMRP, and consequently inhibit its translation.[280] FMRP also associates to polysomes and negatively regulates bound transcript translation.[281,282] As FMRP has protective effects against inflammatory cardiomyocyte injury[283] and counteracts myocardial mitochondrial proton leak,[284] as well as regulating several key

processes against development of cardiac dysfunction,[285,286] namely RNA splicing and export,[287] FMRP, as a relatively unexplored m^{6}A reader, should be investigated in the failing heart.

Dilated cardiomyopathy. Various causes ranging from toxins and infections to hereditary mutations can disrupt myocardial architecture and develop a pathophenotype of DCM, which is hallmarked by outward enlarged and thin-walled, often poorly contracting, and ultimately failing ventricles.[288] While the myocardial m^{6}A content in clinical DCM samples has been reported to be increased, the expression levels of the major writers and the FTO eraser remain unaltered.[121] However, yet another m^{6}A reader, YTHDC1, has been assigned a key cardioprotective role against DCM development by controlling alternative splicing in mice.[150] Indeed, expression of Titin, a giant myofilament protein that serves as a molecular spring during cardiomyocyte contractions and encoded by a colossal 364 exon-containing *Titin* gene, was revealed to rely on the m^{6}A reader YTHDC1 for the proper splicing of its m^{6}A modified pre-mRNA.[150] While the m^{6}A-dependent and YTHDC1-guided *Titin* pre-mRNA splicing produced a shorter and more rigid Titin isoform, N2B, cardiomyocyte-targeted conditional YTHDC1 knockout led to expression dominance of longer and less stiff N2BA isoform manifesting with DCM phenotype and ultimately HF.[150] Considering that *Titin* gene mutations, which disrupt its proper maturation, underly nearly every fourth case of congenital DCM when the causative mutation can be identified,[289] these findings appear to be of potential therapeutic interest. The N2BA isoform has also been reported to increase at the expense of the stiffer N2B isoform in human end-stage DCM.[290] In sum, this discovery warrants evaluation of YTHDC1's role in human *Titin* pre-mRNA maturation and pathogenesis of DCM, which is often considered idiopathic.[289] As YTHDC1 remains currently the only known helicase-domain-containing m^{6}A reader,[38] the above findings may also prove to be a catalyst to broaden the epitranscriptomic considerations in CVDs toward RNA splicing control.

Metabolic cardiomyopathy. Most metabolic pandemics of our time, including obesity, hyperlipidemia, and type 2 diabetes, are increasingly being linked with both m^{6}A and to its role in the heart. A mechanistic summary of these emerging molecular findings is presented in Figure 5. Interestingly, and in sharp contrast with ischemic HF, FTO inhibition appears to be therapeutic in hyperlipidemia- and palmitic acid (PA)-induced cardiomyopathy and cardiomyocyte inflammation, respectively, where its targeted pharmacological inhibition by a LuHui monomer derivative was reported to provide therapeutic benefit, likely via disrupted mRNA translation of cluster of differentiation 36 (*CD36*), alias scavenger receptor class B protein (*SR-B2*).[154] While METTL3 and ALKBH5 have been reported to be downregulated in PA-induced inflammation in human cardiomyocytes,[154] METTL3 was measured to be upregulated in mice myocardium with high-fat-diet-induced cardiomyopathy,[155] again highlighting methodological, cell-type-specific, and species-dependent differences.

While METTL14 appeared downregulated in a mouse model of diabetic cardiomyopathy,[152] ALKBH5 was upregulated and, contrary to its function in ischemia,[134] promoted cardiomyocyte apoptosis ultimately via YAP1 inactivation.[153] Mechanistically, in high-glucose-treated cardiomyocytes, ALKBH5 was unveiled to demethylate forkhead box O3 (*Foxo3*) mRNA m^{6}A, upregulate the protein, and activate the transcription of circular RNA cerebellar degeneration-related protein 1 antisense RNA (circ-CDR1as), which enabled blockage of ubiquitination of mammalian sterile 20-like kinase 1 (MST1). This induced large tumor suppressor kinases 1/2 (LATS1/2), which ultimately inactivated YAP1 via phosphorylation.[153] This finding contrasts the results from ischemic regenerating[54] and reperfused[145] myocardium where ALKBH5 and FTO, respectively, were found to upregulate YAP1 via other mechanisms. Similarly, increasing m^{6}A by METTL14 overexpression appeared therapeutic through suppression of nucleotide-binding oligomerization domain-like receptor (NLR) family pyrin domain containing 3 (NLRP3)-mediated cardiomyocyte pyroptosis, which was firmly linked with a m^{6}A-dependent and YTHDF2-mediated degradation of lncRNA terminal differentiation-induced non-coding RNA (TINCR).[152]

As cardiac insulin signaling converges in translocation of GLUT4 receptor to the cardiomyocyte plasma membrane, and its disturbance is a key etiologic factor in diabetic cardiomyopathy,[291] it is worthwhile to reiterate here the notion that FTO protects the murine heart from pressure-overload-induced dysfunction via Akt-mediated GLUT4 upregulation.[175] Hence, the therapeutic role of FTO in diabetic cardiomyopathy, perhaps via regulation of GLUT4 expression, warrants targeted attention. In sum, although metabolic disease causes alterations in the myocardial m^{6}A epitranscriptomic landscape and its regulatory networks, further specific characterizations are required to unleash the therapeutic and biomarker potential of the epitranscriptomic modifications.

A-to-I editing

Cardiac hypertrophy. Reports directly investigating A-to-I editing in hypertrophy remain scarce. While ADAR1 protein levels have been reported to promptly decrease following murine induction of pressure overload and hypertrophy, the *Adar1* mRNA levels remain unaltered until the decompensated phase of hypertrophy with HF.[130] Moreover, conditional cardiomyocyte-specific ADAR1 knockout results in hypertrophy and interstitial fibrosis.[130] On the other hand, after ADAR2 knockout, Altaf et al. identified myocardial downregulation of the let-7 miRNA family,[132] known regulators of cardiac hypertrophy,[292] as well as reduced levels of hypertrophy and fibrosis-associated miR-29b.[293–295] However, ADAR2 has also been reported to be unrelated to the size regulation of unstressed cardiomyocytes when either silenced or overexpressed *in vitro*. While ADAR2 is reported to upregulate in milder exercise-induced physiological hypertrophy *in vivo*, the consequent functional assessments corroborated the findings from the cell culture.[146]

Last, miR-1, an abundant and hypertrophy-limiting miRNA in the heart,[296–299] has been shown to act as an ADAR repressor.[300-301] As oxidative epitranscriptomic modification of miR-1 at its seed

sequence position 7 guanosine (7o^{8}G-miR-1) changes miR-1 function and provides it with prohypertrophic properties,[302] further studies are required to discover links between A-to-I editing and miRNAs in cardiac hypertrophy. Notably, such functions for miRNA editing have been demonstrated for angiogenesis, as described later in a dedicated section for angiogenesis.

Heart failure. A-to-I editing appears to be critical in HF pathophysiology as forced cardiomyocyte ADAR1 knockout during pressure overload accelerated cardiac dysfunction and adverse dilatation, and resulted in massive lethality in an UPR-dependent manner.[130] Furthermore, and speculating an underlying mechanism, as the above seems consistent with the ADAR1's role to keep innate immune response within developing murine myocardium at bay via MDA5–MAVS–INF–(ER stress)–UPR pathway inhibition (described above in section "cardiogenesis and cardiac regeneration"), it appears interesting that case reports have described several unfortunate children with either congenital *MDA5* gain-of-function[303] or *ADAR1* loss-of-function mutations,[156] both causing a class I interferonopathy, to develop severe cardiac valve calcifications, HF, and ultimately increased premature lethality.

Moreover, it is intriguing to consider the established role of A-to-I editing in control of angiogenesis—a key process in HF[190,304,305]—via editing of miRNAs[65–67] jointly with miR-1, which has been implicated in cardiogenesis,[296] hypertrophy,[297–299,306] cardioprotection,[307] and atherosclerosis,[308] as it represses ADAR1 non-cardiac tissues[300,301] and acts oppositely in favor of hypertrophy when modified by oxidation.[302] Moreover, ADAR1 knockout downregulated multiple miRNAs within failing myocardium.[130] Hence, evaluation of an ADAR1-mediated A-to-I editing in controlling, perhaps via miRNA editing, angiogenetic responses in myocardium appear an avenue, when better understood, that could ultimately provide feasible molecular targets to ignite therapeutic revascularization to repair the failing heart.

Atherosclerosis

Atherosclerosis, described as the fattening and hardening of arteries, is promoted by such factors as aging, hypertension, obesity, smoking, and renal failure.[19,309,310] Mechanistically, endothelial shear stress, dysfunction, and inflammation drive atherosclerosis, the pathophysiology of which is characterized by subendothelial deposition of lipids—especially low-density lipoproteins (LDLs)—to arterial walls. The ensuing endothelial dysfunction and increased permeability promote adherence and translocation of immune and inflammatory cells that engulf the deposited lipids and turn into foam cells.[311] The plaques calcify and grow over time, may rupture, and eventually obstruct blood flow causing tissue ischemia and MI when occurring in a coronary artery supplying the myocardium. If the patient survives, such infarction is a major risk factor for malignant arrythmias as well as further infarctions and development of HF in the future.

Several molecular mechanisms in atherosclerosis and involving m^{6}A and A-to-I editing have been discovered recently. A summary of these molecular pathways is presented in Figure 3, and a detailed overview involving these modifications with respect to atherosclerosis pathophysiology is presented in Figure 6. In addition, Figure 7 summarizes currently discovered molecular interactions involving m^{6}A during macrophage inflammation and formation of foam cells.

m^{6}A modification

Disturbed flow, endothelial dysfunction. The endothelial stress response is considered as the initial step in the pathogenesis toward clinically manifest atherosclerosis.[312] Notably, METTL3-mediated m^{6}A RNA deposition has been suggested to have a key role in early atherosclerosis. Namely, oscillatory blood flow was found to increase METTL3 expression in endothelial cells predisposed to oscillatory flow and stress.[92] The authors identified METTL3 to function as an upstream activator of nuclear factor kappa B (NF-κB), an enhancer of NLR family pyrin domain containing 1 (NLRP1) protein and repressor of Krüppel-like factor 4 (KLF4). They demonstrated *NLRP1* mRNA stabilization (upregulating NLRP1) to occur via m^{6}A-YTHDF1 and *KLF4* mRNA degradation (downregulating KLF4) to be dependent on m^{6}A-YTHDF2 interactions. NF-κB is an inflammatory master regulator and NLRP1 is crucial for inflammasome activation in endotheliocytes during atherosclerosis,[313] while KLF4 is a key regulator of vascular homeostasis and endotheliocyte function.[314] However, a contrasting effect for oscillatory-flow-induced endothelial dysfunction has also been described. With a considerably shorter period of oscillatory stimulus, rather than upregulation, Li et al. described endothelial METTL3 downregulation with consequent epidermal growth factor receptor (*EGFR*) mRNA m^{6}A-hypomethylation-mediated EGFR upregulation, which was linked with promoted atherosclerosis both *in vitro* and *in vivo*.[95] As advocated further later in section "future perspectives", these drastically contrasting effects for METTL3 require methodologically standardized validations and further profiling of the m^{6}A reader expressions and interactions.

Endothelial and monocyte inflammation, manifest atherosclerosis. Increased expression of both METTL3 and METTL14 in endothelial cells activated by tumor necrosis factor alpha (TNF-α) have been associated with enhanced monocyte adherence and enhanced vascular cell adhesion molecule 1 (VCAM-1) and intercellular adhesion molecule 1 (ICAM-1) expression.[90] Binding to METTL14 activates the forkhead box O1 (FOXO1) transcription factor, leading to increased transcription of *VCAM-1* and *ICAM-1* mRNAs, also independently of m^{6}A.[90] METTL3/14-dependent m^{6}A methylation of *FOXO1* mRNA and subsequent binding by YTHDF1 further increase FOXO1 protein expression. Chen et al. linked the increased METTL14 expression with m^{6}A-dependent degradation of vasculoprotective *Klotho* mRNA[315] in dysfunctional endothelium.[101] Endothelial cells of human cerebrovascular plaques harbor increased METTL14 expression.[91] Zhang et al. described a METTL14-DGCR8-m^{6}A-miR-19a axis where METTL14 directly binds DGCR8 and enhances its interaction with pri-miR-19a. The resulting m^{6}A-dependent maturation of miR-19a then drives proliferation and the invasive capacities of atherosclerotic endothelial cells.[91]

Interestingly, knockdown of cardiomyocyte-secreted miR-19a improves angiogenesis after MI via hypoxia-inducible factor 1α (HIF-1α) *in vivo*.[316] Hence, it can be speculated that a m^{6}A-dependent miR-19a maturation regulates, via HIF-1α, endothelial function within myocardium.[317]

In addition to the endothelial inflammation and dysfunction, activation of the blood monocytes—especially their pro-inflammatory phenoconversion to endothelium- and plaque-penetrating atherosclerosis-promoting macrophages—is a critical atherosclerosis-promoting event recently shown to be influenced by the m^{6}A methylation (Figure 7). Increased inflammatory activation of oxidized LDL (oxLDL)-stimulated monocytes was associated with METTL3-dependent hypermethylation and YTHDF2-dependent mRNA degradation of peroxisome proliferator-activated receptor gamma co-activator 1α (PGC-1α) protein, a mitochondrial biogenesis-regulating cofactor.[100] While pro-inflammatory ROS production was increased, the production of ATP and oxygen consumption decreased alongside downregulation of electron transport chain proteins cytochrome *c* (CYCS) and NADH:ubiquinone oxidoreductase subunit C2 (NDUFC2).[100] In line with the effect on m^{6}A abundance and the adverse METTL3 upregulation, FTO has recently been assigned an anti-atherosclerotic role via inhibiting foam cell formation by controlling cholesterol efflux transporters and scavenger receptors, and suppressing both mature interleukin-1β (IL-1β) synthesis and secretion.[94] The suggested atheroprotective role of FTO was further shown *in vivo*, but the favorable effect interestingly occurred only in male mice.[94]

However, in contrast to its role in atherosclerotic endothelium, METTL14 has been associated with anti-atherosclerotic activity. Namely, a bioactive metabolite mono-(2-ethylhexyl) phthalate (MEHP) has been shown to inhibit both mRNA m^{6}A content and METTL14 expression to promote intracellular cholesterol accumulation and foam cell formation *in vitro* through disrupted cholesterol efflux accountable for an m^{6}A-dependent scavenger receptor class B member 1 (*Sr-b1*) mRNA downregulation with consequent downregulation of SR-B1 protein.[318] Furthermore, Gong et al. recently promoted the putative therapeutic importance of METTL14-mediated modification of miR-654 in human atherosclerosis via regulating cholesterol efflux.[93] Specifically, they pointed out a putative pathway consisting of METTL14–m^{6}A-(miR-654-3p)–lncRNA ZNFX1 antisense RNA 1 (ZFAS1)–ADAM metallopeptidase domain 10/RAB22A, member RAS oncogene family (ADAM10/RAB22A). In brief, such a hypothesis stems from a notion that lncRNA ZFAS1, dependent on miR-654-3p, promotes inflammation and diminishes cholesterol efflux from the atherosclerotic plaques by regulating ADAM10/RAB22A.[319] Moreover, *ZFAS1* is overexpressed in human atherosclerotic plaques[320] and is subject to m^{6}A methylation.[321]

A-to-I editing

ADAR1-dependent editing and increased expression of vascular inflammation-associated cathepsin S (CTSS), an extracellular matrix-cleaving protease,[50,322,323] was found by Stellos et al. in patient samples from coronary and carotid atherosclerotic arteries and aortic aneurysms as well as in hypoxic and inflamed (TNF-α and interferon gamma [INF-γ]) endothelial cells. Moreover, based on a finding of reduced CTSS expression in atherosclerotic plaques of minipigs treated with anti-oxLDL antibody,[324] its atherosclerotic plaque-stabilizing mechanism was hypothesized to be mediated by ADAR1 A-to-I editing of *Ctss* mRNA.[102] In peripheral blood mononuclear cells (PBMCs), the editing of the lncRNA nuclear paraspeckle assembly transcript 1 (NEAT1) by ADAR1 led to its stabilization and increased expression, which then positively correlated with the level of atherosclerotic disease.[103] Further, in TNF-α-activated human umbilical vein endothelial cells (HUVECs), NEAT1 knockdown blunted the mRNA expression of proatherosclerotic chemokines C-C motif chemokine ligand 2 (*CCL2*), C-X-C motif chemokine ligand 8 (*CXCL8*), and adhesion molecules *ICAM-1* and *VCAM-1*.[103] Together with increased *Adar1* mRNA levels, the levels of *EndoV* mRNA and inosine have been reported to be upregulated in human carotid atherosclerotic plaques.[73] Moreover, as both reduced atherosclerotic plaque monocyte infiltration and size were observed in double $ApoE^{-/-}$ $EndoV^{-/-}$ knockout mice, the inhibition of the seemingly proatherosclerotic ENDOV might prove a therapeutic strategy.[73] Also, as the specific interplay between ADAR1, inosines within various RNAs, and ENDOV in atherosclerosis remains elusive, such targeted considerations would be of great interest in light of the above findings and the following notions. In fact, cluster of differentiation 47 (CD47), an important proatherosclerotic and antiphagocytic immunoglobulin,[325] was measured as potently downregulated in $EndoV^{-/-}$ macrophages *ex vivo*, despite harboring no sites for A-to-I editing.[73] Hence, it justifiable to postulate RNA editing-independent functions for ENDOV in atherosclerosis. Indeed, rather than acting as an inosine-specific endonuclease, as established *in vitro*,[54] ENDOV has recently been suggested to preferentially bind RNAs to protect them from degradation *in vivo*.[326]

Atherosclerosis pathophysiology as a multiorgan systemic process

The emerging epitranscriptomic insight regarding atherosclerosis is justifiably heavily plaque focused. However, contributions of other organs and cells to the progression of the atherosclerotic lesions—alongside the emanated paracrine and endocrine signals from these disease foci—are needed to form a more comprehensive view of epitranscriptomic regulation in atherosclerosis. The organs shown to respond to such systemic signals of atherosclerosis are the liver, adipose tissue, bone marrow, and the lymphoid organs. Increased proliferation of the bone-marrow-residing hematopoietic stem cells[327,328] and their splenic invasion[329] establishing extramedullary hematopoiesis are such key extravascular processes. These processes then seed into circulation pro-inflammatory monocytes with plaque-invasive and plaque-promoting properties.[328,329] Such a vicious cycle of atherosclerosis has recently been detailed in terms of causation so that the atherosclerotic process itself increases the proliferation rate of hematopoietic stem cells, thus accelerating the efflorescence of proatherogenic clonal hematopoiesis.[330] As accumulating research

also indicates both m6A[331–334] and A-to-I[69] pathways to play crucial roles in regulating the proliferation and differentiation of hematopoietic stem cells, studies focusing on the role of epitranscriptomics within these systemic aspects of atherosclerosis are warranted.

Myocardial hypoxia, infarction, and fibrosis

Although m6A has been associated with hypoxia-reoxygenation (H/R) injury, myocardial hypoxia, ischemia, ischemia-reperfusion (I/R) injury, and post-ischemic fibrosis, reports specifically addressing A-to-I editing in these conditions remain very scarce. Stable expression of ADAR2 has been recently reported, however, both shortly and 3 weeks after MI.[146] Remarkably, ADAR2 overexpression unveiled a phenotype of improved cardiac healing after MI.[146] Namely, abolished functional deterioration, infarct size, fibrosis, and necrosis, as well as cardiomyocyte-specific increase in proliferative markers, were demonstrated.[146] As detailed above (section "cardiogenesis and cardiac regeneration"), the underlying mechanism was suggested to operate via an A-to-I editing-dependent inhibition of pri-miR-34a maturation.[146]

m6A modification

Reduced myocardial FTO expression has been reported after MI both in humans and mice,[107,133] and cardiomyocyte-targeted FTO overexpression in mice has been shown to reduce myocardial ischemic damage.[107] In hypoxic cardiomyocytes, FTO overexpression counteracted dysfunctional intracellular Ca^{2+} oscillations, increased contractility, reduced arrhythmic events, and increased both *Serca2* mRNA and protein expression. FTO expression reversed the hypermethylation observed in failing cardiomyocytes.[107] Interestingly, Mathiyalagan et al. also showed that the hypermethylation of *Ryr2* mRNA, a major mediator of cardiac sarcoplasmic calcium-induced calcium release that is imperative for proper propagation of electrical impulses, was attenuated by FTO overexpression in infarcted myocardium.[107] However, the subsequent impact on the respective protein levels remained elusive.

In response to AngII-induced activation of cardiac fibroblasts, reduced expression of circular RNA CUGBP Elav-like family member 1 (circCELF1), also seen in plasma samples of MI patients,[335] was reported to drive the downregulation of FTO.[149] It was mechanistically further revealed that the FTO downregulation led to m6A hypermethylation of Dickkopf WNT signaling pathway inhibitor 2 (*DKK2*) mRNA, which enhanced its miR-636-mediated degradation and promoted a profibrotic cellular phenotype. The therapeutic effect of this pathway was confirmed by DKK2 and miR-636 antagomir overexpression during experimental MI.[149] To identify novel m6A-based post-MI angiogenesis-promoting and fibrosis-restricting therapies, it will be of great interest to assess this FTO-dependent molecular pathway, perhaps with an additional focus on the m6A-regulated angiogenic Wnt/β-catenin signaling pathway (see discussion in section "angiogenesis") in myocardial vasculature.[336] Moreover, finer dissection of the revealed positive upstream regulation of FTO by circCELF1, including, for instance, identifying *FTO* targeting miRNAs sponged by this circRNA, could offer novel druggable targets.

In line with the observed beneficial effects of FTO for ischemic cardiomyocytes, cardiac fibroblasts, and myocardium, similar to the above discussions on hypertrophy and HF, Shen et al. identified FTO to reduce cardiomyocyte apoptosis after H/R injury via demethylation of a lncRNA myosin heavy-chain-associated RNA transcript (Mhrt).[135] Of further therapeutic interest, Mhrt, alongside another myocardium-specific lncRNA cardiac hypertrophy-associated transcript (CHAST), has earlier been measured hypermethylated in a murine infarcted myocardium and to demethylate after local myocardial FTO silencing yielding a beneficial phenotype.[107] Indeed, Mhrt is a cardiac-specific lncRNA transcribed from the antisense strand of *Myh7* gene with a protective role against hypertrophy by sequestering the brahma-related gene-1 (*Brg1*) mRNA to consequently blunt the prohypertrophic transition from myosin heavy chain 6 (MYH6) to myosin heavy chain 7 (MYH7) expression dominance.[337,338] Interestingly, triiodothyronine upregulates Mhrt in I/R-injury[339] and has been linked to predict MI[340] and HF.[341] Other m6A-modified lncRNAs expressed in ischemic myocardium, such as long-chain non-coding RNA metastasis-related lung adenocarcinoma transcript 1 (*MALAT1*), have also been suggested as future therapeutic targets for myocardial reperfusion injury.[342]

A functional intersection for the METTL3 writer and ALKBH5 m6A eraser was established by Song et al. in cardiomyocyte H/R and I/R injuries.[134] The authors reported increased m6A levels and expression of METTL3 to adversely associate with decreased autophagic flux and increased apoptosis.[134] Furthermore, a master regulator of apoptosis, transcription factor EB (TFEB), was shown not only to be regulated by METTL3 but also to regulate METTL3 and ALKBH5 in cardiomyocytes and myocardium after I/R injury. Specifically, a negative-feedback loop with two arms was discovered, where first METTL3-mediated m6A-methylation of *Tfeb* pre-mRNA attracts the indirectly m6A-binding heterogeneous nuclear ribonucleoprotein D (HNRNPD, alias AUF1 [ARE/poly(U)-binding/degradation factor 1], measured overexpressed in human failing heart[343]) to increase its translation and consequently TFEB protein expression. The consequent binding of TFEB to *Alkbh5* gene promoter is enhanced, resulting in enhanced *Alkbh5* gene transcription and ALKBH5 protein expression. Second, rather than controlling transcription, TFEB was discovered to destabilize *Mettl3* mRNA, thus downregulating its own positive upstream regulator.[134]

METTL3 therefore appears to be a detrimental agent in murine hypoxic cardiomyocytes *in vitro* and in infarcted myocardium, akin to the earlier discussion for cardiac hypertrophy. These findings support a rationale that METTL3 inhibitors may act as putative therapeutic agents in IHD.[344,345] Such postulation is strengthened by the findings that (1) METTL3 knockout before mice MI preserves cardiac function and structure afterward,[140] and (2) METTL3 promotes cardiomyocyte pyroptosis and myocardial I/R injury in rats via an m6A-dependent DGCR8-mediated pri-miR-143-3p maturation to yield miR-143-3p to finally suppress protein kinase C epsilon type (PRKCE).[142] However, myocardial METTL3 overexpression has also been implicated with cardioprotective ability by (1) inducing

therapeutic myocardial angiogenesis in mice shortly after MI,[56] (2) lessening post-MI damage in rats by promoting cardiomyocyte proliferation via stimulated pri-miR-17-3p maturation,[147] and (3) its non-catalytic METTL14 subunit protecting mice heart from extensive I/R injury by activating Wnt1/β-catenin signal pathway via an m^{6}A-dependent enhanced Wnt family member 1 (*Wnt1*) mRNA translation.[143] Hence, it can be postulated that the resulting final effect on the cardiac phenotype depends considerably on the relative weights of METTL3 activity within the distinct cardiac cell types, ischemic models, as well as the expression profile of the m^{6}A readers and transcribed transcriptome available for methylation at a given time, as discussed above (see section "cardiac hypertrophy and failure").

Hypoxia-inducible m^{6}A deposition by METTL3 was recently described to operate specifically in cardiomyocytes, which could possibly provide an operating rationale for some of the above speculations.[144] Namely, a hypoxia-inducible, cardiomyocyte-enriched, and mesoderm-restricted upregulation of a nuclear cap-binding subunit 3 (NCBP3) protein was identified to occupy the 5′ UTRs of 85 distinct mRNAs in hypoxic cardiomyocytes with a striking 87.6% congruency to a previously published hypoxic cardiomyocyte dataset of transcripts with incongruent translation activity to their transcriptomic expression.[346] NCBP3 was shown to recruit METTL3, promoting the bound mRNA m^{6}A methylation and eukaryotic translation initiation factor 4A2 (eIF4A2) to initiate their translation.[144]

Furthermore, ALKBH5 overexpression has also been shown beneficial by enhancing cardiac regeneration and salvage myocardial function after MI in both neonatal and adult mice (see section "cardiogenesis and cardiac regeneration") via m^{6}A demethylation-dependent increase in *Ythdf1* translation and consequent YTHDF1-dependently enhanced *Yap1* translation to YAP1.[54] This YTHDF1-m^{6}A-*Yap1* interaction was confirmed operative irrespective of ALKBH5 activity when YTHDF1 was overexpressed, suggesting incapability of ALKBH5 to demethylate *Yap1*. FTO, in addition to its anti-apoptotic effects in H/R-treated cardiomyocytes,[135] has also been indicated with an age-dependent waning and consequently propagated ischemic myocardial injury.[138] In H/R-injured neonatal cardiomyocytes, FTO overexpression upregulated YAP1 via m^{6}A-demethylation-mediated protection of *Yap1* mRNA from degradation.[145] While the phenotype of these H/R-injured neonatal cardiomyocytes appears analogously therapeutic with ALKBH5 overexpression,[145] the distinct epitranscriptomic pathways converging at *Yap1* translation suggest FTO selectivity as an m^{6}A eraser for *Yap1* with simultaneous m^{6}A reader milieu that promotes the degradation of m^{6}A-methylated *Yap1*. Hence, it will be of interest to assess the capacity of FTO to demethylate *Yhdf1* mRNA and consequently regulate the protein.

On the other hand, WTAP, a METTL3 writer complex subunit,[136] has been associated with adverse effects in ischemic myocardium. Namely, an ischemic damage and ER-stress-promoting pathway was identified, where WTAP, via activating transcription factor 4 (*Atf4*) mRNA m^{6}A methylation, upregulates ATF4 and promotes cardiomyocyte injury.[136] Therapeutically, WTAP knockout effectively restricted the injuries.[136] Moreover, the WTAP overexpression-induced cardiomyocyte ER stress and apoptosis during H/R injury was effectively ameliorated *in vitro* with administration of 4-phenylbutyric acid (4-PBA), an ER stress inhibitor.[136] Interestingly, in global *Mettl114*$^{+/-}$ mice with worsened I/R-injury phenotype compared with controls, WTAP was identified as the only differentially expressed, i.e., upregulated, m^{6}A writer subunit.[143] Finally, based on bioinformatic reanalysis of up to 108 ischemic, 16 non-ischemic, and 86 idiopathic human myocardium specimens, WTAP was also identified as the most consistently upregulated of the m^{6}A governing enzymes.[133] Hence, WTAP might be unveiled as a biomarker in human ischemic cardiac pathologies.

Aortic valve calcification

Aortic valve calcification is the most common progressing cause of aortic stenosis in the industrialized world.[347] In Europe and North America, aortic stenosis is estimated to affect up to 12.4% of the population over 75 years of age with a staggering prevalence of 3.4% for such critical disease that surgical intervention is guideline-mandated.[347] Macroscopically, progressing stenosis narrows the valve orifice and drives cardiac hypertrophy.[348] Microscopically, the valve calcification is characterized by osteoblast-like phenotype conversion of the valve interstitial cells, ROS production, calcium deposition, and activation of resident valve endotheliocytes as well as leukocyte diapedesis.[349]

m^{6}A and A-to-I modifications

Only two studies have addressed m^{6}A and A-to-I modifications in the pathophysiology of aortic valve calcification. In the first, the m^{6}A modification was described to control the phenotype conversion of human aortic valve interstitial cells to osteoblast-like cells via METTL3-mediated methylation and consequent YTHDF2-dependent degradation of twist family basic helix-loop-helix (bHLH) transcription factor 1 (*TWIST1*) mRNA, which ultimately downregulated the protein.[157] The other case study has described three children, all with tricuspid aortic valves and biallelic loss-of-function mutations in the *ADAR1* gene, who developed systemic class I interferonopathy with pronounced early-age-onset aortic valve calcification, stenosis, and HF.[156]

Angiogenesis

Angiogenesis has been heavily implicated in epitranscriptomic control of both m^{6}A and A-to-I editing. Especially modifications of miRNAs and their altered targetome have been unveiled as important. An overview of the key results from the field of non-malignant angiogenesis is offered below. For more detailed insight into epitranscriptomic control of neovascularization, the reader is directed toward recent reviews for m^{6}A[350] and A-to-I editing.[68]

m^{6}A modification

Corneal angiogenesis is inhibited in FTO knockout mice.[58] Alike, the tube formation of HUVECs in either unstressed or H_2O_2-stressed

conditions shrinks via a putative focal adhesion kinase (*FAK*)-m^{6}A-YTHDF2 axis.[58] Accordingly, FTO overexpression has been associated with enhanced post-ischemic myocardial angiogenesis, albeit with light methodological evidence (see the next paragraph).[107] On the other hand, silencing of the other m^{6}A eraser, ALKBH5, promoted angiogenesis in a hindlimb ischemia model.[104] The angiogenesis-repressing function of ALKBH5 was associated with increased m^{6}A methylation and stability of Wnt family member 5A (*Wnt5A*) mRNA in hypoxia-treated cardiac microvascular endothelial cells (CMECs).[104] No responsible m^{6}A readers were identified, however. Moreover, as the role of WNT5A in angiogenesis remains controversial,[351] it is plausible to speculate the molecular network to be more complex. Increased expression of ALKBH5 has also been reported in HUVECs and human microvascular endothelial cells (HMVEs) after lipopolysaccharide and hypoxia, consistent with results for hypoxic CMECs.[104] However, rather than disrupting angiogenesis, upregulated ALKBH5 was shown to sustain it. In detail, ALKBH5 was found to maintain sphingosine kinase 1 (SPHK1) expression by reducing *SPHK1* mRNA m^{6}A methylation, and to preserve both endothelial nitric oxide synthase (eNOS) and protein kinase B (PKB), alias AKT, phosphorylation.[109] Here, it is worth noting the methodological differences. Indeed, with the above lipopolysaccharide and hypoxia protocol,[109] the authors could not replicate the well-established vascular endothelial growth factor (VEGF)-A induction in these cells when singly stimulated by these stressors.[352,353]At the same time, such findings also suggest a relatively conserved endothelial hypoxia response to upregulate ALKBH5. Such dynamics appear distinct from the measured downregulation of FTO[107] and ALKBH5[54] in infarcted myocardium.

Regarding the role of m^{6}A writers, overexpression of METTL3 *in vitro* has been shown to increase angiogenic parameters in both HUVECs and human CMECs (HCMECs) during basal conditions,[56] and endothelial progenitors under hypoxia.[108] Namely, in HUVECs and HCMECs, METTL3 increased the m^{6}A methylation of let-7e-5p and miR-17-92 clusters, which were subsequently shown to downregulate antiangiogenic thrombospondin 1 (TSP1).[56,354-355] METTL3 overexpression was also reported to increase angiogenesis in experimental models of MI and hindlimb ischemia *in vivo*.[56] As the authors pointed out, with regard to general effects on m^{6}A in RNA, their findings are contradictory to the increased myocardial angiogenesis observed with FTO overexpression following MI.[107] However, the robust methodological variation limits the interpretation of the results. Specifically, with the FTO overexpression, angiogenesis was assessed with a single-antibody staining against platelet endothelial cell adhesion molecule (PECAM-1), alias cluster of differentiation 31 (CD31), positive endotheliocytes 4 weeks after MI, a time point at which post-MI healing and angiogenesis have mostly taken place already. Notably, HIF-1α has been pinpointed as a positive upstream regulator of proangiogenic METTL3 expression in hypoxic endothelium *in vitro*.[57] Specifically, METTL3 was identified to mediate its proangiogenic role in a YTHDF1-dependent manner by enhancing the translation of m^{6}A-methylated low-density lipoprotein receptor-related protein 6 (*LRP6*) and disheveled segment polarity protein 1 (*DVL1*) mRNAs.[57] Both targets, alongside the discussed *Wnt5a* mRNA,[104] encode proteins that are part of the Wnt signaling pathway, a core regulator pathway of angiogenesis in endotheliocytes.[356,357]

Interestingly, decreased METTL3[106] and WTAP[105] expression has been associated with larger diameters of human cerebral arteriovenous malformations. Regarding m^{6}A writer METTL3, Wang et al. pinpointed two putative mechanistic pathways for promoting angiogenesis *in vitro*. The first pathway involves METTL3-mediated stabilization of deltex E3 ubiquitin ligase 3L (*DTX3L*) mRNA in an m^{6}A-IGF2BP1/3-dependent manner to enable the respective DTX3L protein to heterodimerize with deltex E3 ubiquitin ligase 1 (DTX1) to form a Notch E3 ubiquitin ligase, which suppresses Notch signaling and further downstream hes-related family bHLH transcription factor with YRPW motif 2 (HEY2).[106] In the second suggested mechanism, METTL3 represses the transforming growth factor β1 (TGF-β1) pathway via SMAD (homologs of the *Drosophila melanogaster* protein 'mothers against decapentaplegic' (MAD) and *Caenorhabditis elegans* 'small body size' (SMA)) family member 6 (SMAD6) downregulation and increases phosphorylation of its other members, including SMAD1-3, SMAD5, and SMAD9.[106] WTAP deficiency was noted also to increase free WT1 expression, which led toWnt signaling inhibition and increased degradation of β-catenin.[105] WTAP has also been reported to maintain angiogenic desmoplakin (DSP)[358–360] expression in endothelial cells in an m^{6}A-IGF2BP1/3-dependent manner.[105]

A-to-I editing

van der Kwast et al. discovered that miR-487b, previously known to maintain the integrity of hypertensive artery walls and post-ischemic blood flow recovery,[361] was increasingly edited from its seed sequence in hindlimb ischemia.[65] The edited form, miR-487b-ED, was unveiled to have unique proangiogenic functions and a near-completely altered targetome compared with the unedited miR-487b. In addition, four other vasoactive and vascular-cell-expressed miRNAs have been established to be the targets for notable A-to-I editing: miR-376a-3p, miR-376c-3p, miR-381-3p, and miR-411-5p.[66,67] These miRNAs were shown to contain inosine edits in their seed sequence at the maturation stage and to respond to hypoxia by increased editing. These targetomes of these edited forms acted to promote angiogenesis.[66,67] Collectively, these findings delineate a novel layer of ischemic angiogenesis regulation and elucidate avenues for epitranscriptomics-based angiogenic miRNAs to be tested as therapeutic handles.

Arterial aneurysms

Arterial aneurysms represent a set of conditions with variable risk factors and etiologies.[362–364] All are characterized by the disruption of the structural and mechanical properties of the arterial wall.[365–367] This leads to local ballooning of an artery with concurrent thinning of its wall rendering the artery prone to dissection[368] and rupture.[369–370] In general, aneurysm ruptures are associated with extremely high mortality rates.

A-to-I editing

Increased expressions of the A-to-I editor *ADAR1* and *CTSS* mRNAs have been described in human aneurysmatic thoracic aortas. The authors identified the RNA-stabilizing HuR to bind the newly formed inosine.[50] Importantly, CTSS has many matrix remodeling functions and participates in both collagenolysis and elastolysis,[371–373] which are processes also heavily implicated in aneurysm pathophysiology,[362–364,367] and could provide a pharmacologically targetable molecular pathway. Based on the discovery for A-to-I editing to control diastolic blood pressure via *Flna* mRNA editing,[116] thus producing an actin crosslinking FLNA, heavily implicated in arterial wall integrity (see also section "hypertension"),[252] further investigations regarding A-to-I editing in also controlling aneurysm pathophysiology are warranted and rational.

m^{6}A modification

Aging has been shown to downregulate METTL3 expression in the aorta.[156] The development and progression of abdominal aortic aneurysm has been suggested to be induced through METTL3-mediated maturation of miR-34a and decreased Sirtuin 1 (*Sirt1*) mRNA expression.[86] Accordingly, knockdown of METTL3 protects from development of abdominal aortic aneurysm, and this therapeutic effect is inhibitable by either miR-34a silencing or SIRT1 overexpression.[86] Interestingly, a recent study highlighted the SIRT1-melatonin axis in a murine thoracic aortic aneurysm model.[374] The authors noted that melatonin administration prevented thoracic aortic aneurysm formation via acting on SIRT1 in a melatonin-receptor-dependent manner.[374] Yang et al. reported melatonin to inhibit METTL3 expression and m^{6}A in ESCs specifically via melatonin receptor 1 (MT1) and further through the MT1–Janus kinase 2 (JAK2)–STAT3–zinc-finger protein 217 (Zfp217) pathway,[375] all members of which are implicated to be regulated by m^{6}A in CVDs.[118,376] Moreover, melatonin has been shown to downregulate VEGF in hypoxic retinas,[377–379] inhibit hypoxic angiogenesis by repressing the HIF-1α-VEGF-ROS axis,[380] and upregulate HIF-1α targeting miR-3195 and miR-374b, thus downregulating VEGF.[381] These findings are of interest considering the upstream roles of melatonin and MT-1 in inhibiting METTL3,[375] since *Vegf* mRNA has been shown to be m^{6}A modified by METTL3, thus modulating a TEK receptor tyrosine kinase (TEK)–PI3K–VEGF axis via increasing its stability and enhancing angiogenesis.[382] Furthermore, the m^{6}A-reader IGF2BP3 has been shown to bind m^{6}A in *Vegf* mRNA, increasing its translation.[383] To summarize, as VEGF inhibition has been shown to prevent aortic aneurysm progression,[384] melatonin might, via *Vegf* mRNA m^{6}A methylation, act on aneurysm development.

Dissected aortas were reported to have decreased KIAA1429 (alias *VIRMA*) and miR-143-3p levels while ALKBH5 was reported upregulated.[88] KIAA1429 was shown, via its increasing m^{6}A methylation effect, to enhance pri-miR143-3p maturation by interacting with the important miRNA molecular processor DCGR8 to consequently downregulate its downstream target gene, responsible for observed phenotypes, DEAD-box helicase 6 (DDX6). On the other hand, ALKBH5 was shown to repress such interaction and thus, contrary to the phenotypes observed in KIAA1429 overexpression, promote aortic dissection, suppress human aortic smooth muscle cell (HASMC) proliferation, and promote apoptosis in human aortic endothelial cells (HAECs).[91]

He et al. demonstrated increased m^{6}A content and expressions of YTHDF2 and YTHDF3 in abdominal aortic aneurysm,[82] of which YTHDF3 positively correlated with the aneurysm diameter. Another similar associative study has linked reduced METTL14 expression with higher risk of aneurysm rupture.[84] Like ALKBH5,[91] FTO is also upregulated in dissecting and stable aortic aneurysms.[87] Moreover, increased angiotensin-II-induced FTO levels and gain-of-function methods in VSMCs were shown to mediate pathologic phenotype switching.[87] Mechanistically, the FTO-driven demethylation of Krüppel-like factor 5 (*KLF5*) mRNA and downregulation of glycogen synthase kinase 3β (GSK3β) signaling, leading in combination to upregulated KLF5 protein, were unveiled as the responsible pathways.[87] Interestingly, the adverse role of KLF5 has also been shown in atherosclerosis as a part of oxLDL–KLF5–miR-29a–F-box and WD repeat domain containing 7 (FBW7) positive feedback loop. Specifically, oxLDL-induced upregulation of KLF5, further accelerated via miR-29a accumulation-mediated and FBW7-repression-dependent reduction of KLF5 ubiquitination, increases VSMC proliferation and progression of atherosclerosis, thus stressing miR-29a suppression as a possible therapeutic strategy.[385] Epitranscriptomically, it is interesting that miR-29a has been shown to undergo m^{6}A methylation and to be consequently repressed by the HNRNPA2/B1 m^{6}A reader.[386] KLF5 has been implicated as a key hub for vascular-injury-induced proliferative responses of VSMCs, neointima formation, as well as both angiotensin II-induced cardiac hypertrophy and fibrosis response.[387] Combined, targeted hypermethylation of either the *KLF5* mRNA or miR-29a might prove therapeutic in conditions where VSMC hyperproliferation holds a central pathophysiologic role, including atherosclerosis, arterial aneurysms, and both pulmonary and systemic hypertension.

FUTURE PERSPECTIVES

Taken together, both the m^{6}A and A-to-I modifications have emerged as dominant regulators of CVDs. Some future perspectives are discussed below.

Drugging the m^{6}A- and A-to-I-related pathways in cardiovascular diseases

A number of compounds targeting m^{6}A writers[345,393] and erasers[394–396] have been identified. A summary of these small molecules is provided in Table S5. As many of these well-characterized molecules remain untested in cardiovascular models (excluding the FTO inhibitors FB23-2 and Rhein;[117] see section "hypertension") and, based on the above discussions, the function of the m^{6}A regulators varies across tissues, cell types, and diseases (as well as their models), their testing within the cardiovascular field appears to be a promising avenue to extend our evolving understanding. Pharmacological evaluation could reveal insightful sum effects of these regulators; for example, for METTL3 in the context of cardiac hypertrophy, MI, and flow-induced

endothelial dysfunction. Consequently, coupled with accumulating new evidence, validations, and methodological standardization, such insights could help us to detangle these currently complex, even controversial, mechanistic landscapes currently hallmarked by simultaneous and extensive involvement of METTL3-mediated m^{6}A methylation within multiple—even opposing—molecular pathways. Moreover, as these epitranscriptomic regulators have demonstrated both driving and suppressing roles in some of these pathologies, these compounds may also provide novel therapeutic benefits. For example, as a METTL3 inhibitor is emerging a handle to treat acute leukemia,[345] and while YTHDF2 also appears another such target,[397] testing this METTL3 inhibitor, STM2457, during MI or hypertrophy might produce therapeutic effects. Moreover, as upregulation of FTO and ALKBH5 has most consistently proved to be therapeutic during various models of myocardial ischemia, development of activating compounds for these m^{6}A erasers could hold translational therapeutic value to mend ischemic hearts. Finally, the additional discovery of such compounds for the m^{6}A readers and A-to-I editors is awaited.

m^{6}A readers as a key to clarify the role of epitranscriptomics in CVD pathologies?

Many of the numerous readers of m^{6}A remain uncharacterized.[388–392] Due to the nature of these readers, responding to upstream stimuli to initiate the molecular, cellular, and ultimately systemic responses, it is probable that they will prove to be centrally important in CVD epitranscriptomics. Encouragingly, studies exploring the role of these readers in CVDs are being constantly reported (Figures 3, 4, 5, 6, and 7).

Although the YTHDF family of m^{6}A readers has recently attracted considerable scientific interest, the redundancy of their targets and downstream functions remains a matter of recent controversy. Namely, two research groups, led by Jaffrey et al.[264] and Hanna et al.,[398] have recently called into question the canonical scheme where YTHDF1 stabilizes the m^{6}A-bound transcripts, YTHDF2 degrades them, and YTHDF3 can act in both directions. According to this view, the YTHDF family shares a virtually identical set of modified target RNAs and functions in unity to promote their degradation. However, many both m^{6}A- and YTHDF1-dependently stabilized mRNAs, as measured in cardiovascular tissues, seem to be in contradiction with such a scheme stemming from HeLa cells[264] and ESCs.[398] These include *NLRP1*,[92] *FOXO1*,[90] *Myl2*,[121] *Atf4*,[136] *MAGED1*,[80] and *Wnt5a*.[104] In addition, YTHDF2, but neither YTHDF1 nor YTHDF3, has been described to bind m^{6}A-modified *Acvr2a* mRNA in hypertrophic skeletal muscle.[262] Notably, a recent preprint by Zou et al.[400] has brought up major experimental controversies in the original paper published by Zaccara and Jaffrey.[264] Also, based on revised and additional experiments, YTHDF1 was demonstrated to promote translation of its target mRNAs in HeLa cells and to harbor a low-complexity domain notably distinct from that of YTHDF2, which was found to account for their capacity to form different condensates and to act in a paralog-specific manner.[400]

Elucidation of the upstream control of m^{6}A readers may also help to explain the possible discrepancy between YTHDF family members. For example, post-transcriptional YTHDF2 SUMOylation considerably increases its affinity to m^{6}A, thus stimulating the m^{6}A-bound RNA degradation in cancer cells.[399] Also, the importance of post-transcriptional regulation of METTL3,[267] METTL14, WTAP,[401] and YTHDFs in a paralog-specific manner[38] by phosphorylation has been described in non-cardiovascular systems. Finally, to truly unravel the functions YTHDF paralogs, the importance of paralog-specific expressions according to given tissue, cell, and cell state, or even specific molecular signals, cannot be overemphasized. For example, the knockdown of YTHDF1 or YTHDF3 in ESC-derived cardiomyocytes, contrary to ESCs, does not accumulate m^{6}A in RNAs,[160] and tumor protein 63 (p63) seems to upregulate just YTHDF3 in skin.[402] Hence, it is critical that the functions of YTHDF paralogs are also meticulously examined in the cardiovascular system. Such an approach could also provide clarification to the contrasting findings for METTL3-mediated m^{6}A methylation in controlling the cardiac hypertrophy and oscillatory flow-induced endothelial dysfunction discussed above.

Revealing the upstream control of ADARs in cardiovascular systems

While the understanding of the role of A-to-I editing in cardiovascular diseases remains limited in general, overall highlighting the need for future investigations, deciphering its upstream control might provide avenues for novel considerations. For example, miR-1 has been shown to target ADARs by repressing their expression in non-cardiac cells.[300,301] However, although this miRNA is highly expressed in heart[296] and has an established role in many CVDs or related processes,[298,299,302,305–307] its role from an epitranscriptomic viewpoint remains to be established in heart.

Cardiometabolism is a putative modulator of the cardiac m^{6}A methylome

As oxygen and α-ketoglutarate are needed to erase m^{6}A, and the reaction yields succinate (Figure 1),[38] the metabolic state of the myocardium can be expected to affect its RNA m^{6}A content. Indeed, in hypoxic myocardium, α-ketoglutarate depletes downstream in the Krebs cycle into succinate,[403] while it acts to produce high-energy phosphates as substituents for the deteriorating oxidative metabolism.[404] In addition, α-ketoglutarate acts upstream in the cycle to produce citrate, thus circumventing the tormented mitochondrial respiration to produce lipids for energy. While originally described in hypoxic cancer cells,[405] such a process may also alter myocardial epigenomes.[406] Last, as α-ketoglutarate levels are supplemented endogenously in hypoxia only via either glutamine or glutamate deamination, it seems consistent that exogenous supplementation of these metabolites protects heart from ischemia.[407–409]

Hence, epitranscriptomically, it appears congruent that m^{6}A levels rise as α-ketoglutarate depletes in myocardial ischemia.[107,134] Taken together, as overexpression of m^{6}A erasers protects myocardium from ischemic insults,[54,107] FTO is upregulated in cardiomyocytes by leptin adipokine[118] and conveys cardioprotection also via stimulation of glucose metabolism,[175] the role of hypoxic cardiometabolism

in controlling cardiac RNA m^6A dynamics, and vice versa, emerges as being worthy of future study.

CONCLUSIONS

An epitranscriptomic era is unfolding in translational RNA biology and medicine.[410] Here, we have reviewed the fast-growing body of evidence available regarding both initial associative and experimental findings to establish a firm link for the two most common epitranscriptomic modifications, m^6A and A-to-I, to mirror as well as partake in the onset and development of multiple common cardiovascular diseases. As our current mechanistic understandings can be expected to further crystallize in the future, the potential of modified endogenous RNAs as targets for future cardiovascular pharmacologic development will increase. In addition, as exquisitely positioned at the intersection of our transcribed genome and its ultimate interpretation as functional proteins, these relatively stable, yet dynamic,[411] modifications also hold potential for biomarker discovery. Prospective controlled observational cohort studies, such as the The Ischemic Heart Disease Epitranscriptomics and Biomarkers (IHD-EPITRAN) study (www.ihd-epitran.com), help us to shed light into this fascinating development.[412]

SUPPLEMENTAL INFORMATION

Supplemental information can be found online at https://doi.org/10.1016/j.omtn.2022.07.018.

ACKNOWLEDGMENTS

The authors acknowledge scientific writer Jennifer Rowland for spelling and grammar checks of the article. This work was supported by the Finnish Foundation for Cardiovascular Research (E.K. 200092 and V.S. 200174), Finnish Government-allocated block grants to specialty area (A.V. TYH2020340), Aarne Koskelo Foundation (V.S.), The Finnish Medical Foundation (V.S. 3857), The Ida Montin Foundation (V.S. 20210362), and The Eemil Aaltonen Foundation (V.S. 210212 K). BioRender.com was utilized for creating the graphics (University of Helsinki license). Open access funded by Helsinki University Library.

AUTHOR CONTRIBUTIONS

V.S. collected the related papers, drafted and wrote the manuscript, and drew illustrations. E.K. wrote and revised the manuscript and provided supervision. A.V. revised the manuscript. All authors read and approved the final manuscript.

DECLARATION OF INTERESTS

The authors declare no competing interests.

REFERENCES

1. Roth, G.A., Mensah, G.A., Johnson, C.O., Addolorato, G., Ammirati, E., Baddour, L.M., Barengo, N.C., Beaton, A.Z., Benjamin, E.J., Benziger, C.P., et al. (2020). Global burden of cardiovascular diseases and risk factors, 1990-2019: update from the GBD 2019 study. J. Am. Coll. Cardiol. *76*, 2982–3021. https://doi.org/10.1016/j.jacc.2020.11.010.

2. Vos, T., Lim, S.S., Abbafati, C., Abbas, K.M., Abbasi, M., Abbasifard, M., Abbasi-Kangevari, M., Abbastabar, H., Abd-Allah, F., Abdelalim, A., et al. (2020). Global burden of 369 diseases and injuries in 204 countries and territories, 1990-2019: a systematic analysis for the Global Burden of Disease Study 2019. Lancet *396*, 1204–1222. https://doi.org/10.1016/S0140-6736(20)30925-9.

3. Benjamin, E.J., Virani, S.S., Callaway, C.W., Chamberlain, A.M., Chang, A.R., Cheng, S., Chiuve, S.E., Cushman, M., Delling, F.N., Deo, R., et al. (2018). Heart disease and stroke statistics-2018 update: a report from the American heart association. Circulation *137*, e67–e492. https://doi.org/10.1161/CIR.0000000000000558.

4. European Heart Network (2017). European Cardiovascular Disease Statistics. http://www.ehnheart.org/cvd-statistics/cvd-statistics-2017.html. (Accessed 26 May 2021).

5. Libby, P. (2021). The changing landscape of atherosclerosis. Nature *592*, 524–533. https://doi.org/10.1038/s41586-021-03392-8.

6. Groenewegen, A., Rutten, F.H., Mosterd, A., and Hoes, A.W. (2020). Epidemiology of heart failure. Eur. J. Heart Fail. *22*, 1342–1356. https://doi.org/10.1002/ejhf.1858.

7. Jones, N.R., Roalfe, A.K., Adoki, I., Hobbs, F.D.R., and Taylor, C.J. (2019). Survival of patients with chronic heart failure in the community: a systematic review and meta-analysis. Eur. J. Heart Fail. *21*, 1306–1325. https://doi.org/10.1002/ejhf.1594.

8. Taylor, C.J., Ryan, R., Nichols, L., Gale, N., Hobbs, F.R., and Marshall, T. (2017). Survival following a diagnosis of heart failure in primary care. Fam. Pract. *34*, 161–168. https://doi.org/10.1093/fampra/cmw145.

9. Lippi, G., and Sanchis-Gomar, F. (2020). Global epidemiology and future trends of heart failure. AME Med. J. *5*, 15. https://doi.org/10.21037/amj.2020.03.03.

10. Sabatine, M.S., Giugliano, R.P., Keech, A.C., Honarpour, N., Wiviott, S.D., Murphy, S.A., Kuder, J.F., Wang, H., Liu, T., Wasserman, S.M., et al. (2017). Evolocumab and clinical outcomes in patients with cardiovascular disease. N. Engl. J. Med. *376*, 1713–1722. https://doi.org/10.1056/NEJMoa1615664.

11. Schwartz, G.G., Steg, P.G., Szarek, M., Bhatt, D.L., Bittner, V.A., Diaz, R., Edelberg, J.M., Goodman, S.G., Hanotin, C., Harrington, R.A., et al. (2018). Alirocumab and cardiovascular outcomes after acute coronary syndrome. N. Engl. J. Med. *379*, 2097–2107. https://doi.org/10.1056/NEJMoa1801174.

12. Täubel, J., Hauke, W., Rump, S., Viereck, J., Batkai, S., Poetzsch, J., Rode, L., Weigt, H., Genschel, C., Lorch, U., et al. (2021). Novel antisense therapy targeting microRNA-132 in patients with heart failure: results of a first-in-human Phase 1b randomized, double-blind, placebo-controlled study. Eur. Heart J. *42*, 178–188. https://doi.org/10.1093/eurheartj/ehaa898.

13. Abbott, B.G., Case, J.A., Dorbala, S., Einstein, A.J., Galt, J.R., Pagnanelli, R., Bullock-Palmer, R.P., Soman, P., and Wells, R.G. (2018). Contemporary cardiac SPECT imaging-innovations and best practices: an information statement from the American society of nuclear cardiology. J. Nucl. Cardiol. *25*, 1847–1860. https://doi.org/10.1007/s12350-018-1348-y.

14. Seetharam, K., and Lerakis, S. (2019). Cardiac magnetic resonance imaging: the future is bright. F1000Res. *8*. https://doi.org/10.12688/f1000research.19721.1.

15. Lederle, F.A., Kyriakides, T.C., Stroupe, K.T., Freischlag, J.A., Padberg, F.T., Jr., Matsumura, J.S., Huo, Z., and Johnson, G.R.; OVER Veterans Affairs Cooperative Study Group (2019). Open versus endovascular repair of abdominal aortic aneurysm. N. Engl. J. Med. *380*, 2126–2135. https://doi.org/10.1056/NEJMoa1715955.

16. Goyal, M., Menon, B.K., van Zwam, W.H., Dippel, D.W.J., Mitchell, P.J., Demchuk, A.M., Dávalos, A., Majoie, C.B.L.M., van der Lugt, A., de Miquel, M.A., et al. (2016). Endovascular thrombectomy after large-vessel ischaemic stroke: a meta-analysis of individual patient data from five randomised trials. Lancet *387*, 1723–1731. https://doi.org/10.1016/S0140-6736(16)00163-X.

17. Al-Ahmad, A., Yee, R., and Link, M.S. (2018). Contemporary review of the cardiovascular implantable electronic devices with future directions. Card. Electrophysiol. Clin. *10*, xv. https://doi.org/10.1016/j.ccep.2017.12.002.

18. Kisling, L.A., and J, M.D. (2021). Prevention strategies. In StatPearls (Treasure Island (FL)).

19. Libby, P., Buring, J.E., Badimon, L., Hansson, G.K., Deanfield, J., Bittencourt, M.S., Tokgözoğlu, L., and Lewis, E.F. (2019). Atherosclerosis. Nat. Rev. Dis. Primers *5*, 56. https://doi.org/10.1038/s41572-019-0106-z.

20. Pothineni, N.V.K., Subramany, S., Kuriakose, K., Shirazi, L.F., Romeo, F., Shah, P.K., and Mehta, J.L. (2017). Infections, atherosclerosis, and coronary heart disease. Eur. Heart J. *38*, 3195–3201. https://doi.org/10.1093/eurheartj/ehx362.

21. Jonsson, A.L., and Bäckhed, F. (2017). Role of gut microbiota in atherosclerosis. Nat. Rev. Cardiol. *14*, 79–87. https://doi.org/10.1038/nrcardio.2016.183.

22. Tang, W.H.W., Wang, Z., Levison, B.S., Koeth, R.A., Britt, E.B., Fu, X., Wu, Y., and Hazen, S.L. (2013). Intestinal microbial metabolism of phosphatidylcholine and cardiovascular risk. N. Engl. J. Med. *368*, 1575–1584. https://doi.org/10.1056/NEJMoa1109400.

23. Nemet, I., Saha, P.P., Gupta, N., Zhu, W., Romano, K.A., Skye, S.M., Cajka, T., Mohan, M.L., Li, L., Wu, Y., et al. (2020). A cardiovascular disease-linked gut microbial metabolite acts via adrenergic receptors. Cell *180*, 862–877.e22. https://doi.org/10.1016/j.cell.2020.02.016.

24. van den Hoogenhof, M.M.G., Pinto, Y.M., and Creemers, E.E. (2016). RNA splicing: regulation and dysregulation in the heart. Circ. Res. *118*, 454–468. https://doi.org/10.1161/CIRCRESAHA.115.307872.

25. Yu, S., and Kim, V.N. (2020). A tale of non-canonical tails: gene regulation by post-transcriptional RNA tailing. Nat. Rev. Mol. Cell Biol. *21*, 542–556. https://doi.org/10.1038/s41580-020-0246-8.

26. Saletore, Y., Meyer, K., Korlach, J., Vilfan, I.D., Jaffrey, S., and Mason, C.E. (2012). The birth of the Epitranscriptome: deciphering the function of RNA modifications. Genome Biol. *13*, 175. https://doi.org/10.1186/gb-2012-13-10-175.

27. Nishikura, K. (2016). A-to-I editing of coding and non-coding RNAs by ADARs. Nat. Rev. Mol. Cell Biol. *17*, 83–96. https://doi.org/10.1038/nrm.2015.4.

28. Waddington, C.H. (2012). The epigenotype. 1942. Int. J. Epidemiol. *41*, 10–13. https://doi.org/10.1093/ije/dyr184.

29. Johnson, T.B., and Coghill, R.D. (1925). Researches on pyrimidines. C111. the discovery of 5-methyl-cytosine in tuberculinic acid, the nucleic acid of the Tubercle bacillus. J. Am. Chem. Soc. *47*, 2838–2844. https://doi.org/10.1021/ja01688a030.

30. Vaughan, M.H., Jr., Soeiro, R., Warner, J.R., and Darnell, J.E., Jr. (1967). The effects of methionine deprivation on ribosome synthesis in HeLa cells. Proc. Natl. Acad. Sci. USA *58*, 1527–1534. https://doi.org/10.1073/pnas.58.4.1527.

31. Desrosiers, R., Friderici, K., and Rottman, F. (1974). Identification of methylated nucleosides in messenger RNA from Novikoff hepatoma cells. Proc. Natl. Acad. Sci. USA *71*, 3971–3975. https://doi.org/10.1073/pnas.71.10.3971.

32. Dominissini, D., Moshitch-Moshkovitz, S., Schwartz, S., Salmon-Divon, M., Ungar, L., Osenberg, S., Cesarkas, K., Jacob-Hirsch, J., Amariglio, N., Kupiec, M., et al. (2012). Topology of the human and mouse m6A RNA methylomes revealed by m6A-seq. Nature *485*, 201–206. https://doi.org/10.1038/nature11112.

33. Meyer, K.D., Saletore, Y., Zumbo, P., Elemento, O., Mason, C.E., and Jaffrey, S.R. (2012). Comprehensive analysis of mRNA methylation reveals enrichment in 3' UTRs and near stop codons. Cell *149*, 1635–1646. https://doi.org/10.1016/j.cell.2012.05.003.

34. Garcia-Campos, M.A., Edelheit, S., Toth, U., Safra, M., Shachar, R., Viukov, S., Winkler, R., Nir, R., Lasman, L., Brandis, A., et al. (2019). Deciphering the "m(6)A code" via antibody-independent quantitative profiling. Cell *178*, 731–747.e16. https://doi.org/10.1016/j.cell.2019.06.013.

35. Xiao, Y., Wang, Y., Tang, Q., Wei, L., Zhang, X., and Jia, G. (2018). An elongation- and ligation-based qPCR amplification method for the radiolabeling-free detection of locus-specific N(6) -methyladenosine modification. Angew. Chem. Int. Ed. Engl. *57*, 15995–16000. https://doi.org/10.1002/anie.201807942.

36. Liu, H., Begik, O., Lucas, M.C., Ramirez, J.M., Mason, C.E., Wiener, D., Schwartz, S., Mattick, J.S., Smith, M.A., and Novoa, E.M. (2019). Accurate detection of m(6)A RNA modifications in native RNA sequences. Nat. Commun. *10*, 4079. https://doi.org/10.1038/s41467-019-11713-9.

37. Yang, Y., Hsu, P.J., Chen, Y.S., and Yang, Y.G. (2018). Dynamic transcriptomic m(6)A decoration: writers, erasers, readers and functions in RNA metabolism. Cell Res. *28*, 616–624. https://doi.org/10.1038/s41422-018-0040-8.

38. Zaccara, S., Ries, R.J., and Jaffrey, S.R. (2019). Reading, writing and erasing mRNA methylation. Nat. Rev. Mol. Cell Biol. *20*, 608–624. https://doi.org/10.1038/s41580-019-0168-5.

39. Bazak, L., Haviv, A., Barak, M., Jacob-Hirsch, J., Deng, P., Zhang, R., Isaacs, F.J., Rechavi, G., Li, J.B., Eisenberg, E., et al. (2014). A-to-I RNA editing occurs at over a hundred million genomic sites, located in a majority of human genes. Genome Res. *24*, 365–376. https://doi.org/10.1101/gr.164749.113.

40. Wei, C.M., and Moss, B. (1977). Nucleotide sequences at the N6-methyladenosine sites of HeLa cell messenger ribonucleic acid. Biochemistry *16*, 1672–1676. https://doi.org/10.1021/bi00627a023.

41. Wei, C.M., Gershowitz, A., and Moss, B. (1976). 5'-Terminal and internal methylated nucleotide sequences in HeLa cell mRNA. Biochemistry *15*, 397–401. https://doi.org/10.1021/bi00647a024.

42. van Tran, N., Ernst, F.G.M., Hawley, B.R., Zorbas, C., Ulryck, N., Hackert, P., Bohnsack, K.E., Bohnsack, M.T., Jaffrey, S.R., Graille, M., et al. (2019). The human 18S rRNA m6A methyltransferase METTL5 is stabilized by TRMT112. Nucleic Acids Res. *47*, 7719–7733. https://doi.org/10.1093/nar/gkz619.

43. Ueda, Y., Ooshio, I., Fusamae, Y., Kitae, K., Kawaguchi, M., Jingushi, K., Hase, H., Harada, K., Hirata, K., and Tsujikawa, K. (2017). AlkB homolog 3-mediated tRNA demethylation promotes protein synthesis in cancer cells. Sci. Rep. *7*, 42271. https://doi.org/10.1038/srep42271.

44. Mauer, J., Luo, X., Blanjoie, A., Jiao, X., Grozhik, A.V., Patil, D.P., Linder, B., Pickering, B.F., Vasseur, J.J., Chen, Q., et al. (2017). Reversible methylation of m(6)Am in the 5' cap controls mRNA stability. Nature *541*, 371–375. https://doi.org/10.1038/nature21022.

45. Aas, P.A., Otterlei, M., Falnes, P.O., Vågbø, C.B., Skorpen, F., Akbari, M., Sundheim, O., Bjørås, M., Slupphaug, G., Seeberg, E., et al. (2003). Human and bacterial oxidative demethylases repair alkylation damage in both RNA and DNA. Nature *421*, 859–863. https://doi.org/10.1038/nature01363.

46. Ougland, R., Zhang, C.M., Liiv, A., Johansen, R.F., Seeberg, E., Hou, Y.M., Remme, J., and Falnes, P.Ø. (2004). AlkB restores the biological function of mRNA and tRNA inactivated by chemical methylation. Mol. Cell *16*, 107–116. https://doi.org/10.1016/j.molcel.2004.09.002.

47. Li, X., Xiong, X., Wang, K., Wang, L., Shu, X., Ma, S., and Yi, C. (2016). Transcriptome-wide mapping reveals reversible and dynamic N(1)-methyladenosine methylome. Nat. Chem. Biol. *12*, 311–316. https://doi.org/10.1038/nchembio.2040.

48. Macbeth, M.R., Schubert, H.L., Vandemark, A.P., Lingam, A.T., Hill, C.P., and Bass, B.L. (2005). Inositol hexakisphosphate is bound in the ADAR2 core and required for RNA editing. Science *309*, 1534–1539. https://doi.org/10.1126/science.1113150.

49. Agranat, L., Sperling, J., and Sperling, R. (2010). A novel tissue-specific alternatively spliced form of the A-to-I RNA editing enzyme ADAR2. RNA Biol. *7*, 253–262. https://doi.org/10.4161/rna.7.2.11568.

50. Stellos, K., Gatsiou, A., Stamatelopoulos, K., Perisic Matic, L., John, D., Lunella, F.F., Jaé, N., Rossbach, O., Amrhein, C., Sigala, F., et al. (2016). Adenosine-to-inosine RNA editing controls cathepsin S expression in atherosclerosis by enabling HuR-mediated post-transcriptional regulation. Nat. Med. *22*, 1140–1150. https://doi.org/10.1038/nm.4172.

51. Vik, E.S., Nawaz, M.S., Strøm Andersen, P., Fladeby, C., Bjørås, M., Dalhus, B., and Alseth, I. (2013). Endonuclease V cleaves at inosines in RNA. Nat. Commun. *4*, 2271. https://doi.org/10.1038/ncomms3271.

52. Wang, X., Lu, Z., Gomez, A., Hon, G.C., Yue, Y., Han, D., Fu, Y., Parisien, M., Dai, Q., Jia, G., et al. (2014). N6-methyladenosine-dependent regulation of messenger RNA stability. Nature *505*, 117–120. https://doi.org/10.1038/nature12730.

53. Wang, X., Zhao, B.S., Roundtree, I.A., Lu, Z., Han, D., Ma, H., Weng, X., Chen, K., Shi, H., and He, C. (2015). N(6)-methyladenosine modulates messenger RNA translation efficiency. Cell *161*, 1388–1399. https://doi.org/10.1016/j.cell.2015.05.014.

54. Han, Z., Wang, X., Xu, Z., Cao, Y., Gong, R., Yu, Y., Yu, Y., Guo, X., Liu, S., Yu, M., et al. (2021). ALKBH5 regulates cardiomyocyte proliferation and heart regeneration by demethylating the mRNA of YTHDF1. Theranostics *11*, 3000–3016. https://doi.org/10.7150/thno.47354.

55. Yang, C., Zhao, K., Zhang, J., Wu, X., Sun, W., Kong, X., and Shi, J. (2021). Comprehensive analysis of the transcriptome-wide m6A methylome of heart via MeRIP after birth: day 0 vs. Day 7. Front. Cardiovasc. Med. *8*, 633631. https://doi.org/10.3389/fcvm.2021.633631.

56. Chamorro-Jorganes, A., Sweaad, W.K., Katare, R., Besnier, M., Anwar, M., Beazley-Long, N., Sala-Newby, G., Ruiz-Polo, I., Chandrasekera, D., Ritchie, A.A., et al. (2021). METTL3 regulates angiogenesis by modulating let-7e-5p and miRNA-18a-5p expression in endothelial cells. Arterioscler. Thromb. Vasc. Biol. *41*, e325–e337. https://doi.org/10.1161/ATVBAHA.121.316180.

57. Yao, M.D., Jiang, Q., Ma, Y., Liu, C., Zhu, C.Y., Sun, Y.N., Shan, K., Ge, H.M., Zhang, Q.Y., Zhang, H.Y., et al. (2020). Role of METTL3-dependent N(6)-methyladenosine mRNA modification in the promotion of angiogenesis. Mol. Ther. *28*, 2191–2202. https://doi.org/10.1016/j.ymthe.2020.07.022.

58. Shan, K., Zhou, R.M., Xiang, J., Sun, Y.N., Liu, C., Lv, M.W., and Xu, J.J. (2020). FTO regulates ocular angiogenesis via m(6)A-YTHDF2-dependent mechanism. Exp. Eye Res. *197*, 108107. https://doi.org/10.1016/j.exer.2020.108107.

59. Lin, J., Zhu, Q., Huang, J., Cai, R., and Kuang, Y. (2020). Hypoxia promotes vascular smooth muscle cell (VSMC) differentiation of adipose-derived stem cell (ADSC) by regulating Mettl3 and paracrine factors. Stem Cells Int. *2020*, 2830565. https://doi.org/10.1155/2020/2830565.

60. Li, T., Zhuang, Y., Yang, W., Xie, Y., Shang, W., Su, S., Dong, X., Wu, J., Jiang, W., Zhou, Y., et al. (2021). Silencing of METTL3 attenuates cardiac fibrosis induced by myocardial infarction via inhibiting the activation of cardiac fibroblasts. FASEB J. *35*, e21162. https://doi.org/10.1096/fj.201903169R.

61. Geula, S., Moshitch-Moshkovitz, S., Dominissini, D., Mansour, A.A., Kol, N., Salmon-Divon, M., Hershkovitz, V., Peer, E., Mor, N., Manor, Y.S., et al. (2015). Stem cells. m6A mRNA methylation facilitates resolution of naive pluripotency toward differentiation. Science *347*, 1002–1006. https://doi.org/10.1126/science.1261417.

62. Jiang, X., Liu, B., Nie, Z., Duan, L., Xiong, Q., Jin, Z., Yang, C., and Chen, Y. (2021). The role of m6A modification in the biological functions and diseases. Signal Transduct. Target. Ther. *6*, 74. https://doi.org/10.1038/s41392-020-00450-x.

63. Yang, C., Hu, Y., Zhou, B., Bao, Y., Li, Z., Gong, C., Yang, H., Wang, S., and Xiao, Y. (2020). The role of m(6)A modification in physiology and disease. Cell Death Dis. *11*, 960. https://doi.org/10.1038/s41419-020-03143-z.

64. Wilkinson, E., Cui, Y.H., and He, Y.Y. (2021). Context-dependent roles of RNA modifications in stress responses and diseases. Int. J. Mol. Sci. *22*, 1949. https://doi.org/10.3390/ijms22041949.

65. van der Kwast, R.V.C.T., van Ingen, E., Parma, L., Peters, H.A.B., Quax, P.H.A., and Nossent, A.Y. (2018). Adenosine-to-Inosine editing of MicroRNA-487b alters target gene selection after ischemia and promotes neovascularization. Circ. Res. *122*, 444–456. https://doi.org/10.1161/CIRCRESAHA.117.312345.

66. van der Kwast, R.V.C.T., Woudenberg, T., Quax, P.H.A., and Nossent, A.Y. (2020). MicroRNA-411 and its 5'-IsomiR have distinct targets and functions and are differentially regulated in the vasculature under ischemia. Mol. Ther. *28*, 157–170. https://doi.org/10.1016/j.ymthe.2019.10.002.

67. van der Kwast, R.V.C.T., Parma, L., van der Bent, M.L., van Ingen, E., Baganha, F., Peters, H.A.B., Goossens, E.A.C., Simons, K.H., Palmen, M., de Vries, M.R., et al. (2020). Adenosine-to-Inosine editing of vasoactive MicroRNAs alters their targetome and function in ischemia. Mol. Ther. Nucleic Acids *21*, 932–953. https://doi.org/10.1016/j.omtn.2020.07.020.

68. van der Kwast, R., Quax, P.H.A., and Nossent, A.Y. (2019). An emerging role for isomiRs and the microRNA epitranscriptome in neovascularization. Cells *9*, 61. https://doi.org/10.3390/cells9010061.

69. Hartner, J.C., Walkley, C.R., Lu, J., and Orkin, S.H. (2009). ADAR1 is essential for the maintenance of hematopoiesis and suppression of interferon signaling. Nat. Immunol. *10*, 109–115. https://doi.org/10.1038/ni.1680.

70. Marceca, G.P., Tomasello, L., Distefano, R., Acunzo, M., Croce, C.M., and Nigita, G. (2021). Detecting and characterizing A-to-I microRNA editing in cancer. Cancers *13*, 1699. https://doi.org/10.3390/cancers13071699.

71. Shafik, A.M., Allen, E.G., and Jin, P. (2022). Epitranscriptomic dynamics in brain development and disease. Mol. Psychiatry. https://doi.org/10.1038/s41380-022-01570-2.

72. Quiles-Jiménez, A., Gregersen, I., Mittelstedt Leal de Sousa, M., Abbas, A., Kong, X.Y., Alseth, I., Holm, S., Dahl, T.B., Skagen, K., Skjelland, M., et al. (2020). N6-methyladenosine in RNA of atherosclerotic plaques: an epitranscriptomic signature of human carotid atherosclerosis. Biochem. Biophys. Res. Commun. *533*, 631–637. https://doi.org/10.1016/j.bbrc.2020.09.057.

73. Kong, X.Y., Huse, C., Yang, K., Øgaard, J., Berges, N., Vik, E.S., Nawaz, M.S., Quiles-Jiménez, A., Abbas, A., Gregersen, I., et al. (2021). Endonuclease V regulates atherosclerosis through C-C motif chemokine ligand 2-mediated monocyte infiltration. J. Am. Heart Assoc. *10*, e020656. https://doi.org/10.1161/JAHA.120.020656.

74. Su, H., Wang, G., Wu, L., Ma, X., Ying, K., and Zhang, R. (2020). Transcriptome-wide map of m(6)A circRNAs identified in a rat model of hypoxia mediated pulmonary hypertension. BMC Genom. *21*, 39. https://doi.org/10.1186/s12864-020-6462-y.

75. Qin, Y., Qiao, Y., Li, L., Luo, E., Wang, D., Yao, Y., Tang, C., and Yan, G. (2021). The m(6)A methyltransferase METTL3 promotes hypoxic pulmonary arterial hypertension. Life Sci. *274*, 119366. https://doi.org/10.1016/j.lfs.2021.119366.

76. Zhou, X.L., Huang, F.J., Li, Y., Huang, H., and Wu, Q.C. (2021). SEDT2/METTL14-mediated m6A methylation awakening contributes to hypoxia-induced pulmonary arterial hypertension in mice. Aging (Albany NY) *13*, 7538–7548. https://doi.org/10.18632/aging.202616.

77. Xu, S., Xu, X., Zhang, Z., Yan, L., Zhang, L., and Du, L. (2021). The role of RNA m(6)A methylation in the regulation of postnatal hypoxia-induced pulmonary hypertension. Respir. Res. *22*, 121. https://doi.org/10.1186/s12931-021-01728-6.

78. Zeng, Y., Huang, T., Zuo, W., Wang, D., Xie, Y., Wang, X., Xiao, Z., Chen, Z., Liu, Q., Liu, N., et al. (2021). Integrated analysis of m(6)A mRNA methylation in rats with monocrotaline-induced pulmonary arterial hypertension. Aging (Albany NY) *13*, 18238–18256. https://doi.org/10.18632/aging.203230.

79. Xin, W., He, S., Du, Y., Yu, Y., Song, X., zhang, j., Jiang, Y., Li, S., Zhang, J., and zhu, d. (2021). WTAP-mediated GPX4 m6A methylation triggers PASMCs ferroptosis and pulmonary vascular fibrosis in pulmonary artery hypertension. Authorea. https://doi.org/10.22541/au.162872052.24960786/v1.

80. Hu, L., Wang, J., Huang, H., Yu, Y., Ding, J., Yu, Y., Li, K., Wei, D., Ye, Q., Wang, F., et al. (2021). YTHDF1 regulates pulmonary hypertension through translational control of MAGED1. Am. J. Respir. Crit. Care Med. *203*, 1158–1172. https://doi.org/10.1164/rccm.202009-3419OC.

81. Guo, X., Lin, Y., Lin, Y., Zhong, Y., Yu, H., Huang, Y., Yang, J., Cai, Y., Liu, F., Li, Y., et al. (2022). PM2.5 induces pulmonary microvascular injury in COPD via METTL16-mediated m6A modification. Environ. Pollut. *303*, 119115. https://doi.org/10.1016/j.envpol.2022.119115.

82. He, Y., Xing, J., Wang, S., Xin, S., Han, Y., and Zhang, J. (2019). Increased m6A methylation level is associated with the progression of human abdominal aortic aneurysm. Ann. Transl. Med. *7*, 797. https://doi.org/10.21037/atm.2019.12.65.

83. Zhou, X., Chen, Z., Zhou, J., Liu, Y., Fan, R., and Sun, T. (2021). Transcriptome and N6-methyladenosine RNA methylome analyses in aortic dissection and normal human aorta. Front. Cardiovasc. Med. *8*, 627380. https://doi.org/10.3389/fcvm.2021.627380.

84. Li, T., Wang, T., Jing, J., and Sun, L. (2021). Expression pattern and clinical value of key m6A RNA modification regulators in abdominal aortic aneurysm. J. Inflamm. Res. *14*, 4245–4258. https://doi.org/10.2147/JIR.S327152.

85. Chai, T., Tian, M., Yang, X., Qiu, Z., Lin, X., and Chen, L. (2021). Genome-wide identification of RNA modifications for spontaneous coronary aortic dissection. Front. Genet. *12*, 696562. https://doi.org/10.3389/fgene.2021.696562.

86. Zhong, L., He, X., Song, H., Sun, Y., Chen, G., Si, X., Sun, J., Chen, X., Liao, W., Liao, Y., et al. (2020). METTL3 induces AAA development and progression by modulating N6-methyladenosine-dependent primary miR34a processing. Mol. Ther. Nucleic Acids *21*, 394–411. https://doi.org/10.1016/j.omtn.2020.06.005.

87. Ma, D., Liu, X., Zhang, J.J., Zhao, J.J., Xiong, Y.J., Chang, Q., Wang, H.Y., Su, P., Meng, J., and Zhao, Y.B. (2020). Vascular smooth muscle FTO promotes aortic dissecting aneurysms via m6A modification of Klf5. Front. Cardiovasc. Med. *7*, 592550. https://doi.org/10.3389/fcvm.2020.592550.

88. Wang, P., Wang, Z., Zhang, M., Wu, Q., Shi, F., and Yuan, S. (2021). KIAA1429 and ALKBH5 oppositely influence aortic dissection progression via regulating the maturation of Pri-miR-143-3p in an m6A-dependent manner. Front. Cell Dev. Biol. *9*, 668377. https://doi.org/10.3389/fcell.2021.668377.

89. Mo, X.B., Lei, S.F., Zhang, Y.H., and Zhang, H. (2018). Detection of m(6)A-associated SNPs as potential functional variants for coronary artery disease. Epigenomics *10*, 1279–1287. https://doi.org/10.2217/epi-2018-0007.

90. Jian, D., Wang, Y., Jian, L., Tang, H., Rao, L., Chen, K., Jia, Z., Zhang, W., Liu, Y., Chen, X., et al. (2020). METTL14 aggravates endothelial inflammation and atherosclerosis by increasing FOXO1 N6-methyladeosine modifications. Theranostics *10*, 8939–8956. https://doi.org/10.7150/thno.45178.

91. Zhang, B.Y., Han, L., Tang, Y.F., Zhang, G.X., Fan, X.L., Zhang, J.J., Xue, Q., and Xu, Z.Y. (2020). METTL14 regulates M6A methylation-modified primary miR-19a to promote cardiovascular endothelial cell proliferation and invasion. Eur. Rev. Med. Pharmacol. Sci. *24*, 7015–7023. https://doi.org/10.26355/eurrev_202006_21694.

92. Chien, C.S., Li, J.Y.S., Chien, Y., Wang, M.L., Yarmishyn, A.A., Tsai, P.H., Juan, C.C., Nguyen, P., Cheng, H.M., Huo, T.I., et al. (2021). METTL3-dependent N(6)-methyladenosine RNA modification mediates the atherogenic inflammatory cascades in vascular endothelium. Proc. Natl. Acad. Sci. USA *118*. e2025070118. https://doi.org/10.1073/pnas.2025070118.

93. Gong, C., Fan, Y., and Liu, J. (2021). METTL14 mediated m6A modification to LncRNA ZFAS1/RAB22A: a novel therapeutic target for atherosclerosis. Int. J. Cardiol. *328*, 177. https://doi.org/10.1016/j.ijcard.2020.12.002.

94. Mo, C., Yang, M., Han, X., Li, J., Gao, G., Tai, H., Huang, N., and Xiao, H. (2017). Fat mass and obesity-associated protein attenuates lipid accumulation in macrophage foam cells and alleviates atherosclerosis in apolipoprotein E-deficient mice. J. Hypertens. *35*, 810–821. https://doi.org/10.1097/HJH.0000000000001255.

95. Li, B., Zhang, T., Liu, M., Cui, Z., Zhang, Y., Liu, M., Liu, Y., Sun, Y., Li, M., Tian, Y., et al. (2022). RNA N(6)-methyladenosine modulates endothelial atherogenic responses to disturbed flow in mice. Elife *11*, e69906. https://doi.org/10.7554/eLife.69906.

96. Yu, Z., Zheng, X., Wang, C., Chen, C., Ning, N., Peng, D., Liu, T., and Pan, W. (2022). The traditional Chinese medicine Hua tuo Zai Zao wan alleviates atherosclerosis by deactivation of inflammatory macrophages. Evid. Based. Complement. Alternat. Med. *2022*, 2200662. https://doi.org/10.1155/2022/2200662.

97. Dong, G., Yu, J., Shan, G., Su, L., Yu, N., and Yang, S. (2021). N6-Methyladenosine methyltransferase METTL3 promotes angiogenesis and atherosclerosis by upregulating the JAK2/STAT3 pathway via m6A reader IGF2BP1. Front. Cell Dev. Biol. *9*, 731810. https://doi.org/10.3389/fcell.2021.731810.

98. Li, Z., Xu, Q., Huangfu, N., Chen, X., and Zhu, J. (2022). Mettl3 promotes oxLDL-mediated inflammation through activating STAT1 signaling. J. Clin. Lab. Anal. *36*, e24019. https://doi.org/10.1002/jcla.24019.

99. Guo, M., Yan, R., Ji, Q., Yao, H., Sun, M., Duan, L., Xue, Z., and Jia, Y. (2020). IFN regulatory Factor-1 induced macrophage pyroptosis by modulating m6A modification of circ_0029589 in patients with acute coronary syndrome. Int. Immunopharmacol. *86*, 106800. https://doi.org/10.1016/j.intimp.2020.106800.

100. Zhang, X., Li, X., Jia, H., An, G., and Ni, J. (2021). The m(6)A methyltransferase METTL3 modifies PGC-1alpha mRNA promoting mitochondrial dysfunction and oxLDL-induced inflammation in monocytes. J. Biol. Chem. *297*, 101058. https://doi.org/10.1016/j.jbc.2021.101058.

101. Chen, J., Ning, Y., Zhang, H., Song, N., Gu, Y., Shi, Y., Cai, J., Ding, X., and Zhang, X. (2019). METTL14-dependent m6A regulates vascular calcification induced by indoxyl sulfate. Life Sci. *239*, 117034. https://doi.org/10.1016/j.lfs.2019.117034.

102. Li, J., Zhu, Z., and Shu, C. (2017). Treatment with oxLDL antibody reduces cathepsin S expression in atherosclerosis via down-regulating ADAR1-mediated RNA editing. Int. J. Cardiol. *229*, 7. https://doi.org/10.1016/j.ijcard.2016.11.313.

103. Vlachogiannis, N.I., Sachse, M., Georgiopoulos, G., Zormpas, E., Bampatsias, D., Delialis, D., Bonini, F., Galyfos, G., Sigala, F., Stamatelopoulos, K., et al. (2021). Adenosine-to-inosine Alu RNA editing controls the stability of the pro-inflammatory long noncoding RNA NEAT1 in atherosclerotic cardiovascular disease. J. Mol. Cell. Cardiol. *160*, 111–120. https://doi.org/10.1016/j.yjmcc.2021.07.005.

104. Zhao, Y., Hu, J., Sun, X., Yang, K., Yang, L., Kong, L., Zhang, B., Li, F., Li, C., Shi, B., et al. (2021). Loss of m6A demethylase ALKBH5 promotes post-ischemic angiogenesis via post-transcriptional stabilization of WNT5A. Clin. Transl. Med. *11*, e402. https://doi.org/10.1002/ctm2.402.

105. Wang, L.J., Xue, Y., Li, H., Huo, R., Yan, Z., Wang, J., Xu, H., Wang, J., Cao, Y., and Zhao, J.Z. (2020). Wilms' tumour 1-associating protein inhibits endothelial cell angiogenesis by m6A-dependent epigenetic silencing of desmoplakin in brain arteriovenous malformation. J. Cell Mol. Med. *24*, 4981–4991. https://doi.org/10.1111/jcmm.15101.

106. Wang, L.J., Xue, Y., Huo, R., Yan, Z., Xu, H., Li, H., Wang, J., Zhang, Q., Cao, Y., and Zhao, J.Z. (2020). N6-methyladenosine methyltransferase METTL3 affects the phenotype of cerebral arteriovenous malformation via modulating Notch signaling pathway. J. Biomed. Sci. *27*, 62. https://doi.org/10.1186/s12929-020-00655-w.

107. Mathiyalagan, P., Adamiak, M., Mayourian, J., Sassi, Y., Liang, Y., Agarwal, N., Jha, D., Zhang, S., Kohlbrenner, E., Chepurko, E., et al. (2019). FTO-dependent N(6)-methyladenosine regulates cardiac function during remodeling and repair. Circulation *139*, 518–532. https://doi.org/10.1161/CIRCULATIONAHA.118.033794.

108. Jiang, W., Zhu, P., Huang, F., Zhao, Z., Zhang, T., An, X., et al. (2021). The RNA Methyltransferase METTL3 Promotes Endothelial Progenitor Cell Angiogenesis in Mandibular Distraction Osteogenesis via the PI3K/AKT Pathway. Front. Cell Dev. Biol. *9*, 720925. https://doi.org/10.3389/fcell.2021.720925.

109. Kumari, R., Dutta, R., Ranjan, P., Suleiman, Z.G., Goswami, S.K., Li, J., Pal, H.C., and Verma, S.K. (2021). ALKBH5 regulates SPHK1-dependent endothelial cell angiogenesis following ischemic stress. Front. Cardiovasc. Med. *8*, 817304. https://doi.org/10.3389/fcvm.2021.817304.

110. van den Homberg, D.A.L., van der Kwast, R.V.C.T., Quax, P.H.A., and Nossent, A.Y. (2022). N-6-Methyladenosine in vasoactive microRNAs during hypoxia; A novel role for METTL4. Int. J. Mol. Sci. *23*, 1057. https://doi.org/10.3390/ijms23031057.

111. Zhang, Y., Hua, W., Dang, Y., Cheng, Y., Wang, J., Zhang, X., Teng, M., Wang, S., Zhang, M., Kong, Z., et al. (2021). Validated impacts of N6-methyladenosine methylated mRNAs on apoptosis and angiogenesis in myocardial infarction based on MeRIP-seq analysis. Front. Mol. Biosci. *8*, 789923. https://doi.org/10.3389/fmolb.2021.789923.

112. Zheng, Y., Nie, P., Peng, D., He, Z., Liu, M., Xie, Y., Miao, Y., Zuo, Z., and Ren, J. (2018). m6Avar: a database of functional variants involved in m6A modification. Nucleic Acids Res. *46*, D139–D145. https://doi.org/10.1093/nar/gkx895.

113. Mo, X.B., Lei, S.F., Zhang, Y.H., and Zhang, H. (2019). Examination of the associations between m(6)A-associated single-nucleotide polymorphisms and blood pressure. Hypertens. Res. *42*, 1582–1589. https://doi.org/10.1038/s41440-019-0277-8.

114. Pausova, Z., Syme, C., Abrahamowicz, M., Xiao, Y., Leonard, G.T., Perron, M., Richer, L., Veillette, S., Smith, G.D., Seda, O., et al. (2009). A common variant of the FTO gene is associated with not only increased adiposity but also elevated blood pressure in French Canadians. Circ. Cardiovasc. Genet. *2*, 260–269. https://doi.org/10.1161/CIRCGENETICS.109.857359.

115. Wu, Q., Yuan, X., Han, R., Zhang, H., and Xiu, R. (2019). Epitranscriptomic mechanisms of N6-methyladenosine methylation regulating mammalian hypertension development by determined spontaneously hypertensive rats pericytes. Epigenomics *11*, 1359–1370. https://doi.org/10.2217/epi-2019-0148.

116. Jain, M., Mann, T.D., Stulić, M., Rao, S.P., Kirsch, A., Pullirsch, D., Strobl, X., Rath, C., Reissig, L., Moreth, K., et al. (2018). RNA editing of Filamin A pre-mRNA regulates vascular contraction and diastolic blood pressure. EMBO J. *37*, e94813. https://doi.org/10.15252/embj.201694813.

117. Krüger, N., Biwer, L.A., Good, M.E., Ruddiman, C.A., Wolpe, A.G., DeLalio, L.J., Murphy, S., Macal, E.H., Jr., Ragolia, L., Serbulea, V., et al. (2020). Loss of endothelial FTO antagonizes obesity-induced metabolic and vascular dysfunction. Circ. Res. *126*, 232–242. https://doi.org/10.1161/CIRCRESAHA.119.315531.

118. Gan, X.T., Zhao, G., Huang, C.X., Rowe, A.C., Purdham, D.M., and Karmazyn, M. (2013). Identification of fat mass and obesity associated (FTO) protein expression in cardiomyocytes: regulation by leptin and its contribution to leptin-induced hypertrophy. PLoS One *8*, e74235. https://doi.org/10.1371/journal.pone.0074235.

119. Carnevali, L., Graiani, G., Rossi, S., Al Banchaabouchi, M., Macchi, E., Quaini, F., Rosenthal, N., and Sgoifo, A. (2014). Signs of cardiac autonomic imbalance and proarrhythmic remodeling in FTO deficient mice. PLoS One *9*, e95499. https://doi.org/10.1371/journal.pone.0095499.

120. Dorn, L.E., Lasman, L., Chen, J., Xu, X., Hund, T.J., Medvedovic, M., Hanna, J.H., van Berlo, J.H., and Accornero, F. (2019). The N(6)-methyladenosine mRNA Methylase METTL3 controls cardiac homeostasis and hypertrophy. Circulation *139*, 533–545. https://doi.org/10.1161/CIRCULATIONAHA.118.036146.

121. Kmietczyk, V., Riechert, E., Kalinski, L., Boileau, E., Malovrh, E., Malone, B., Gorska, A., Hofmann, C., Varma, E., Jürgensen, L., et al. (2019). m(6)A-mRNA methylation regulates cardiac gene expression and cellular growth. Life Sci. Alliance *2*, e201800233. https://doi.org/10.26508/lsa.201800233.

122. Berulava, T., Buchholz, E., Elerdashvili, V., Pena, T., Islam, M.R., Lbik, D., Mohamed, B.A., Renner, A., von Lewinski, D., Sacherer, M., et al. (2020).

Changes in m6A RNA methylation contribute to heart failure progression by modulating translation. Eur. J. Heart Fail. *22*, 54–66. https://doi.org/10.1002/ejhf.1672.

123. Gao, X.Q., Zhang, Y.H., Liu, F., Ponnusamy, M., Zhao, X.M., Zhou, L.Y., Zhai, M., Liu, C.Y., Li, X.M., Wang, M., et al. (2020). The piRNA CHAPIR regulates cardiac hypertrophy by controlling METTL3-dependent N(6)-methyladenosine methylation of Parp10 mRNA. Nat. Cell Biol. *22*, 1319–1331. https://doi.org/10.1038/s41556-020-0576-y.

124. Qian, B., Wang, P., Zhang, D., and Wu, L. (2021). m6A modification promotes miR-133a repression during cardiac development and hypertrophy via IGF2BP2. Cell Death Discov. *7*, 157. https://doi.org/10.1038/s41420-021-00552-7.

125. Xu, H., Wang, Z., Chen, M., Zhao, W., Tao, T., Ma, L., Ni, Y., and Li, W. (2021). YTHDF2 alleviates cardiac hypertrophy via regulating Myh7 mRNA decoy. Cell Biosci. *11*, 132. https://doi.org/10.1186/s13578-021-00649-7.

126. Lu, P., Xu, Y., Sheng, Z.Y., Peng, X.G., Zhang, J.J., Wu, Q.H., Wu, Y.Q., Cheng, X.S., and Zhu, K. (2021). De-ubiquitination of p300 by USP12 critically enhances METTL3 expression and ang II-induced cardiac hypertrophy. Exp. Cell Res. *406*, 112761. https://doi.org/10.1016/j.yexcr.2021.112761.

127. Ju, W., Liu, K., Ouyang, S., Liu, Z., He, F., and Wu, J. (2021). Changes in N6-methyladenosine modification modulate diabetic cardiomyopathy by reducing myocardial fibrosis and myocyte hypertrophy. Front. Cell Dev. Biol. *9*, 702579. https://doi.org/10.3389/fcell.2021.702579.

128. Fang, M., Deng, J., Zhou, Q., Hu, Z., and Yang, L. (2022). Maslinic acid protects against pressure-overload-induced cardiac hypertrophy by blocking METTL3-mediated m(6)A methylation. Aging (Albany NY) *14*, 2548–2557. https://doi.org/10.18632/aging.203860.

129. Yang, Y., Mbikyo, M.B., Zhang, J., Zhang, Y., Zhang, N., and Li, Z. (2022). The lncRNA MIAT regulates CPT-1a mediated cardiac hypertrophy through m(6)A RNA methylation reading protein Ythdf2. Cell Death Discov. *8*, 167. https://doi.org/10.1038/s41420-022-00977-8.

130. El Azzouzi, H., Vilaça, A.P., Feyen, D.A.M., Gommans, W.M., de Weger, R.A., Doevendans, P.A.F., and Sluijter, J.P.G. (2020). Cardiomyocyte specific deletion of ADAR1 causes severe cardiac dysfunction and increased lethality. Front. Cardiovasc. Med. *7*, 30. https://doi.org/10.3389/fcvm.2020.00030.

131. Hinger, S.A., Wei, J., Dorn, L.E., Whitson, B.A., Janssen, P.M.L., He, C., and Accornero, F. (2021). Remodeling of the m(6)A landscape in the heart reveals few conserved post-transcriptional events underlying cardiomyocyte hypertrophy. J. Mol. Cell. Cardiol. *151*, 46–55. https://doi.org/10.1016/j.yjmcc.2020.11.002.

132. Altaf, F., Vesely, C., Sheikh, A.M., Munir, R., Shah, S.T.A., and Tariq, A. (2019). Modulation of ADAR mRNA expression in patients with congenital heart defects. PLoS One *14*, e0200968. https://doi.org/10.1371/journal.pone.0200968.

133. Shi, X., Cao, Y., Zhang, X., Gu, C., Liang, F., Xue, J., Ni, H.W., Wang, Z., Li, Y., Wang, X., et al. (2021). Comprehensive analysis of N6-methyladenosine RNA methylation regulators expression identify distinct molecular subtypes of myocardial infarction. Front. Cell Dev. Biol. *9*, 756483. https://doi.org/10.3389/fcell.2021.756483.

134. Song, H., Feng, X., Zhang, H., Luo, Y., Huang, J., Lin, M., Jin, J., Ding, X., Wu, S., Huang, H., et al. (2019). METTL3 and ALKBH5 oppositely regulate m(6)A modification of TFEB mRNA, which dictates the fate of hypoxia/reoxygenation-treated cardiomyocytes. Autophagy *15*, 1419–1437. https://doi.org/10.1080/15548627.2019.1586246.

135. Shen, W., Li, H., Su, H., Chen, K., and Yan, J. (2021). FTO overexpression inhibits apoptosis of hypoxia/reoxygenation-treated myocardial cells by regulating m6A modification of Mhrt. Mol. Cell. Biochem. *476*, 2171–2179. https://doi.org/10.1007/s11010-021-04069-6.

136. Wang, J., Zhang, J., Ma, Y., Zeng, Y., Lu, C., Yang, F., Jiang, N., Zhang, X., Wang, Y., Xu, Y., et al. (2021). WTAP promotes myocardial ischemia/reperfusion injury by increasing endoplasmic reticulum stress via regulating m(6)A modification of ATF4 mRNA. Aging (Albany NY) *13*, 11135–11149. https://doi.org/10.18632/aging.202770.

137. Su, Y., Xu, R., Zhang, R., Qu, Y., Zuo, W., Ji, Z., Geng, H., Pan, M., and Ma, G. (2020). N6-methyladenosine methyltransferase plays a role in hypoxic preconditioning partially through the interaction with lncRNA H19. Acta Biochim. Biophys. Sin. *52*, 1306–1315. https://doi.org/10.1093/abbs/gmaa130.

138. Su, X., Shen, Y., Jin, Y., Kim, I.M., Weintraub, N.L., and Tang, Y. (2021). Aging-associated differences in epitranscriptomic m6A regulation in response to acute cardiac ischemia/reperfusion injury in female mice. Front. Pharmacol. *12*, 654316. https://doi.org/10.3389/fphar.2021.654316.

139. Chang, J.S., Lin, Z.X., Liu, Y.J., Yang, S.M., Zhang, Y., and Yu, X.Y. (2021). Ultra performance liquid chromatography-tandem mass spectrometry assay for the quantification of RNA and DNA methylation. J. Pharm. Biomed. Anal. *197*, 113969. https://doi.org/10.1016/j.jpba.2021.113969.

140. Gong, R., Wang, X., Li, H., Liu, S., Jiang, Z., Zhao, Y., Yu, Y., Han, Z., Yu, Y., Dong, C., et al. (2021). Loss of m(6)A methyltransferase METTL3 promotes heart regeneration and repair after myocardial injury. Pharmacol. Res. *174*, 105845. https://doi.org/10.1016/j.phrs.2021.105845.

141. Zhang, M., Chen, Y., Chen, H., Shen, Y., Pang, L., Wu, W., and Yu, Z. (2022). Tanshinone IIA alleviates cardiac hypertrophy through m6A modification of galectin-3. Bioengineered *13*, 4260–4270. https://doi.org/10.1080/21655979.2022.2031388.

142. Wang, X., Li, Y., Li, J., Li, S., and Wang, F. (2022). Mechanism of METTL3-mediated m(6)A modification in cardiomyocyte pyroptosis and myocardial ischemia-reperfusion injury. Cardiovasc. Drugs Ther. https://doi.org/10.1007/s10557-021-07300-0.

143. Pang, P., Qu, Z., Yu, S., Pang, X., Li, X., Gao, Y., Liu, K., Liu, Q., Wang, X., Bian, Y., et al. (2021). Mettl14 attenuates cardiac ischemia/reperfusion injury by regulating Wnt1/beta-catenin signaling pathway. Front. Cell Dev. Biol. *9*, 762853. https://doi.org/10.3389/fcell.2021.762853.

144. Ye, F., Wang, X., Tu, S., Zeng, L., Deng, X., Luo, W., and Zhang, Z. (2021). The effects of NCBP3 on METTL3-mediated m6A RNA methylation to enhance translation process in hypoxic cardiomyocytes. J. Cell Mol. Med. *25*, 8920–8928. https://doi.org/10.1111/jcmm.16852.

145. Ke, W.L., Huang, Z.W., Peng, C.L., and Ke, Y.P. (2022). m(6)A demethylase FTO regulates the apoptosis and inflammation of cardiomyocytes via YAP1 in ischemia-reperfusion injury. Bioengineered *13*, 5443–5452. https://doi.org/10.1080/21655979.2022.2030572.

146. Wu, X., Wang, L., Wang, K., Li, J., Chen, R., Wu, X., Ni, G., Liu, C., Das, S., Sluijter, J.P.G., et al. (2022). ADAR2 increases in exercised heart and protects against myocardial infarction and doxorubicin-induced cardiotoxicity. Mol. Ther. *30*, 400–414. https://doi.org/10.1016/j.ymthe.2021.07.004.

147. Zhao, K., Yang, C., Zhang, J., Sun, W., Zhou, B., Kong, X., and Shi, J. (2021). METTL3 improves cardiomyocyte proliferation upon myocardial infarction via upregulating miR-17-3p in a DGCR8-dependent manner. Cell Death Discov. *7*, 291. https://doi.org/10.1038/s41420-021-00688-6.

148. Sun, P., Wang, C., Mang, G., Xu, X., Fu, S., Chen, J., Wang, X., Wang, W., Li, H., Zhao, P., et al. (2022). Extracellular vesicle-packaged mitochondrial disturbing miRNA exacerbates cardiac injury during acute myocardial infarction. Clin. Transl. Med. *12*, e779. https://doi.org/10.1002/ctm2.779.

149. Li, X.X., Mu, B., Li, X., and Bie, Z.D. (2022). circCELF1 inhibits myocardial fibrosis by regulating the expression of DKK2 through FTO/m(6)A and miR-636. J. Cardiovasc. Transl. Res. https://doi.org/10.1007/s12265-022-10209-0.

150. Gao, S., Sun, H., Chen, K., Gu, X., Chen, H., Jiang, L., Chen, L., Zhang, S., Liu, Y., Shi, D., et al. (2021). Depletion of m(6) A reader protein YTHDC1 induces dilated cardiomyopathy by abnormal splicing of Titin. J. Cell Mol. Med. *25*, 10879–10891. https://doi.org/10.1111/jcmm.16955.

151. Zhang, B., Xu, Y., Cui, X., Jiang, H., Luo, W., Weng, X., Wang, Y., Zhao, Y., Sun, A., and Ge, J. (2021). Alteration of m6A RNA methylation in heart failure with preserved ejection fraction. Front. Cardiovasc. Med. *8*, 647806. https://doi.org/10.3389/fcvm.2021.647806.

152. Meng, L., Lin, H., Huang, X., Weng, J., Peng, F., and Wu, S. (2022). METTL14 suppresses pyroptosis and diabetic cardiomyopathy by downregulating TINCR lncRNA. Cell Death Dis. *13*, 38. https://doi.org/10.1038/s41419-021-04484-z.

153. Shao, Y., Li, M., Yu, Q., Gong, M., Wang, Y., Yang, X., Liu, L., Liu, D., Tan, Z., Zhang, Y., et al. (2022). CircRNA CDR1as promotes cardiomyocyte apoptosis through activating hippo signaling pathway in diabetic cardiomyopathy. Eur. J. Pharmacol. *922*, 174915. https://doi.org/10.1016/j.ejphar.2022.174915.

154. Yu, Y., Pan, Y., Fan, Z., Xu, S., Gao, Z., Ren, Z., Yu, J., Li, W., Liu, F., Gu, J., et al. (2021). LuHui derivative, A novel compound that inhibits the fat mass and

obesity-associated (FTO), alleviates the inflammatory response and injury in hyperlipidemia-induced cardiomyopathy. Front. Cell Dev. Biol. *9*, 731365. https://doi.org/10.3389/fcell.2021.731365.

155. Xu, Z., Qin, Y., Lv, B., Tian, Z., and Zhang, B. (2022). Intermittent fasting improves high-fat diet-induced obesity cardiomyopathy via alleviating lipid deposition and apoptosis and decreasing m6A methylation in the heart. Nutrients *14*, 251. https://doi.org/10.3390/nu14020251.

156. Crow, Y., Keshavan, N., Barbet, J.P., Bercu, G., Bondet, V., Boussard, C., Dedieu, N., Duffy, D., Hully, M., Giardini, A., et al. (2020). Cardiac valve involvement in ADAR-related type I interferonopathy. J. Med. Genet. *57*, 475–478. https://doi.org/10.1136/jmedgenet-2019-106457.

157. Zhou, T., Han, D., Liu, J., Shi, J., Zhu, P., Wang, Y., and Dong, N. (2021). Factors influencing osteogenic differentiation of human aortic valve interstitial cells. J. Thorac. Cardiovasc. Surg. *161*, e163–e185. https://doi.org/10.1016/j.jtcvs.2019.10.039.

158. Borik, S., Simon, A.J., Nevo-Caspi, Y., Mishali, D., Amariglio, N., Rechavi, G., and Paret, G. (2011). Increased RNA editing in children with cyanotic congenital heart disease. Intensive Care Med. *37*, 1664–1671. https://doi.org/10.1007/s00134-011-2296-z.

159. Arcidiacono, O.A., Krejčí, J., and Bártová, E. (2020). The distinct function and localization of METTL3/METTL14 and METTL16 enzymes in cardiomyocytes. Int. J. Mol. Sci. *21*, E8139. https://doi.org/10.3390/ijms21218139.

160. Wang, S., Zhang, J., Wu, X., Lin, X., Liu, X.M., and Zhou, J. (2021). Differential roles of YTHDF1 and YTHDF3 in embryonic stem cell-derived cardiomyocyte differentiation. RNA Biol. *18*, 1354–1363. https://doi.org/10.1080/15476286.2020.1850628.

161. Moore, J.B., 4th, Sadri, G., Fischer, A.G., Weirick, T., Militello, G., Wysoczynski, M., Gumpert, A.M., Braun, T., and Uchida, S. (2020). The A-to-I RNA editing enzyme Adar1 is essential for normal embryonic cardiac growth and development. Circ. Res. *127*, 550–552. https://doi.org/10.1161/CIRCRESAHA.120.316932.

162. Horsch, M., Seeburg, P.H., Adler, T., Aguilar-Pimentel, J.A., Becker, L., Calzada-Wack, J., Garrett, L., Götz, A., Hans, W., Higuchi, M., et al. (2011). Requirement of the RNA-editing enzyme ADAR2 for normal physiology in mice. J. Biol. Chem. *286*, 18614–18622. https://doi.org/10.1074/jbc.M110.200881.

163. Wang, Q., Miyakoda, M., Yang, W., Khillan, J., Stachura, D.L., Weiss, M.J., and Nishikura, K. (2004). Stress-induced apoptosis associated with null mutation of ADAR1 RNA editing deaminase gene. J. Biol. Chem. *279*, 4952–4961. https://doi.org/10.1074/jbc.M310162200.

164. Witman, N.M., Behm, M., Ohman, M., and Morrison, J.I. (2013). ADAR-related activation of adenosine-to-inosine RNA editing during regeneration. Stem Cells Dev. *22*, 2254–2267. https://doi.org/10.1089/scd.2013.0104.

165. Yang, Y., Shen, S., Cai, Y., Zeng, K., Liu, K., Li, S., Zeng, L., Chen, L., Tang, J., Hu, Z., et al. (2021). Dynamic patterns of N6-methyladenosine profiles of messenger RNA correlated with the cardiomyocyte regenerability during the early heart development in mice. Oxid. Med. Cell. Longev. *2021*, 5537804. https://doi.org/10.1155/2021/5537804.

166. Wang, Z., Cui, M., Shah, A.M., Ye, W., Tan, W., Min, Y.L., Botten, G.A., Shelton, J.M., Liu, N., Bassel-Duby, R., et al. (2019). Mechanistic basis of neonatal heart regeneration revealed by transcriptome and histone modification profiling. Proc. Natl. Acad. Sci. USA *116*, 18455–18465. https://doi.org/10.1073/pnas.1905824116.

167. Yamada, S., Samtani, R.R., Lee, E.S., Lockett, E., Uwabe, C., Shiota, K., Anderson, S.A., and Lo, C.W. (2010). Developmental atlas of the early first trimester human embryo. Dev. Dyn. *239*, 1585–1595. https://doi.org/10.1002/dvdy.22316.

168. Wang, Q., Khillan, J., Gadue, P., and Nishikura, K. (2000). Requirement of the RNA editing deaminase ADAR1 gene for embryonic erythropoiesis. Science *290*, 1765–1768. https://doi.org/10.1126/science.290.5497.1765.

169. Ward, S.V., George, C.X., Welch, M.J., Liou, L.Y., Hahm, B., Lewicki, H., de la Torre, J.C., Samuel, C.E., and Oldstone, M.B. (2011). RNA editing enzyme adenosine deaminase is a restriction factor for controlling measles virus replication that also is required for embryogenesis. Proc. Natl. Acad. Sci. USA *108*, 331–336. https://doi.org/10.1073/pnas.1017241108.

170. Hartner, J.C., Schmittwolf, C., Kispert, A., Müller, A.M., Higuchi, M., and Seeburg, P.H. (2004). Liver disintegration in the mouse embryo caused by deficiency in the RNA-editing enzyme ADAR1. J. Biol. Chem. *279*, 4894–4902. https://doi.org/10.1074/jbc.M311347200.

171. Shtrichman, R., Germanguz, I., Mandel, R., Ziskind, A., Nahor, I., Safran, M., Osenberg, S., Sherf, O., Rechavi, G., and Itskovitz-Eldor, J. (2012). Altered A-to-I RNA editing in human embryogenesis. PLoS One *7*, e41576. https://doi.org/10.1371/journal.pone.0041576.

172. Liddicoat, B.J., Piskol, R., Chalk, A.M., Ramaswami, G., Higuchi, M., Hartner, J.C., Li, J.B., Seeburg, P.H., and Walkley, C.R. (2015). RNA editing by ADAR1 prevents MDA5 sensing of endogenous dsRNA as nonself. Science *349*, 1115–1120. https://doi.org/10.1126/science.aac7049.

173. Mannion, N.M., Greenwood, S.M., Young, R., Cox, S., Brindle, J., Read, D., Nellåker, C., Vesely, C., Ponting, C.P., McLaughlin, P.J., et al. (2014). The RNA-editing enzyme ADAR1 controls innate immune responses to RNA. Cell Rep. *9*, 1482–1494. https://doi.org/10.1016/j.celrep.2014.10.041.

174. Pestal, K., Funk, C.C., Snyder, J.M., Price, N.D., Treuting, P.M., and Stetson, D.B. (2015). Isoforms of RNA-editing enzyme ADAR1 independently control nucleic acid sensor MDA5-driven autoimmunity and multi-organ development. Immunity *43*, 933–944. https://doi.org/10.1016/j.immuni.2015.11.001.

175. Zhang, B., Jiang, H., Wu, J., Cai, Y., Dong, Z., Zhao, Y., Hu, Q., Hu, K., Sun, A., and Ge, J. (2021). m6A demethylase FTO attenuates cardiac dysfunction by regulating glucose uptake and glycolysis in mice with pressure overload-induced heart failure. Signal Transduct. Target. Ther. *6*, 377. https://doi.org/10.1038/s41392-021-00699-w.

176. Wang, X., Xu, L., Gillette, T.G., Jiang, X., and Wang, Z.V. (2018). The unfolded protein response in ischemic heart disease. J. Mol. Cell. Cardiol. *117*, 19–25. https://doi.org/10.1016/j.yjmcc.2018.02.013.

177. Zhou, H., Ren, J., Toan, S., and Mui, D. (2021). Role of mitochondrial quality surveillance in myocardial infarction: from bench to bedside. Ageing Res. Rev. *66*, 101250. https://doi.org/10.1016/j.arr.2020.101250.

178. Tona, F., Montisci, R., Iop, L., and Civieri, G. (2021). Role of coronary microvascular dysfunction in heart failure with preserved ejection fraction. Rev. Cardiovasc. Med. *22*, 97–104. https://doi.org/10.31083/j.rcm.2021.01.277.

179. Gao, G., Xie, A., Zhang, J., Herman, A.M., Jeong, E.M., Gu, L., Liu, M., Yang, K.C., Kamp, T.J., and Dudley, S.C. (2013). Unfolded protein response regulates cardiac sodium current in systolic human heart failure. Circ. Arrhythm. Electrophysiol. *6*, 1018–1024. https://doi.org/10.1161/CIRCEP.113.000274.

180. Ren, J., Bi, Y., Sowers, J.R., Hetz, C., and Zhang, Y. (2021). Endoplasmic reticulum stress and unfolded protein response in cardiovascular diseases. Nat. Rev. Cardiol. *18*, 499–521. https://doi.org/10.1038/s41569-021-00511-w.

181. Sommer, B., Köhler, M., Sprengel, R., and Seeburg, P.H. (1991). RNA editing in brain controls a determinant of ion flow in glutamate-gated channels. Cell *67*, 11–19. https://doi.org/10.1016/0092-8674(91)90568-j.

182. Higuchi, M., Maas, S., Single, F.N., Hartner, J., Rozov, A., Burnashev, N., Feldmeyer, D., Sprengel, R., and Seeburg, P.H. (2000). Point mutation in an AMPA receptor gene rescues lethality in mice deficient in the RNA-editing enzyme ADAR2. Nature *406*, 78–81. https://doi.org/10.1038/35017558.

183. Sakurai, M., Shiromoto, Y., Ota, H., Song, C., Kossenkov, A.V., Wickramasinghe, J., Showe, L.C., Skordalakes, E., Tang, H.Y., Speicher, D.W., et al. (2017). ADAR1 controls apoptosis of stressed cells by inhibiting Staufen1-mediated mRNA decay. Nat. Struct. Mol. Biol. *24*, 534–543. https://doi.org/10.1038/nsmb.3403.

184. Wang, Y., Li, Y., Toth, J.I., Petroski, M.D., Zhang, Z., and Zhao, J.C. (2014). N6-methyladenosine modification destabilizes developmental regulators in embryonic stem cells. Nat. Cell Biol. *16*, 191–198. https://doi.org/10.1038/ncb2902.

185. Gao, Y., Vasic, R., Song, Y., Teng, R., Liu, C., Gbyli, R., Biancon, G., Nelakanti, R., Lobben, K., Kudo, E., et al. (2020). m(6)A modification prevents formation of endogenous double-stranded RNAs and deleterious innate immune responses during hematopoietic development. Immunity *52*, 1007–1021.e8. https://doi.org/10.1016/j.immuni.2020.05.003.

186. Batista, P.J., Molinie, B., Wang, J., Qu, K., Zhang, J., Li, L., Bouley, D.M., Lujan, E., Haddad, B., Daneshvar, K., et al. (2014). m(6)A RNA modification controls cell fate transition in mammalian embryonic stem cells. Cell Stem Cell *15*, 707–719. https://doi.org/10.1016/j.stem.2014.09.019.

187. Shi, H., Zhang, X., Weng, Y.L., Lu, Z., Liu, Y., Lu, Z., Li, J., Hao, P., Zhang, Y., Zhang, F., et al. (2018). m(6)A facilitates hippocampus-dependent learning and memory through YTHDF1. Nature *563*, 249–253. https://doi.org/10.1038/s41586-018-0666-1.

188. Ivanova, I., Much, C., Di Giacomo, M., Azzi, C., Morgan, M., Moreira, P.N., Monahan, J., Carrieri, C., Enright, A.J., and O'Carroll, D. (2017). The RNA m(6)A reader YTHDF2 is essential for the post-transcriptional regulation of the maternal transcriptome and oocyte competence. Mol. Cell *67*, 1059–1067.e4. https://doi.org/10.1016/j.molcel.2017.08.003.

189. Zheng, G., Dahl, J.A., Niu, Y., Fedorcsak, P., Huang, C.M., Li, C.J., Vågbø, C.B., Shi, Y., Wang, W.L., Song, S.H., et al. (2013). ALKBH5 is a mammalian RNA demethylase that impacts RNA metabolism and mouse fertility. Mol. Cell *49*, 18–29. https://doi.org/10.1016/j.molcel.2012.10.015.

190. Porrello, E.R., Mahmoud, A.I., Simpson, E., Hill, J.A., Richardson, J.A., Olson, E.N., and Sadek, H.A. (2011). Transient regenerative potential of the neonatal mouse heart. Science *331*, 1078–1080. https://doi.org/10.1126/science.1200708.

191. Wang, H., Paulsen, M.J., Hironaka, C.E., Shin, H.S., Farry, J.M., Thakore, A.D., Jung, J., Lucian, H.J., Eskandari, A., Anilkumar, S., et al. (2020). Natural heart regeneration in a neonatal rat myocardial infarction model. Cells *9*, 229. https://doi.org/10.3390/cells9010229.

192. von Gise, A., Lin, Z., Schlegelmilch, K., Honor, L.B., Pan, G.M., Buck, J.N., Ma, Q., Ishiwata, T., Zhou, B., Camargo, F.D., et al. (2012). YAP1, the nuclear target of Hippo signaling, stimulates heart growth through cardiomyocyte proliferation but not hypertrophy. Proc. Natl. Acad. Sci. USA *109*, 2394–2399. https://doi.org/10.1073/pnas.1116136109.

193. Lin, Z., Zhou, P., von Gise, A., Gu, F., Ma, Q., Chen, J., Guo, H., van Gorp, P.R.R., Wang, D.Z., and Pu, W.T. (2015). Pi3kcb links Hippo-YAP and PI3K-AKT signaling pathways to promote cardiomyocyte proliferation and survival. Circ. Res. *116*, 35–45. https://doi.org/10.1161/CIRCRESAHA.115.304457.

194. Kielbasa, O.M., Reynolds, J.G., Wu, C.L., Snyder, C.M., Cho, M.Y., Weiler, H., Kandarian, S., and Naya, F.J. (2011). Myospryn is a calcineurin-interacting protein that negatively modulates slow-fiber-type transformation and skeletal muscle regeneration. FASEB J *25*, 2276–2286. https://doi.org/10.1096/fj.10-169219.

195. Scutenaire, J., Deragon, J.M., Jean, V., Benhamed, M., Raynaud, C., Favory, J.J., Merret, R., and Bousquet-Antonelli, C. (2018). The YTH domain protein ECT2 is an m(6)A reader required for normal trichome branching in arabidopsis. Plant Cell *30*, 986–1005. https://doi.org/10.1105/tpc.17.00854.

196. Scarrow, M., Wang, Y., and Sun, G. (2021). Molecular regulatory mechanisms underlying the adaptability of polyploid plants. Biol. Rev. Camb. Philos. Soc. *96*, 394–407. https://doi.org/10.1111/brv.12661.

197. Derks, W., and Bergmann, O. (2020). Polyploidy in cardiomyocytes: roadblock to heart regeneration? Circ. Res. *126*, 552–565. https://doi.org/10.1161/CIRCRESAHA.119.315408.

198. Zhang, S., Chen, Q., Liu, Q., Li, Y., Sun, X., Hong, L., Ji, S., Liu, C., Geng, J., Zhang, W., et al. (2017). Hippo signaling suppresses cell ploidy and tumorigenesis through Skp2. Cancer Cell *31*, 669–684.e7. https://doi.org/10.1016/j.ccell.2017.04.004.

199. Ganem, N.J., Cornils, H., Chiu, S.Y., O'Rourke, K.P., Arnaud, J., Yimlamai, D., Théry, M., Camargo, F.D., and Pellman, D. (2014). Cytokinesis failure triggers hippo tumor suppressor pathway activation. Cell *158*, 833–848. https://doi.org/10.1016/j.cell.2014.06.029.

200. Tamamori-Adachi, M., Takagi, H., Hashimoto, K., Goto, K., Hidaka, T., Koshimizu, U., Yamada, K., Goto, I., Maejima, Y., Isobe, M., et al. (2008). Cardiomyocyte proliferation and protection against post-myocardial infarction heart failure by cyclin D1 and Skp2 ubiquitin ligase. Cardiovasc. Res. *80*, 181–190. https://doi.org/10.1093/cvr/cvn183.

201. Huang, H., Weng, H., Sun, W., Qin, X., Shi, H., Wu, H., Zhao, B.S., Mesquita, A., Liu, C., Yuan, C.L., et al. (2018). Recognition of RNA N(6)-methyladenosine by IGF2BP proteins enhances mRNA stability and translation. Nat. Cell Biol. *20*, 285–295. https://doi.org/10.1038/s41556-018-0045-z.

202. Gillan, L., Matei, D., Fishman, D.A., Gerbin, C.S., Karlan, B.Y., and Chang, D.D. (2002). Periostin secreted by epithelial ovarian carcinoma is a ligand for alpha(V)beta(3) and alpha(V)beta(5) integrins and promotes cell motility. Cancer Res. *62*, 5358–5364.

203. Kühn, B., del Monte, F., Hajjar, R.J., Chang, Y.S., Lebeche, D., Arab, S., and Keating, M.T. (2007). Periostin induces proliferation of differentiated cardiomyocytes and promotes cardiac repair. Nat. Med. *13*, 962–969. https://doi.org/10.1038/nm1619.

204. Xie, Y., Lampinen, M., Takala, J., Sikorski, V., Soliymani, R., Tarkia, M., Lalowski, M., Mervaala, E., Kupari, M., Zheng, Z., et al. (2020). Epicardial transplantation of atrial appendage micrograft patch salvages myocardium after infarction. J. Heart Lung Transplant. *39*, 707–718. https://doi.org/10.1016/j.healun.2020.03.023.

205. Ladage, D., Yaniz-Galende, E., Rapti, K., Ishikawa, K., Tilemann, L., Shapiro, S., Takewa, Y., Muller-Ehmsen, J., Schwarz, M., Garcia, M.J., et al. (2013). Stimulating myocardial regeneration with periostin Peptide in large mammals improves function post-myocardial infarction but increases myocardial fibrosis. PLoS One *8*, e59656. https://doi.org/10.1371/journal.pone.0059656.

206. Oka, T., Xu, J., Kaiser, R.A., Melendez, J., Hambleton, M., Sargent, M.A., Lorts, A., Brunskill, E.W., Dorn, G.W., 2nd, Conway, S.J., et al. (2007). Genetic manipulation of periostin expression reveals a role in cardiac hypertrophy and ventricular remodeling. Circ. Res. *101*, 313–321. https://doi.org/10.1161/CIRCRESAHA.107.149047.

207. Lorts, A., Schwanekamp, J.A., Elrod, J.W., Sargent, M.A., and Molkentin, J.D. (2009). Genetic manipulation of periostin expression in the heart does not affect myocyte content, cell cycle activity, or cardiac repair. Circ. Res. *104*, 1–7. https://doi.org/10.1161/CIRCRESAHA.108.188649.

208. O'Meara, C.C., Wamstad, J.A., Gladstone, R.A., Fomovsky, G.M., Butty, V.L., Shrikumar, A., Gannon, J.B., Boyer, L.A., and Lee, R.T. (2015). Transcriptional reversion of cardiac myocyte fate during mammalian cardiac regeneration. Circ. Res. *116*, 804–815. https://doi.org/10.1161/CIRCRESAHA.116.304269.

209. Li, J., Liu, Y., Jin, Y., Wang, R., Wang, J., Lu, S., VanBuren, V., Dostal, D.E., Zhang, S.L., and Peng, X. (2017). Essential role of Cdc42 in cardiomyocyte proliferation and cell-cell adhesion during heart development. Dev. Biol. *421*, 271–283. https://doi.org/10.1016/j.ydbio.2016.12.012.

210. Bouma, B.J., and Mulder, B.J.M. (2017). Changing landscape of congenital heart disease. Circ. Res. *120*, 908–922. https://doi.org/10.1161/CIRCRESAHA.116.309302.

211. Zhou, W., Cai, H., Li, J., Xu, H., Wang, X., Men, H., Zheng, Y., and Cai, L. (2021). Potential roles of mediator complex subunit 13 in cardiac diseases. Int. J. Biol. Sci. *17*, 328–338. https://doi.org/10.7150/ijbs.52290.

212. Montgomery, R.L., Hullinger, T.G., Semus, H.M., Dickinson, B.A., Seto, A.G., Lynch, J.M., Stack, C., Latimer, P.A., Olson, E.N., and van Rooij, E. (2011). Therapeutic inhibition of miR-208a improves cardiac function and survival during heart failure. Circulation *124*, 1537–1547. https://doi.org/10.1161/CIRCULATIONAHA.111.030932.

213. Callis, T.E., Pandya, K., Seok, H.Y., Tang, R.H., Tatsuguchi, M., Huang, Z.P., Chen, J.F., Deng, Z., Gunn, B., Shumate, J., et al. (2009). MicroRNA-208a is a regulator of cardiac hypertrophy and conduction in mice. J. Clin. Invest. *119*, 2772–2786. https://doi.org/10.1172/JCI36154.

214. Xin, M., Olson, E.N., and Bassel-Duby, R. (2013). Mending broken hearts: cardiac development as a basis for adult heart regeneration and repair. Nat. Rev. Mol. Cell Biol. *14*, 529–541. https://doi.org/10.1038/nrm3619.

215. Ma, E., Gu, X.Q., Wu, X., Xu, T., and Haddad, G.G. (2001). Mutation in pre-mRNA adenosine deaminase markedly attenuates neuronal tolerance to O2 deprivation in Drosophila melanogaster. J. Clin. Invest. *107*, 685–693. https://doi.org/10.1172/JCI11625.

216. Peng, P.L., Zhong, X., Tu, W., Soundarapandian, M.M., Molner, P., Zhu, D., Lau, L., Liu, S., Liu, F., and Lu, Y. (2006). ADAR2-dependent RNA editing of AMPA receptor subunit GluR2 determines vulnerability of neurons in forebrain ischemia. Neuron *49*, 719–733. https://doi.org/10.1016/j.neuron.2006.01.025.

217. Nevo-Caspi, Y., Amariglio, N., Rechavi, G., and Paret, G. (2011). A-to-I RNA editing is induced upon hypoxia. Shock *35*, 585–589. https://doi.org/10.1097/SHK.0b013e31820fe4b7.

218. Zoccal, D.B., Furuya, W.I., Bassi, M., Colombari, D.S.A., and Colombari, E. (2014). The nucleus of the solitary tract and the coordination of respiratory and sympathetic activities. Front. Physiol. *5*, 238. https://doi.org/10.3389/fphys.2014.00238.

219. Heesch, C.M. (1999). Reflexes that control cardiovascular function. Am. J. Physiol. *277*, S234–S243. https://doi.org/10.1152/advances.1999.277.6.S234.

220. Goetze, J.P., Bruneau, B.G., Ramos, H.R., Ogawa, T., de Bold, M.K., and de Bold, A.J. (2020). Cardiac natriuretic peptides. Nat. Rev. Cardiol. *17*, 698–717. https://doi.org/10.1038/s41569-020-0381-0.

221. Laragh, J.H. (1985). Atrial natriuretic hormone, the renin-aldosterone axis, and blood pressure-electrolyte homeostasis. N. Engl. J. Med. *313*, 1330–1340. https://doi.org/10.1056/NEJM198511213132106.

222. Japundžić-Žigon, N., Lozić, M., Šarenac, O., and Murphy, D. (2020). Vasopressin & oxytocin in control of the cardiovascular system: an updated review. Curr. Neuropharmacol. *18*, 14–33. https://doi.org/10.2174/1570159X17666190717150501.

223. Motiejunaite, J., Amar, L., and Vidal-Petiot, E. (2021). Adrenergic receptors and cardiovascular effects of catecholamines. Ann. Endocrinol. *82*, 193–197. https://doi.org/10.1016/j.ando.2020.03.012.

224. Park, D.S., and Fishman, G.I. (2011). The cardiac conduction system. Circulation *123*, 904–915. https://doi.org/10.1161/CIRCULATIONAHA.110.942284.

225. Solaro, R.J. (2007). Mechanisms of the Frank-Starling law of the heart: the beat goes on. Biophys. J. *93*, 4095–4096. https://doi.org/10.1529/biophysj.107.117200.

226. Armstead, W.M. (2016). Cerebral blood flow autoregulation and dysautoregulation. Anesthesiol. Clin. *34*, 465–477. https://doi.org/10.1016/j.anclin.2016.04.002.

227. Carlström, M., Wilcox, C.S., and Arendshorst, W.J. (2015). Renal autoregulation in health and disease. Physiol. Rev. *95*, 405–511. https://doi.org/10.1152/physrev.00042.2012.

228. He, S., Wang, H., Liu, R., He, M., Che, T., Jin, L., Deng, L., Tian, S., Li, Y., Lu, H., et al. (2017). mRNA N6-methyladenosine methylation of postnatal liver development in pig. PLoS One *12*, e0173421. https://doi.org/10.1371/journal.pone.0173421.

229. Chang, M., Lv, H., Zhang, W., Ma, C., He, X., Zhao, S., Zhang, Z.W., Zeng, Y.X., Song, S., Niu, Y., et al. (2017). Region-specific RNA m(6)A methylation represents a new layer of control in the gene regulatory network in the mouse brain. Open Biol. *7*, 170166. https://doi.org/10.1098/rsob.170166.

230. Xi, L., Carroll, T., Matos, I., Luo, J.D., Polak, L., Pasolli, H.A., Jaffrey, S.R., and Fuchs, E. (2020). m6A RNA methylation impacts fate choices during skin morphogenesis. Elife *9*, e56980. https://doi.org/10.7554/eLife.56980.

231. Perry, R.P., Kelley, D.E., Friderici, K., and Rottman, F. (1975). The methylated constituents of L cell messenger RNA: evidence for an unusual cluster at the 5' terminus. Cell *4*, 387–394. https://doi.org/10.1016/0092-8674(75)90159-2.

232. Mayr, C. (2019). What are 3' UTRs doing? Cold Spring Harb. Perspect. Biol. *11*, a034728. https://doi.org/10.1101/cshperspect.a034728.

233. Uhlén, M., Fagerberg, L., Hallström, B.M., Lindskog, C., Oksvold, P., Mardinoglu, A., Sivertsson, Å., Kampf, C., Sjöstedt, E., Asplund, A., et al. (2015). Proteomics. Tissue-based map of the human proteome. Science *347*, 1260419. https://doi.org/10.1126/science.1260419.

234. Hurtubise, J., McLellan, K., Durr, K., Onasanya, O., Nwabuko, D., and Ndisang, J.F. (2016). The different facets of dyslipidemia and hypertension in atherosclerosis. Curr. Atheroscler. Rep. *18*, 82. https://doi.org/10.1007/s11883-016-0632-z.

235. Yildiz, M., Oktay, A.A., Stewart, M.H., Milani, R.V., Ventura, H.O., and Lavie, C.J. (2020). Left ventricular hypertrophy and hypertension. Prog. Cardiovasc. Dis. *63*, 10–21. https://doi.org/10.1016/j.pcad.2019.11.009.

236. Vancheri, F., Longo, G., Vancheri, S., and Henein, M. (2020). Coronary microvascular dysfunction. J. Clin. Med. *9*, E2880. https://doi.org/10.3390/jcm9092880.

237. SPRINT Research Group, Wright, J.T., Jr., Williamson, J.D., Whelton, P.K., Snyder, J.K., Sink, K.M., Rocco, M.V., Reboussin, D.M., Rahman, M., Oparil, S., et al. (2015). A randomized trial of intensive versus standard blood-pressure control. N. Engl. J. Med. *373*, 2103–2116. https://doi.org/10.1056/NEJMoa1511939.

238. Levy, D., Larson, M.G., Vasan, R.S., Kannel, W.B., and Ho, K.K. (1996). The progression from hypertension to congestive heart failure. JAMA *275*, 1557–1562. https://doi.org/10.1001/jama.1996.03530440037034.

239. MacMahon, S., Peto, R., Cutler, J., Collins, R., Sorlie, P., Neaton, J., Abbott, R., Godwin, J., Dyer, A., and Stamler, J. (1990). Blood pressure, stroke, and coronary heart disease. Part 1, Prolonged differences in blood pressure: prospective observational studies corrected for the regression dilution bias. Lancet *335*, 765–774. https://doi.org/10.1016/0140-6736(90)90878-9.

240. Messerli, F.H., Rimoldi, S.F., and Bangalore, S. (2017). The transition from hypertension to heart failure: contemporary update. JACC. Heart Fail. *5*, 543–551. https://doi.org/10.1016/j.jchf.2017.04.012.

241. Hsu, C.Y., McCulloch, C.E., Darbinian, J., Go, A.S., and Iribarren, C. (2005). Elevated blood pressure and risk of end-stage renal disease in subjects without baseline kidney disease. Arch. Intern. Med. *165*, 923–928. https://doi.org/10.1001/archinte.165.8.923.

242. Forouzanfar, M.H., Liu, P., Roth, G.A., Ng, M., Biryukov, S., Marczak, L., Alexander, L., Estep, K., Hassen Abate, K., Akinyemiju, T.F., et al. (2017). Global burden of hypertension and systolic blood pressure of at least 110 to 115 mm Hg, 1990-2015. JAMA *317*, 165–182. https://doi.org/10.1001/jama.2016.19043.

243. Mills, K.T., Stefanescu, A., and He, J. (2020). The global epidemiology of hypertension. Nat. Rev. Nephrol. *16*, 223–237. https://doi.org/10.1038/s41581-019-0244-2.

244. Oleksiewicz, U., Gładych, M., Raman, A.T., Heyn, H., Mereu, E., Chlebanowska, P., Andrzejewska, A., Sozańska, B., Samant, N., Fąk, K., et al. (2017). TRIM28 and interacting KRAB-ZNFs control self-renewal of human pluripotent stem cells through epigenetic repression of pro-differentiation genes. Stem Cell Rep. *9*, 2065–2080. https://doi.org/10.1016/j.stemcr.2017.10.031.

245. Wu, D., Li, G., Deng, M., Song, W., Huang, X., Guo, X., Wu, Z., Wu, S., and Xu, J. (2015). Associations between ADRB1 and CYP2D6 gene polymorphisms and the response to beta-blocker therapy in hypertension. J. Int. Med. Res. *43*, 424–434. https://doi.org/10.1177/0300060514563151.

246. Meyer, T.E., Shiffman, D., Morrison, A.C., Rowland, C.M., Louie, J.Z., Bare, L.A., Ross, D.A., Arellano, A.R., Chasman, D.I., Ridker, P.M., et al. (2009). GOSR2 Lys67Arg is associated with hypertension in whites. Am. J. Hypertens. *22*, 163–168. https://doi.org/10.1038/ajh.2008.336.

247. Guyenet, P.G. (2006). The sympathetic control of blood pressure. Nat. Rev. Neurosci. *7*, 335–346. https://doi.org/10.1038/nrn1902.

248. Gerken, T., Girard, C.A., Tung, Y.C.L., Webby, C.J., Saudek, V., Hewitson, K.S., Yeo, G.S.H., McDonough, M.A., Cunliffe, S., McNeill, L.A., et al. (2007). The obesity-associated FTO gene encodes a 2-oxoglutarate-dependent nucleic acid demethylase. Science *318*, 1469–1472. https://doi.org/10.1126/science.1151710.

249. Kriegel, A.J., Baker, M.A., Liu, Y., Liu, P., Cowley, A.W., Jr., and Liang, M. (2015). Endogenous microRNAs in human microvascular endothelial cells regulate mRNAs encoded by hypertension-related genes. Hypertension *66*, 793–799. https://doi.org/10.1161/HYPERTENSIONAHA.115.05645.

250. Nigita, G., Acunzo, M., Romano, G., Veneziano, D., Laganà, A., Vitiello, M., Wernicke, D., Ferro, A., and Croce, C.M. (2016). microRNA editing in seed region aligns with cellular changes in hypoxic conditions. Nucleic Acids Res. *44*, 6298–6308. https://doi.org/10.1093/nar/gkw532.

251. Nakamura, M., and Sadoshima, J. (2018). Mechanisms of physiological and pathological cardiac hypertrophy. Nat. Rev. Cardiol. *15*, 387–407. https://doi.org/10.1038/s41569-018-0007-y.

252. Retailleau, K., Arhatte, M., Demolombe, S., Peyronnet, R., Baudrie, V., Jodar, M., Bourreau, J., Henrion, D., Offermanns, S., Nakamura, F., et al. (2016). Arterial myogenic activation through smooth muscle filamin A. Cell Rep. *14*, 2050–2058. https://doi.org/10.1016/j.celrep.2016.02.019.

253. Mandras, S.A., Mehta, H.S., and Vaidya, A. (2020). Pulmonary hypertension: a brief guide for clinicians. Mayo Clin. Proc. *95*, 1978–1988. https://doi.org/10.1016/j.mayocp.2020.04.039.

254. Boissier, F., Katsahian, S., Razazi, K., Thille, A.W., Roche-Campo, F., Leon, R., Vivier, E., Brochard, L., Vieillard-Baron, A., Brun-Buisson, C., et al. (2013). Prevalence and prognosis of cor pulmonale during protective ventilation for acute respiratory distress syndrome. Intensive Care Med. *39*, 1725–1733. https://doi.org/10.1007/s00134-013-2941-9.

255. Thenappan, T., Ormiston, M.L., Ryan, J.J., and Archer, S.L. (2018). Pulmonary arterial hypertension: pathogenesis and clinical management. BMJ *360*, j5492. https://doi.org/10.1136/bmj.j5492.

256. Fei, J., Cui, X.B., Wang, J.N., Dong, K., and Chen, S.Y. (2016). ADAR1-Mediated RNA editing, A novel mechanism controlling phenotypic modulation of vascular smooth muscle cells. Circ. Res. *119*, 463–469. https://doi.org/10.1161/CIRCRESAHA.116.309003.

257. Hong, M., Rong, J., Tao, X., and Xu, Y. (2022). The emerging role of ferroptosis in cardiovascular diseases. Front. Pharmacol. *13*, 822083. https://doi.org/10.3389/fphar.2022.822083.

258. Zhu, B., Gong, Y., Shen, L., Li, J., Han, J., Song, B., Hu, L., Wang, Q., and Wang, Z. (2020). Total Panax notoginseng saponin inhibits vascular smooth muscle cell proliferation and migration and intimal hyperplasia by regulating WTAP/p16 signals via m(6)A modulation. Biomed. Pharmacother. *124*, 109935. https://doi.org/10.1016/j.biopha.2020.109935.

259. Kuwahara, K., Saito, Y., Takano, M., Arai, Y., Yasuno, S., Nakagawa, Y., Takahashi, N., Adachi, Y., Takemura, G., Horie, M., et al. (2003). NRSF regulates the fetal cardiac gene program and maintains normal cardiac structure and function. EMBO J. *22*, 6310–6321. https://doi.org/10.1093/emboj/cdg601.

260. Oka, T., Akazawa, H., Naito, A.T., and Komuro, I. (2014). Angiogenesis and cardiac hypertrophy: maintenance of cardiac function and causative roles in heart failure. Circ. Res. *114*, 565–571. https://doi.org/10.1161/CIRCRESAHA.114.300507.

261. Rosca, M.G., Tandler, B., and Hoppel, C.L. (2013). Mitochondria in cardiac hypertrophy and heart failure. J. Mol. Cell. Cardiol. *55*, 31–41. https://doi.org/10.1016/j.yjmcc.2012.09.002.

262. Petrosino, J.M., Hinger, S.A., Golubeva, V.A., Barajas, J.M., Dorn, L.E., Iyer, C.C., Sun, H.L., Arnold, W.D., He, C., and Accornero, F. (2022). The m(6)A methyltransferase METTL3 regulates muscle maintenance and growth in mice. Nat. Commun. *13*, 168. https://doi.org/10.1038/s41467-021-27848-7.

263. Castillero, E., Akashi, H., Najjar, M., Ji, R., Brandstetter, L.M., Wang, C., Liao, X., Zhang, X., Sperry, A., Gailes, M., et al. (2020). Activin type II receptor ligand signaling inhibition after experimental ischemic heart failure attenuates cardiac remodeling and prevents fibrosis. Am. J. Physiol. Heart Circ. Physiol. *318*, H378–H390. https://doi.org/10.1152/ajpheart.00302.

264. Zaccara, S., and Jaffrey, S.R. (2020). A unified model for the function of YTHDF proteins in regulating m(6)a-modified mRNA. Cell *181*, 1582–1595.e18. https://doi.org/10.1016/j.cell.2020.05.012.

265. Hosen, M.R., Militello, G., Weirick, T., Ponomareva, Y., Dassanayaka, S., Moore, J.B., 4th, Döring, C., Wysoczynski, M., Jones, S.P., Dimmeler, S., et al. (2018). Airn regulates Igf2bp2 translation in cardiomyocytes. Circ. Res. *122*, 1347–1353. https://doi.org/10.1161/CIRCRESAHA.117.312215.

266. Cleynen, I., Brants, J.R., Peeters, K., Deckers, R., Debiec-Rychter, M., Sciot, R., Van de Ven, W.J.M., and Petit, M.M.R. (2007). HMGA2 regulates transcription of the Imp2 gene via an intronic regulatory element in cooperation with nuclear factor-kappaB. Mol. Cancer Res. *5*, 363–372. https://doi.org/10.1158/1541-7786.MCR-06-0331.

267. Gallo, S., Vitacolonna, A., Bonzano, A., Comoglio, P., and Crepaldi, T. (2019). ERK: a key player in the pathophysiology of cardiac hypertrophy. Int. J. Mol. Sci. *20*, E2164. https://doi.org/10.3390/ijms20092164.

268. Sun, H.L., Zhu, A.C., Gao, Y., Terajima, H., Fei, Q., Liu, S., Zhang, L., Zhang, Z., Harada, B.T., He, Y.Y., et al. (2020). Stabilization of ERK-phosphorylated METTL3 by USP5 increases m(6)A methylation. Mol. Cell *80*, 633–647.e7. https://doi.org/10.1016/j.molcel.2020.10.026.

269. Karmazyn, M., Purdham, D.M., Rajapurohitam, V., and Zeidan, A. (2007). Leptin as a cardiac hypertrophic factor: a potential target for therapeutics. Trends Cardiovasc. Med. *17*, 206–211. https://doi.org/10.1016/j.tcm.2007.06.001.

270. Nakao, S., Tsukamoto, T., Ueyama, T., and Kawamura, T. (2020). STAT3 for cardiac regenerative medicine: involvement in stem cell biology, pathophysiology, and bioengineering. Int. J. Mol. Sci. *21*, E1937. https://doi.org/10.3390/ijms21061937.

271. Tsukamoto, T., Sogo, T., Ueyama, T., Nakao, S., Harada, Y., Ihara, D., Akagi, Y., Kida, Y.S., Hasegawa, K., Nagamune, T., et al. (2020). Chimeric G-CSF receptor-mediated STAT3 activation contributes to efficient induction of cardiomyocytes from mouse induced pluripotent stem cells. Biotechnol. J. *15*, e1900052. https://doi.org/10.1002/biot.201900052.

272. Wu, R., Liu, Y., Zhao, Y., Bi, Z., Yao, Y., Liu, Q., Wang, F., Wang, Y., and Wang, X. (2019). m(6)A methylation controls pluripotency of porcine induced pluripotent stem cells by targeting SOCS3/JAK2/STAT3 pathway in a YTHDF1/YTHDF2-orchestrated manner. Cell Death Dis. *10*, 171. https://doi.org/10.1038/s41419-019-1417-4.

273. Boissel, S., Reish, O., Proulx, K., Kawagoe-Takaki, H., Sedgwick, B., Yeo, G.S.H., Meyre, D., Golzio, C., Molinari, F., Kadhom, N., et al. (2009). Loss-of-function mutation in the dioxygenase-encoding FTO gene causes severe growth retardation and multiple malformations. Am. J. Hum. Genet. *85*, 106–111. https://doi.org/10.1016/j.ajhg.2009.06.002.

274. Uetrecht, A.C., and Bear, J.E. (2006). Coronins: the return of the crown. Trends Cell Biol. *16*, 421–426. https://doi.org/10.1016/j.tcb.2006.06.002.

275. Lin, L., Hales, C.M., Garber, K., and Jin, P. (2014). Fat mass and obesity-associated (FTO) protein interacts with CaMKII and modulates the activity of CREB signaling pathway. Hum. Mol. Genet. *23*, 3299–3306. https://doi.org/10.1093/hmg/ddu043.

276. Shen, J., Yang, L., and Wei, W. (2021). Role of Fto on CaMKII/CREB signaling pathway of hippocampus in depressive-like behaviors induced by chronic restraint stress mice. Behav. Brain Res. *406*, 113227. https://doi.org/10.1016/j.bbr.2021.113227.

277. Liu, Z., and Zhang, J. (2018). Most m6A RNA modifications in protein-coding regions are evolutionarily unconserved and likely nonfunctional. Mol. Biol. Evol. *35*, 666–675. https://doi.org/10.1093/molbev/msx320.

278. Shi, H., Wang, X., Lu, Z., Zhao, B.S., Ma, H., Hsu, P.J., Liu, C., and He, C. (2017). YTHDF3 facilitates translation and decay of N(6)-methyladenosine-modified RNA. Cell Res. *27*, 315–328. https://doi.org/10.1038/cr.2017.15.

279. Li, A., Chen, Y.S., Ping, X.L., Yang, X., Xiao, W., Yang, Y., Sun, H.Y., Zhu, Q., Baidya, P., Wang, X., et al. (2017). Cytoplasmic m(6)A reader YTHDF3 promotes mRNA translation. Cell Res. *27*, 444–447. https://doi.org/10.1038/cr.2017.10.

280. Worpenberg, L., Paolantoni, C., Longhi, S., Mulorz, M.M., Lence, T., Wessels, H.H., Dassi, E., Aiello, G., Sutandy, F.X.R., Scheibe, M., et al. (2021). Ythdf is a N6-methyladenosine reader that modulates Fmr1 target mRNA selection and restricts axonal growth in Drosophila. EMBO J. *40*, e104975. https://doi.org/10.15252/embj.2020104975.

281. Laggerbauer, B., Ostareck, D., Keidel, E.M., Ostareck-Lederer, A., and Fischer, U. (2001). Evidence that fragile X mental retardation protein is a negative regulator of translation. Hum. Mol. Genet. *10*, 329–338. https://doi.org/10.1093/hmg/10.4.329.

282. Jacquemont, S., Pacini, L., Jønch, A.E., Cencelli, G., Rozenberg, I., He, Y., D'Andrea, L., Pedini, G., Eldeeb, M., Willemsen, R., et al. (2018). Protein synthesis levels are increased in a subset of individuals with fragile X syndrome. Hum. Mol. Genet. *27*, 2039–2051. https://doi.org/10.1093/hmg/ddy099.

283. Bao, J., Ye, C., Zheng, Z., and Zhou, Z. (2018). Fmr1 protects cardiomyocytes against lipopolysaccharide-induced myocardial injury. Exp. Ther. Med. *16*, 1825–1833. https://doi.org/10.3892/etm.2018.6386.

284. Barajas, M., Wang, A., Griffiths, K.K., Matsumoto, K., Liu, R., Homma, S., and Levy, R.J. (2021). The newborn Fmr1 knockout mouse: a novel model of excess ubiquinone and closed mitochondrial permeability transition pore in the developing heart. Pediatr. Res. *89*, 456–463. https://doi.org/10.1038/s41390-020-1064-6.

285. Harrison, B.C., Roberts, C.R., Hood, D.B., Sweeney, M., Gould, J.M., Bush, E.W., and McKinsey, T.A. (2004). The CRM1 nuclear export receptor controls pathological cardiac gene expression. Mol. Cell Biol. *24*, 10636–10649. https://doi.org/10.1128/MCB.24.24.10636-10649.2004.

286. Gao, C., Ren, S., Lee, J.H., Qiu, J., Chapski, D.J., Rau, C.D., Zhou, Y., Abdellatif, M., Nakano, A., Vondriska, T.M., et al. (2016). RBFox1-mediated RNA splicing regulates cardiac hypertrophy and heart failure. J. Clin. Invest. *126*, 195–206. https://doi.org/10.1172/JCI84015.

287. Davis, J.K., and Broadie, K. (2017). Multifarious functions of the fragile X mental retardation protein. Trends Genet. *33*, 703–714. https://doi.org/10.1016/j.tig.2017.07.008.

288. Schultheiss, H.P., Fairweather, D., Caforio, A.L.P., Escher, F., Hershberger, R.E., Lipshultz, S.E., Liu, P.P., Matsumori, A., Mazzanti, A., McMurray, J., et al. (2019). Dilated cardiomyopathy. Nat. Rev. Dis. Primers *5*, 32. https://doi.org/10.1038/s41572-019-0084-1.

289. Herman, D.S., Lam, L., Taylor, M.R.G., Wang, L., Teekakirikul, P., Christodoulou, D., Conner, L., DePalma, S.R., McDonough, B., Sparks, E., et al. (2012). Truncations of titin causing dilated cardiomyopathy. N. Engl. J. Med. *366*, 619–628. https://doi.org/10.1056/NEJMoa1110186.

290. Loescher, C.M., Hobbach, A.J., and Linke, W.A. (2021). Titin (TTN): from molecule to modifications, mechanics and medical significance. Cardiovasc. Res. cvab328. https://doi.org/10.1093/cvr/cvab328.

291. Jia, G., Hill, M.A., and Sowers, J.R. (2018). Diabetic cardiomyopathy: an update of mechanisms contributing to this clinical entity. Circ. Res. *122*, 624–638. https://doi.org/10.1161/CIRCRESAHA.117.311586.

292. Bao, M.H., Feng, X., Zhang, Y.W., Lou, X.Y., Cheng, Y., and Zhou, H.H. (2013). Let-7 in cardiovascular diseases, heart development and cardiovascular differentiation from stem cells. Int. J. Mol. Sci. *14*, 23086–23102. https://doi.org/10.3390/ijms141123086.

293. van Rooij, E., Sutherland, L.B., Thatcher, J.E., DiMaio, J.M., Naseem, R.H., Marshall, W.S., Hill, J.A., and Olson, E.N. (2008). Dysregulation of microRNAs after myocardial infarction reveals a role of miR-29 in cardiac fibrosis. Proc. Natl. Acad. Sci. USA *105*, 13027–13032. https://doi.org/10.1073/pnas.0805038105.

294. Zou, X., Wang, J., Tang, L., and Wen, Q. (2019). LncRNA TUG1 contributes to cardiac hypertrophy via regulating miR-29b-3p. In Vitro Cell. Dev. Biol. Anim. *55*, 482–490. https://doi.org/10.1007/s11626-019-00368-x.

295. Ni, H., Li, W., Zhuge, Y., Xu, S., Wang, Y., Chen, Y., Shen, G., and Wang, F. (2019). Inhibition of circHIPK3 prevents angiotensin II-induced cardiac fibrosis by sponging miR-29b-3p. Int. J. Cardiol. *292*, 188–196. https://doi.org/10.1016/j.ijcard.2019.04.006.

296. Zhao, Y., Samal, E., and Srivastava, D. (2005). Serum response factor regulates a muscle-specific microRNA that targets Hand2 during cardiogenesis. Nature *436*, 214–220. https://doi.org/10.1038/nature03817.

297. Melman, Y.F., Shah, R., and Das, S. (2014). MicroRNAs in heart failure: is the picture becoming less miRky? Circ. Heart Fail. *7*, 203–214. https://doi.org/10.1161/CIRCHEARTFAILURE.113.000266.

298. Connolly, M., Garfield, B.E., Crosby, A., Morrell, N.W., Wort, S.J., and Kemp, P.R. (2020). miR-1-5p targets TGF-betaR1 and is suppressed in the hypertrophying hearts of rats with pulmonary arterial hypertension. PLoS One *15*, e0229409. https://doi.org/10.1371/journal.pone.0229409.

299. Hua, Y., Zhang, Y., and Ren, J. (2012). IGF-1 deficiency resists cardiac hypertrophy and myocardial contractile dysfunction: role of microRNA-1 and microRNA-133a. J. Cell Mol. Med. *16*, 83–95. https://doi.org/10.1111/j.1582-4934.2011.01307.x.

300. Vinther, J., Hedegaard, M.M., Gardner, P.P., Andersen, J.S., and Arctander, P. (2006). Identification of miRNA targets with stable isotope labeling by amino acids in cell culture. Nucleic Acids Res. *34*, e107. https://doi.org/10.1093/nar/gkl590.

301. Lim, L.P., Lau, N.C., Garrett-Engele, P., Grimson, A., Schelter, J.M., Castle, J., Bartel, D.P., Linsley, P.S., and Johnson, J.M. (2005). Microarray analysis shows that some microRNAs downregulate large numbers of target mRNAs. Nature *433*, 769–773. https://doi.org/10.1038/nature03315.

302. Seok, H., Lee, H., Lee, S., Ahn, S.H., Lee, H.S., Kim, G.W.D., Peak, J., Park, J., Cho, Y.K., Jeong, Y., et al. (2020). Position-specific oxidation of miR-1 encodes cardiac hypertrophy. Nature *584*, 279–285. https://doi.org/10.1038/s41586-020-2586-0.

303. Rutsch, F., MacDougall, M., Lu, C., Buers, I., Mamaeva, O., Nitschke, Y., Rice, G.I., Erlandsen, H., Kehl, H.G., Thiele, H., et al. (2015). A specific IFIH1 gain-of-function mutation causes Singleton-Merten syndrome. Am. J. Hum. Genet. *96*, 275–282. https://doi.org/10.1016/j.ajhg.2014.12.014.

304. Isner, J.M., and Losordo, D.W. (1999). Therapeutic angiogenesis for heart failure. Nat. Med. *5*, 491–492. https://doi.org/10.1038/8374.

305. Taimeh, Z., Loughran, J., Birks, E.J., and Bolli, R. (2013). Vascular endothelial growth factor in heart failure. Nat. Rev. Cardiol. *10*, 519–530. https://doi.org/10.1038/nrcardio.2013.94.

306. Chistiakov, D.A., Orekhov, A.N., and Bobryshev, Y.V. (2016). Cardiac-specific miRNA in cardiogenesis, heart function, and cardiac pathology (with focus on myocardial infarction). J. Mol. Cell. Cardiol. *94*, 107–121. https://doi.org/10.1016/j.yjmcc.2016.03.015.

307. Kura, B., Kalocayova, B., Devaux, Y., and Bartekova, M. (2020). Potential clinical implications of miR-1 and miR-21 in heart disease and cardioprotection. Int. J. Mol. Sci. *21*, E700. https://doi.org/10.3390/ijms21030700.

308. Jiang, F., Chen, Q., Wang, W., Ling, Y., Yan, Y., and Xia, P. (2020). Hepatocyte-derived extracellular vesicles promote endothelial inflammation and atherogenesis via microRNA-1. J. Hepatol. *72*, 156–166. https://doi.org/10.1016/j.jhep.2019.09.014.

309. Lacolley, P., Regnault, V., and Laurent, S. (2020). Mechanisms of arterial stiffening: from mechanotransduction to epigenetics. Arterioscler. Thromb. Vasc. Biol. *40*, 1055–1062. https://doi.org/10.1161/ATVBAHA.119.313129.

310. Wanner, C., Amann, K., and Shoji, T. (2016). The heart and vascular system in dialysis. Lancet *388*, 276–284. https://doi.org/10.1016/S0140-6736(16)30508-6.

311. Sluiter, T.J., van Buul, J.D., Huveneers, S., Quax, P.H.A., and de Vries, M.R. (2021). Endothelial barrier function and leukocyte transmigration in atherosclerosis. Biomedicines *9*, 328. https://doi.org/10.3390/biomedicines9040328.

312. He, M., Martin, M., Marin, T., Chen, Z., and Gongol, B. (2020). Endothelial mechanobiology. APL Bioeng. *4*, 010904. https://doi.org/10.1063/1.5129563.

313. Bleda, S., de Haro, J., Varela, C., Esparza, L., Ferruelo, A., and Acin, F. (2014). NLRP1 inflammasome, and not NLRP3, is the key in the shift to proinflammatory state on endothelial cells in peripheral arterial disease. Int. J. Cardiol. *172*, 282–284. https://doi.org/10.1016/j.ijcard.2013.12.201.

314. Bai, L., and Shyy, J.Y.J., PhD (2018). Shear stress regulation of endothelium: a double-edged sword. J. Transl. Int. Med. *6*, 58–61. https://doi.org/10.2478/jtim-2018-0019.

315. Vervloet, M.G., Adema, A.Y., Larsson, T.E., and Massy, Z.A. (2014). The role of klotho on vascular calcification and endothelial function in chronic kidney disease. Semin. Nephrol. *34*, 578–585. https://doi.org/10.1016/j.semnephrol.2014.09.003.

316. Gou, L., Xue, C., Tang, X., and Fang, Z. (2020). Inhibition of Exo-miR-19a-3p derived from cardiomyocytes promotes angiogenesis and improves heart function in mice with myocardial infarction via targeting HIF-1alpha. Aging (Albany NY) *12*, 23609–23618. https://doi.org/10.18632/aging.103563.

317. Jain, T., Nikolopoulou, E.A., Xu, Q., and Qu, A. (2018). Hypoxia inducible factor as a therapeutic target for atherosclerosis. Pharmacol. Ther. *183*, 22–33. https://doi.org/10.1016/j.pharmthera.2017.09.003.

318. Park, M.H., Jeong, E., and Choudhury, M. (2020). Mono-(2-Ethylhexyl)phthalate regulates cholesterol efflux via MicroRNAs regulated m(6)A RNA methylation. Chem. Res. Toxicol. *33*, 461–469. https://doi.org/10.1021/acs.chemrestox.9b00367.

319. Tang, X., Yin, R., Shi, H., Wang, X., Shen, D., Wang, X., and Pan, C. (2020). LncRNA ZFAS1 confers inflammatory responses and reduces cholesterol efflux in atherosclerosis through regulating miR-654-3p-ADAM10/RAB22A axis. Int. J. Cardiol. *315*, 72–80. https://doi.org/10.1016/j.ijcard.2020.03.056.

320. Chen, L., Yao, H., Hui, J.Y., Ding, S.H., Fan, Y.L., Pan, Y.H., Chen, K.H., Wan, J.Q., and Jiang, J.Y. (2016). Global transcriptomic study of atherosclerosis development in rats. Gene *592*, 43–48. https://doi.org/10.1016/j.gene.2016.07.023.

321. Yang, Z., Ma, J., Han, S., Li, X., Guo, H., and Liu, D. (2020). ZFAS1 exerts an oncogenic role via suppressing miR-647 in an m(6)a-dependent manner in cervical cancer. OncoTargets Ther. *13*, 11795–11806. https://doi.org/10.2147/OTT.S274492.

322. Sukhova, G.K., Zhang, Y., Pan, J.H., Wada, Y., Yamamoto, T., Naito, M., Kodama, T., Tsimikas, S., Witztum, J.L., Lu, M.L., et al. (2003). Deficiency of cathepsin S reduces atherosclerosis in LDL receptor-deficient mice. J. Clin. Invest. *111*, 897–906. https://doi.org/10.1172/JCI14915.

323. Reiser, J., Adair, B., and Reinheckel, T. (2010). Specialized roles for cysteine cathepsins in health and disease. J. Clin. Invest. *120*, 3421–3431. https://doi.org/10.1172/JCI42918.

324. Poulsen, C.B., Al-Mashhadi, A.L., von Wachenfeldt, K., Bentzon, J.F., Nielsen, L.B., Al-Mashhadi, R.H., Thygesen, J., Tolbod, L., Larsen, J.R., Frøkiær, J., et al. (2016). Treatment with a human recombinant monoclonal IgG antibody against oxidized LDL in atherosclerosis-prone pigs reduces cathepsin S in coronary lesions. Int. J. Cardiol. *215*, 506–515. https://doi.org/10.1016/j.ijcard.2016.03.222.

325. Kojima, Y., Volkmer, J.P., McKenna, K., Civelek, M., Lusis, A.J., Miller, C.L., Direnzo, D., Nanda, V., Ye, J., Connolly, A.J., et al. (2016). CD47-blocking antibodies restore phagocytosis and prevent atherosclerosis. Nature *536*, 86–90. https://doi.org/10.1038/nature18935.

326. Kong, X.Y., Vik, E.S., Nawaz, M.S., Berges, N., Dahl, T.B., Vågbø, C., Suganthan, R., Segers, F., Holm, S., Quiles-Jiménez, A., et al. (2020). Deletion of Endonuclease V suppresses chemically induced hepatocellular carcinoma. Nucleic Acids Res. *48*, 4463–4479. https://doi.org/10.1093/nar/gkaa115.

327. Libby, P., Nahrendorf, M., and Swirski, F.K. (2016). Leukocytes link local and systemic inflammation in ischemic cardiovascular disease: an expanded "cardiovascular continuum". J. Am. Coll. Cardiol. *67*, 1091–1103. https://doi.org/10.1016/j.jacc.2015.12.048.

328. Murphy, A.J., Akhtari, M., Tolani, S., Pagler, T., Bijl, N., Kuo, C.L., Wang, M., Sanson, M., Abramowicz, S., Welch, C., et al. (2011). ApoE regulates hematopoietic stem cell proliferation, monocytosis, and monocyte accumulation in atherosclerotic lesions in mice. J. Clin. Invest. *121*, 4138–4149. https://doi.org/10.1172/JCI57559.

329. Robbins, C.S., Chudnovskiy, A., Rauch, P.J., Figueiredo, J.L., Iwamoto, Y., Gorbatov, R., Etzrodt, M., Weber, G.F., Ueno, T., van Rooijen, N., et al. (2012). Extramedullary hematopoiesis generates Ly-6C(high) monocytes that infiltrate atherosclerotic lesions. Circulation *125*, 364–374. https://doi.org/10.1161/CIRCULATIONAHA.111.061986.

330. Heyde, A., Rohde, D., McAlpine, C.S., Zhang, S., Hoyer, F.F., Gerold, J.M., Cheek, D., Iwamoto, Y., Schloss, M.J., Vandoorne, K., et al. (2021). Increased stem cell proliferation in atherosclerosis accelerates clonal hematopoiesis. Cell *184*, 1348–1361.e22. https://doi.org/10.1016/j.cell.2021.01.049.

331. Vu, L.P., Pickering, B.F., Cheng, Y., Zaccara, S., Nguyen, D., Minuesa, G., Chou, T., Chow, A., Saletore, Y., MacKay, M., et al. (2017). The N(6)-methyladenosine (m(6)A)-forming enzyme METTL3 controls myeloid differentiation of normal hematopoietic and leukemia cells. Nat. Med. *23*, 1369–1376. https://doi.org/10.1038/nm.4416.

332. Weng, H., Huang, H., Wu, H., Qin, X., Zhao, B.S., Dong, L., Shi, H., Skibbe, J., Shen, C., Hu, C., et al. (2018). METTL14 inhibits hematopoietic stem/progenitor differentiation and promotes leukemogenesis via mRNA m(6)A modification. Cell Stem Cell *22*, 191–205.e9. https://doi.org/10.1016/j.stem.2017.11.016.

333. Li, Z., Qian, P., Shao, W., Shi, H., He, X.C., Gogol, M., Yu, Z., Wang, Y., Qi, M., Zhu, Y., et al. (2018). Suppression of m(6)A reader Ythdf2 promotes hematopoietic stem cell expansion. Cell Res. *28*, 904–917. https://doi.org/10.1038/s41422-018-0072-0.

334. Mapperley, C., van de Lagemaat, L.N., Lawson, H., Tavosanis, A., Paris, J., Campos, J., Wotherspoon, D., Durko, J., Sarapuu, A., Choe, J., et al. (2021). The mRNA m6A reader YTHDF2 suppresses proinflammatory pathways and sustains hematopoietic stem cell function. J. Exp. Med. *218*, e20200829. https://doi.org/10.1084/jem.20200829.

335. Yin, L., Tang, Y., and Jiang, M. (2021). Research on the circular RNA bioinformatics in patients with acute myocardial infarction. J. Clin. Lab. Anal. *35*, e23621. https://doi.org/10.1002/jcla.23621.

336. Min, J.K., Park, H., Choi, H.J., Kim, Y., Pyun, B.J., Agrawal, V., Song, B.W., Jeon, J., Maeng, Y.S., Rho, S.S., et al. (2011). The WNT antagonist Dickkopf2 promotes angiogenesis in rodent and human endothelial cells. J. Clin. Invest. *121*, 1882–1893. https://doi.org/10.1172/JCI42556.

337. Han, P., Li, W., Lin, C.H., Yang, J., Shang, C., Nuernberg, S.T., Jin, K.K., Xu, W., Lin, C.Y., Lin, C.J., et al. (2014). A long noncoding RNA protects the heart from pathological hypertrophy. Nature *514*, 102–106. https://doi.org/10.1038/nature13596.

338. Xu, Y., Luo, Y., Liang, C., and Zhang, T. (2020). LncRNA-Mhrt regulates cardiac hypertrophy by modulating the miR-145a-5p/KLF4/myocardin axis. J. Mol. Cell. Cardiol. *139*, 47–61. https://doi.org/10.1016/j.yjmcc.2019.12.013.

339. Forini, F., Nicolini, G., Kusmic, C., D'Aurizio, R., Mercatanti, A., Iervasi, G., and Pitto, L. (2020). T3 critically affects the Mhrt/brg1 Axis to regulate the cardiac MHC switch: role of an epigenetic cross-talk. Cells *9*, 155. https://doi.org/10.3390/cells9102155.

340. Zhang, J., Gao, C., Meng, M., and Tang, H. (2016). Long noncoding RNA MHRT protects cardiomyocytes against H2O2-induced apoptosis. Biomol. Ther. *24*, 19–24. https://doi.org/10.4062/biomolther.2015.066.

341. Zhang, L., Wu, Y.J., and Zhang, S.L. (2019). Circulating lncRNA MHRT predicts survival of patients with chronic heart failure. J. Geriatr. Cardiol. *16*, 818–821. https://doi.org/10.11909/j.issn.1671-5411.2019.11.006.

342. Yang, C., Fan, Z., and Yang, J. (2020). m(6)A modification of LncRNA MALAT1: a novel therapeutic target for myocardial ischemia-reperfusion injury. Int. J. Cardiol. *306*, 162. https://doi.org/10.1016/j.ijcard.2019.11.140.

343. Misquitta, C.M., Iyer, V.R., Werstiuk, E.S., and Grover, A.K. (2001). The role of 3'-untranslated region (3'-UTR) mediated mRNA stability in cardiovascular pathophysiology. Mol. Cell. Biochem. *224*, 53–67. https://doi.org/10.1023/a:1011982932645.

344. Bedi, R.K., Huang, D., Eberle, S.A., Wiedmer, L., Śledź, P., and Caflisch, A. (2020). Small-molecule inhibitors of METTL3, the major human epitranscriptomic writer. ChemMedChem *15*, 744–748. https://doi.org/10.1002/cmdc.202000011.

345. Yankova, E., Blackaby, W., Albertella, M., Rak, J., De Braekeleer, E., Tsagkogeorga, G., Pilka, E.S., Aspris, D., Leggate, D., Hendrick, A.G., et al. (2021). Small-molecule inhibition of METTL3 as a strategy against myeloid leukaemia. Nature *593*, 597–601. https://doi.org/10.1038/s41586-021-03536-w.

346. Shen, Z., Zeng, L., and Zhang, Z. (2020). Translatome and transcriptome profiling of hypoxic-induced rat cardiomyocytes. Mol. Ther. Nucleic Acids *22*, 1016–1024. https://doi.org/10.1016/j.omtn.2020.10.019.

347. Osnabrugge, R.L.J., Mylotte, D., Head, S.J., Van Mieghem, N.M., Nkomo, V.T., LeReun, C.M., Bogers, A.J.J.C., Piazza, N., and Kappetein, A.P. (2013). Aortic stenosis in the elderly: disease prevalence and number of candidates for transcatheter aortic valve replacement: a meta-analysis and modeling study. J. Am. Coll. Cardiol. *62*, 1002–1012. https://doi.org/10.1016/j.jacc.2013.05.015.

348. Lindman, B.R., Clavel, M.A., Mathieu, P., Iung, B., Lancellotti, P., Otto, C.M., and Pibarot, P. (2016). Calcific aortic stenosis. Nat. Rev. Dis. Primers *2*, 16006. https://doi.org/10.1038/nrdp.2016.6.

349. Towler, D.A. (2013). Molecular and cellular aspects of calcific aortic valve disease. Circ. Res. *113*, 198–208. https://doi.org/10.1161/CIRCRESAHA.113.300155.

350. Li, H., Wu, H., Wang, Q., Ning, S., Xu, S., and Pang, D. (2021). Dual effects of N(6)-methyladenosine on cancer progression and immunotherapy. Mol. Ther. Nucleic Acids *24*, 25–39. https://doi.org/10.1016/j.omtn.2021.02.001.

351. Shi, Y.N., Zhu, N., Liu, C., Wu, H.T., Gui, Y., Liao, D.F., and Qin, L. (2017). Wnt5a and its signaling pathway in angiogenesis. Clin. Chim. Acta *471*, 263–269. https://doi.org/10.1016/j.cca.2017.06.017.

352. Menden, H., Welak, S., Cossette, S., Ramchandran, R., and Sampath, V. (2015). Lipopolysaccharide (LPS)-mediated angiopoietin-2-dependent autocrine angiogenesis is regulated by NADPH oxidase 2 (Nox2) in human pulmonary microvascular endothelial cells. J. Biol. Chem. *290*, 5449–5461. https://doi.org/10.1074/jbc.M114.600692.

353. Namiki, A., Brogi, E., Kearney, M., Kim, E.A., Wu, T., Couffinhal, T., Varticovski, L., and Isner, J.M. (1995). Hypoxia induces vascular endothelial growth factor in cultured human endothelial cells. J. Biol. Chem. *270*, 31189–31195. https://doi.org/10.1074/jbc.270.52.31189.

354. Iruela-Arispe, M.L., Bornstein, P., and Sage, H. (1991). Thrombospondin exerts an antiangiogenic effect on cord formation by endothelial cells in vitro. Proc. Natl. Acad. Sci. USA *88*, 5026–5030. https://doi.org/10.1073/pnas.88.11.5026.

355. DiPietro, L.A., and Polverini, P.J. (1993). Angiogenic macrophages produce the angiogenic inhibitor thrombospondin 1. Am. J. Pathol. *143*, 678–684.

356. Franco, C.A., Liebner, S., and Gerhardt, H. (2009). Vascular morphogenesis: a Wnt for every vessel? Curr. Opin. Genet. Dev. *19*, 476–483. https://doi.org/10.1016/j.gde.2009.09.004.

357. van de Schans, V.A.M., Smits, J.F.M., and Blankesteijn, W.M. (2008). The Wnt/frizzled pathway in cardiovascular development and disease: friend or foe? Eur. J. Pharmacol. *585*, 338–345. https://doi.org/10.1016/j.ejphar.2008.02.093.

358. Gallicano, G.I., Bauer, C., and Fuchs, E. (2001). Rescuing desmoplakin function in extra-embryonic ectoderm reveals the importance of this protein in embryonic heart, neuroepithelium, skin and vasculature. Development *128*, 929–941. https://doi.org/10.1242/dev.128.6.929.

359. Zhou, X., Stuart, A., Dettin, L.E., Rodriguez, G., Hoel, B., and Gallicano, G.I. (2004). Desmoplakin is required for microvascular tube formation in culture. J. Cell Sci. *117*, 3129–3140. https://doi.org/10.1242/jcs.01132.

360. Du, H., Zhao, Y., He, J., Zhang, Y., Xi, H., Liu, M., Ma, J., and Wu, L. (2016). YTHDF2 destabilizes m(6)A-containing RNA through direct recruitment of the CCR4-NOT deadenylase complex. Nat. Commun. *7*, 12626. https://doi.org/10.1038/ncomms12626.

361. Welten, S.M.J., Bastiaansen, A.J.N.M., de Jong, R.C.M., de Vries, M.R., Peters, E.A.B., Boonstra, M.C., Sheikh, S.P., La Monica, N., Kandimalla, E.R., Quax, P.H.A., et al. (2014). Inhibition of 14q32 MicroRNAs miR-329, miR-487b,

miR-494, and miR-495 increases neovascularization and blood flow recovery after ischemia. Circ. Res. *115*, 696–708. https://doi.org/10.1161/CIRCRESAHA.114.304747.

362. Isselbacher, E.M., Lino Cardenas, C.L., and Lindsay, M.E. (2016). Hereditary influence in thoracic aortic aneurysm and dissection. Circulation *133*, 2516–2528. https://doi.org/10.1161/CIRCULATIONAHA.116.009762.

363. Kim, H.W., and Stansfield, B.K. (2017). Genetic and epigenetic regulation of aortic aneurysms. BioMed Res. Int. *2017*, 7268521. https://doi.org/10.1155/2017/7268521.

364. Chalouhi, N., Hoh, B.L., and Hasan, D. (2013). Review of cerebral aneurysm formation, growth, and rupture. Stroke *44*, 3613–3622. https://doi.org/10.1161/STROKEAHA.113.002390.

365. Schievink, W.I. (1997). Intracranial aneurysms. N. Engl. J. Med. *336*, 28–40. https://doi.org/10.1056/NEJM199701023360106.

366. Quintana, R.A., and Taylor, W.R. (2019). Cellular mechanisms of aortic aneurysm formation. Circ. Res. *124*, 607–618. https://doi.org/10.1161/CIRCRESAHA.118.313187.

367. Sakalihasan, N., Limet, R., and Defawe, O.D. (2005). Abdominal aortic aneurysm. Lancet *365*, 1577–1589. https://doi.org/10.1016/S0140-6736(05)66459-8.

368. Nienaber, C.A., and Clough, R.E. (2015). Management of acute aortic dissection. Lancet *385*, 800–811. https://doi.org/10.1016/S0140-6736(14)61005-9.

369. Erbel, R., Aboyans, V., Boileau, C., Bossone, E., Bartolomeo, R.D., Eggebrecht, H., Evangelista, A., Falk, V., Frank, H., Gaemperli, O., et al. (2014). 2014 ESC Guidelines on the diagnosis and treatment of aortic diseases: document covering acute and chronic aortic diseases of the thoracic and abdominal aorta of the adult. The Task Force for the Diagnosis and Treatment of Aortic Diseases of the European Society of Cardiology (ESC). Eur. Heart J. *35*, 2873–2926. https://doi.org/10.1093/eurheartj/ehu281.

370. Wanhainen, A., Verzini, F., Van Herzeele, I., Allaire, E., Bown, M., Cohnert, T., Dick, F., van Herwaarden, J., Karkos, C., Koelemay, M., et al. (2019). 's choice - European society for vascular surgery (ESVS) 2019 clinical practice guidelines on the management of abdominal aorto-iliac artery aneurysms. Eur. J. Vasc. Endovasc. Surg. *57*, 8–93. https://doi.org/10.1016/j.ejvs.2018.09.020.

371. Wang, B., Sun, J., Kitamoto, S., Yang, M., Grubb, A., Chapman, H.A., Kalluri, R., and Shi, G.P. (2006). Cathepsin S controls angiogenesis and tumor growth via matrix-derived angiogenic factors. J. Biol. Chem. *281*, 6020–6029. https://doi.org/10.1074/jbc.M509134200.

372. Shi, G.P., Sukhova, G.K., Kuzuya, M., Ye, Q., Du, J., Zhang, Y., Pan, J.H., Lu, M.L., Cheng, X.W., Iguchi, A., et al. (2003). Deficiency of the cysteine protease cathepsin S impairs microvessel growth. Circ. Res. *92*, 493–500. https://doi.org/10.1161/01.RES.0000060485.20318.96.

373. Riese, R.J., Mitchell, R.N., Villadangos, J.A., Shi, G.P., Palmer, J.T., Karp, E.R., De Sanctis, G.T., Ploegh, H.L., and Chapman, H.A. (1998). Cathepsin S activity regulates antigen presentation and immunity. J. Clin. Invest. *101*, 2351–2363. https://doi.org/10.1172/JCI1158.

374. Xia, L., Sun, C., Zhu, H., Zhai, M., Zhang, L., Jiang, L., Hou, P., Li, J., Li, K., Liu, Z., et al. (2020). Melatonin protects against thoracic aortic aneurysm and dissection through SIRT1-dependent regulation of oxidative stress and vascular smooth muscle cell loss. J. Pineal Res. *69*, e12661. https://doi.org/10.1111/jpi.12661.

375. Yang, L., Liu, X., Song, L., Su, G., Di, A., Bai, C., Wei, Z., and Li, G. (2020). Melatonin restores the pluripotency of long-term-cultured embryonic stem cells through melatonin receptor-dependent m6A RNA regulation. J. Pineal Res. *69*, e12669. https://doi.org/10.1111/jpi.12669.

376. Song, T., Yang, Y., Wei, H., Xie, X., Lu, J., Zeng, Q., Peng, J., Zhou, Y., Jiang, S., and Peng, J. (2019). Zfp217 mediates m6A mRNA methylation to orchestrate transcriptional and post-transcriptional regulation to promote adipogenic differentiation. Nucleic Acids Res. *47*, 6130–6144. https://doi.org/10.1093/nar/gkz312.

377. Ma, Q., Reiter, R.J., and Chen, Y. (2020). Role of melatonin in controlling angiogenesis under physiological and pathological conditions. Angiogenesis *23*, 91–104. https://doi.org/10.1007/s10456-019-09689-7.

378. Kaur, C., Sivakumar, V., Yong, Z., Lu, J., Foulds, W.S., and Ling, E.A. (2007). Blood-retinal barrier disruption and ultrastructural changes in the hypoxic retina in adult rats: the beneficial effect of melatonin administration. J. Pathol. *212*, 429–439. https://doi.org/10.1002/path.2195.

379. Kaur, C., Sivakumar, V., Foulds, W.S., Luu, C.D., and Ling, E.A. (2009). Cellular and vascular changes in the retina of neonatal rats after an acute exposure to hypoxia. Invest. Ophthalmol. Vis. Sci. *50*, 5364–5374. https://doi.org/10.1167/iovs.09-3552.

380. Cheng, J., Yang, H.L., Gu, C.J., Liu, Y.K., Shao, J., Zhu, R., He, Y.Y., Zhu, X.Y., and Li, M.Q. (2019). Melatonin restricts the viability and angiogenesis of vascular endothelial cells by suppressing HIF-1alpha/ROS/VEGF. Int. J. Mol. Med. *43*, 945–955. https://doi.org/10.3892/ijmm.2018.4021.

381. Sohn, E.J., Won, G., Lee, J., Lee, S., and Kim, S.H. (2015). Upregulation of miRNA3195 and miRNA374b mediates the anti-angiogenic properties of melatonin in hypoxic PC-3 prostate cancer cells. J. Cancer *6*, 19–28. https://doi.org/10.7150/jca.9591.

382. Wang, G., Dai, Y., Li, K., Cheng, M., Xiong, G., Wang, X., Chen, S., Chen, Z., Chen, J., Xu, X., et al. (2021). Deficiency of Mettl3 in bladder cancer stem cells inhibits bladder cancer progression and angiogenesis. Front. Cell Dev. Biol. *9*, 627706. https://doi.org/10.3389/fcell.2021.627706.

383. Yang, Z., Wang, T., Wu, D., Min, Z., Tan, J., and Yu, B. (2020). RNA N6-methyladenosine reader IGF2BP3 regulates cell cycle and angiogenesis in colon cancer. J. Exp. Clin. Cancer Res. *39*, 203. https://doi.org/10.1186/s13046-020-01714-8.

384. Xu, B., Iida, Y., Glover, K.J., Ge, Y., Wang, Y., Xuan, H., Hu, X., Tanaka, H., Wang, W., Fujimura, N., et al. (2019). Inhibition of VEGF (vascular endothelial growth factor)-A or its receptor activity suppresses experimental aneurysm progression in the aortic elastase infusion model. Arterioscler. Thromb. Vasc. Biol. *39*, 1652–1666. https://doi.org/10.1161/ATVBAHA.119.312497.

385. Zheng, B., Zheng, C.Y., Zhang, Y., Yin, W.N., Li, Y.H., Liu, C., Zhang, X.H., Nie, C.J., Zhang, H., Jiang, W., et al. (2018). Regulatory crosstalk between KLF5, miR-29a and Fbw7/CDC4 cooperatively promotes atherosclerotic development. Biochim. Biophys. Acta, Mol. Basis Dis. *1864*, 374–386. https://doi.org/10.1016/j.bbadis.2017.10.021.

386. Klinge, C.M., Piell, K.M., Tooley, C.S., and Rouchka, E.C. (2019). HNRNPA2/B1 is upregulated in endocrine-resistant LCC9 breast cancer cells and alters the miRNA transcriptome when overexpressed in MCF-7 cells. Sci. Rep. *9*, 9430. https://doi.org/10.1038/s41598-019-45636-8.

387. Nagai, R., Suzuki, T., Aizawa, K., Shindo, T., and Manabe, I. (2005). Significance of the transcription factor KLF5 in cardiovascular remodeling. J. Thromb. Haemost. *3*, 1569–1576. https://doi.org/10.1111/j.1538-7836.2005.01366.x.

388. Takyar, S., Vasavada, H., Zhang, J.G., Ahangari, F., Niu, N., Liu, Q., Lee, C.G., Cohn, L., and Elias, J.A. (2013). VEGF controls lung Th2 inflammation via the miR-1-Mpl (myeloproliferative leukemia virus oncogene)-P-selectin axis. J. Exp. Med. *210*, 1993–2010. https://doi.org/10.1084/jem.20121200.

389. Arguello, A.E., DeLiberto, A.N., and Kleiner, R.E. (2017). RNA chemical proteomics reveals the N(6)-methyladenosine (m(6)A)-Regulated protein-RNA interactome. J. Am. Chem. Soc. *139*, 17249–17252. https://doi.org/10.1021/jacs.7b09213.

390. Edupuganti, R.R., Geiger, S., Lindeboom, R.G.H., Shi, H., Hsu, P.J., Lu, Z., Wang, S.Y., Baltissen, M.P.A., Jansen, P.W.T.C., Rossa, M., et al. (2017). N(6)-methyladenosine (m(6)A) recruits and repels proteins to regulate mRNA homeostasis. Nat. Struct. Mol. Biol. *24*, 870–878. https://doi.org/10.1038/nsmb.3462.

391. Patil, D.P., Pickering, B.F., and Jaffrey, S.R. (2018). Reading m(6)A in the transcriptome: m(6)a-binding proteins. Trends Cell Biol. *28*, 113–127. https://doi.org/10.1016/j.tcb.2017.10.001.

392. Xiong, X., Hou, L., Park, Y.P., Molinie, B., GTEx Consortium, Gregory, R.I., and Kellis, M. (2021). Genetic drivers of m(6)A methylation in human brain, lung, heart and muscle. Nat. Genet. *53*, 1156–1165. https://doi.org/10.1038/s41588-021-00890-3.

393. Selberg, S., Blokhina, D., Aatonen, M., Koivisto, P., Siltanen, A., Mervaala, E., Kankuri, E., and Karelson, M. (2019). Discovery of small molecules that activate RNA methylation through cooperative binding to the METTL3-14-WTAP complex active site. Cell Rep. *26*, 3762–3771.e5. https://doi.org/10.1016/j.celrep.2019.02.100.

394. Selberg, S., Yu, L.Y., Bondarenko, O., Kankuri, E., Seli, N., Kovaleva, V., Herodes, K., Saarma, M., and Karelson, M. (2021). Small-molecule inhibitors of the RNA M6A demethylases FTO potently support the survival of dopamine neurons. Int. J. Mol. Sci. *22*, 4537. https://doi.org/10.3390/ijms22094537.

395. Selberg, S., Seli, N., Kankuri, E., and Karelson, M. (2021). Rational design of novel anticancer small-molecule RNA m6A demethylase ALKBH5 inhibitors. ACS Omega *6*, 13310–13320. https://doi.org/10.1021/acsomega.1c01289.

396. You, Y., Fu, Y., Huang, M., Shen, D., Zhao, B., Liu, H., Zheng, Y., and Huang, L. (2022). Recent advances of m6A demethylases inhibitors and their biological functions in human diseases. Int. J. Mol. Sci. *23*, 5815. https://doi.org/10.3390/ijms23105815.

397. Paris, J., Morgan, M., Campos, J., Spencer, G.J., Shmakova, A., Ivanova, I., Mapperley, C., Lawson, H., Wotherspoon, D.A., Sepulveda, C., et al. (2019). Targeting the RNA m(6)A reader YTHDF2 selectively compromises cancer stem cells in acute myeloid leukemia. Cell Stem Cell *25*, 137–148.e6. https://doi.org/10.1016/j.stem.2019.03.021.

398. Lasman, L., Krupalnik, V., Viukov, S., Mor, N., Aguilera-Castrejon, A., Schneir, D., Bayerl, J., Mizrahi, O., Peles, S., Tawil, S., et al. (2020). Context-dependent functional compensation between Ythdf m(6)A reader proteins. Genes Dev. *34*, 1373–1391. https://doi.org/10.1101/gad.340695.120.

399. Hou, G., Zhao, X., Li, L., Yang, Q., Liu, X., Huang, C., Lu, R., Chen, R., Wang, Y., Jiang, B., et al. (2021). SUMOylation of YTHDF2 promotes mRNA degradation and cancer progression by increasing its binding affinity with m6A-modified mRNAs. Nucleic Acids Res. *49*, 2859–2877. https://doi.org/10.1093/nar/gkab065.

400. Zou, Z., Sepich-Poore, C., Zhou, X., Wei, J., and He, C. (2022). The mechanism underlying redundant functions of the YTHDF proteins. Preprint at bioRxiv. https://doi.org/10.1101/2022.05.05.490669.

401. Schöller, E., Weichmann, F., Treiber, T., Ringle, S., Treiber, N., Flatley, A., Feederle, R., Bruckmann, A., and Meister, G. (2018). Interactions, localization, and phosphorylation of the m(6)A generating METTL3-METTL14-WTAP complex. RNA *24*, 499–512. https://doi.org/10.1261/rna.064063.117.

402. Birkaya, B., Ortt, K., and Sinha, S. (2007). Novel in vivo targets of DeltaNp63 in keratinocytes identified by a modified chromatin immunoprecipitation approach. BMC Mol. Biol. *8*, 43. https://doi.org/10.1186/1471-2199-8-43.

403. Taegtmeyer, H. (1978). Metabolic responses to cardiac hypoxia. Increased production of succinate by rabbit papillary muscles. Circ. Res. *43*, 808–815. https://doi.org/10.1161/01.res.43.5.808.

404. Chinopoulos, C. (2013). Which way does the citric acid cycle turn during hypoxia? The critical role of alpha-ketoglutarate dehydrogenase complex. J. Neurosci. Res. *91*, 1030–1043. https://doi.org/10.1002/jnr.23196.

405. Wise, D.R., Ward, P.S., Shay, J.E.S., Cross, J.R., Gruber, J.J., Sachdeva, U.M., Platt, J.M., DeMatteo, R.G., Simon, M.C., and Thompson, C.B. (2011). Hypoxia promotes isocitrate dehydrogenase-dependent carboxylation of alpha-ketoglutarate to citrate to support cell growth and viability. Proc. Natl. Acad. Sci. USA *108*, 19611–19616. https://doi.org/10.1073/pnas.1117773108.

406. Karlstaedt, A., Faubert, B., Vitrac, H.M., Salazar, R.L., Gould, B.D., DeBerardinis, R., and Taegtmeyer, H. (2020). Abstract 308: reductive carboxylation contributes to cardiac adaptation in response to the oncometabolite D2-hydroxyglutarate. Circ. Res. *127*, A308. https://doi.org/10.1161/res.127.suppl.

407. Pisarenko, O.I., Solomatina, E.S., Studneva, I.M., Ivanov, V.E., Kapelko, V.I., and Smirnov, V.N. (1983). Effect of exogenous amino acids on the contractility and nitrogenous metabolism of anoxic heart. Adv. Myocardiol. *4*, 309–318. https://doi.org/10.1007/978-1-4757-4441-5_27.

408. Bittl, J.A., and Shine, K.I. (1983). Protection of ischemic rabbit myocardium by glutamic acid. Am. J. Physiol. *245*, H406–H412. https://doi.org/10.1152/ajpheart.1983.245.3.H406.

409. Matsuoka, S., Jarmakani, J.M., Young, H.H., Uemura, S., and Nakanishi, T. (1986). The effect of glutamate on hypoxic newborn rabbit heart. J. Mol. Cell. Cardiol. *18*, 897–906. https://doi.org/10.1016/s0022-2828(86)80004-9.

410. Gatsiou, A., and Stellos, K. (2018). Dawn of epitranscriptomic medicine. Circ. Genom. Precis. Med. *11*, e001927. https://doi.org/10.1161/CIRCGEN.118.001927.

411. Zhao, B.S., Nachtergaele, S., Roundtree, I.A., and He, C. (2018). Our views of dynamic N(6)-methyladenosine RNA methylation. RNA *24*, 268–272. https://doi.org/10.1261/rna.064295.117.

412. Sikorski, V., Karjalainen, P., Blokhina, D., Oksaharju, K., Khan, J., Katayama, S., Rajala, H., Suihko, S., Tuohinen, S., Teittinen, K., et al. (2021). Epitranscriptomics of ischemic heart disease—the IHD-EPITRAN study design and objectives. Int. J. Mol. Sci. *22*, 6630. https://doi.org/10.3390/ijms22126630.

OMTN, Volume *29*

Supplemental information

Emerging roles of the RNA modifications N6-methyladenosine and adenosine-to-inosine in cardiovascular diseases

Vilbert Sikorski, Antti Vento, Esko Kankuri, and on behalf of the IHD-EPITRAN Consortium

TABLE S1. Summary of observational study results assessing m^{6}A and its core regulators in cardiovascular physiology and diseases

Rough methodological classification: ***, interventional study with experimental evidence either *in vivo,* with multiple lines *in vitro*, or both; *, mainly observational study with direct samples from the target tissue or organ, possibly few interventional methods *in vitro*; *o*, associative/phenotypic study with indirect samples (such as blood), no interventional methods. The degree of certainty for summarized potential therapeutic effect is divided here to be either high (experimental evidence *in vivo*), intermediate† (experimental evidence *in vitro*), or light†† (associative/phenotype evidence). To review the Table S1, please see the Supplementary excel file. Abbreviations are listed within the Supplementary excel as well.

TABLE S2. Summary of observational and interventional discoveries regarding A-to-I editing in cardiovascular physiology and diseases

Methodological classification: ***, interventional study with experimental evidence either *in vivo,* with multiple lines *in vitro* or both; *, mainly observational study with direct samples from the target tissue or organ, possibly few interventional methods *in vitro*; *o*, associative/phenotypic study with indirect samples (such as blood), no interventional methods. The degree of certainty for summarized potential therapeutic effect is divided here to be either high (experimental evidence *in vivo*), intermediate† (experimental evidence *in vitro*), or light†† (associative/phenotype evidence). Blue arrows indicate either overexpression or knockdown. To review the Table S2, please see the Supplementary excel file. Abbreviations are listed within the Supplementary excel as well.

TABLE S3. Summary of studies assessing the effects of m^{6}A manipulation in cardiovascular pathologies or related bioprocesses. While upwards arrow indicates upregulation or increase in the given process in general, downwards arrow indicates downregulation or decrease. To review the Table S3, please see the Supplementary excel file. Abbreviations are listed within the Supplementary excel as well.

TABLE S4. Summary of molecular pathways involving m^{6}A and A-to-I modifications in CVDs. Upwards and downwards arrows indicate upregulation (activation) or downregulation (inhibition), respectively. Blunt arrows represent inhibition.

Abbreviations: ***ADAR1***, adenosine deaminase RNA specific; ***ADAR2***, adenosine deaminase RNA specific B1; ***AGO2***, argonaute RISC catalytic component 2; ***AKT***, AKT serine/threonine kinase; ***ALKBH5***, alkB homolog 5, RNA demethylase; ***AngII***, angiotensin II; ***ANK2***, ankyrin 2; ***ATF4***, activating transcription factor 4; ***AUF1***, ARE/poly(U)-binding/degradation factor 1, alias ***HNRNPD***, heterogeneous nuclear ribonucleoprotein D; ***AVC***, aortic valve calcification; ***BCL2***, BCL2 apoptosis regulator; ***CCL2***, C-C motif chemokine ligand 2; ***CDC42***, cell division cycle 42; ***CDR1as***, cdr1 antisense (a long non-coding RNA); ***CHAPIR***, Cardiac-hypertrophy-associated piRNA (piwi-interacting RNA); ***Chast***, cardiac hypertrophy–associated transcript (a long non-coding RNA); ***CHOP***, C/EBP homologous protein; ***circCELF1***, circular RNA CUGBP Elav-like family member 1; ***CM***, cardiomyocyte; ***CMYA5***, cardiomyopathy associated 5; ***CPT-1A***, carnitine palmitoyltransferase 1A; ***CTNND1***, catenin delta 1; ***CTSL***, cathepsin L; ***CTSS***, cathepsin S; ***CUX1p110***, cut like homeobox 1, isoform p110; ***CXCL8***, C-X-C motif chemokine ligand 8; ***DCM***, dilated cardiomyopathy; ***DDX6***, DEAD-box helicase 6; ***DGCR8***, DGCR8 microprocessor complex subunit; ***DHCR24***, 24-dehydrocholesterol reductase; ***DKK2***, dickkopf WNT signaling pathway inhibitor 2; ***DSP***, desmoplakin; ***DTX1***, deltex E3 ubiquitin ligase 1; ***DTX3L***, deltex E3 ubiquitin ligase 3L; ***EGFR***, epidermal growth factor receptor; ***EIF3A***, eukaryotic translation initiation factor 3 subunit A; ***eNOS***, endothelial nitric oxide synthase; ***ER***, endoplasmic reticulum; ***ET***, electron transport; ***EV***, extracellular vesicle; ***FAK***, focal adhesion kinase; ***FBXO32***, F-box protein 32; ***FOXO1/-3***, forkhead box O1/-3; ***FTO***, FTO alpha-ketoglutarate dependent dioxygenase; ***GLUT4***, insulin-responsive glucose transporter type 4; ***GPX4***, glutathione peroxidase 4; ***GSK3β***, glycogen synthase kinase 3 β; ***HFpEF***, heart failure with preserved ejection fraction; ***HFrEF***, heart failure with reduced ejection fraction; ***H3K4me3***, tri-methylation at the 4th lysine residue of the histone H3 protein; ***HNRNPA2B1***, heterogeneous nuclear ribonucleoprotein A2/B1; ***H/R***, hypoxia-reoxygenation (injury); ***HuR***, human antigen R; ***ICAM-1***, intercellular adhesion molecule 1; ***IGF2BP1/-2/-3***, insulin-like growth factor 2 mRNA binding protein 1/2/3; ***INF-γ***, interferon γ; ***IL-6***, interleukin 6; ***I/R***, ischemia-reperfusion (injury); ***JAK2***, Janus kinase 2; ***KDM5A***, lysine demethylase 5A; ***KIAA1429***, alias ***VIRMA***, vir-like m^{6}A methyltransferase associated; ***KLF4/-5***, krüppel-like factor 4/-5; ***LATS1/-2***, Large tumor suppressor kinase 1/-2; ***L-PGDS***, prostaglandin D synthase; ***LPS***, lipopolysaccharide; ***MAGED1***, MAGE family member D1; ***MAVS***, mitochondrial antiviral-signaling protein; ***MDA5***, melanoma differentiation-associated protein 5; ***METTL3***, methyltransferase 3, N^6-adenosine-methyltransferase complex catalytic subunit; ***METTL14***, methyltransferase 14, N^6-adenosine-methyltransferase subunit; ***METTL16***, methyltransferase 16, N^6-methyladenosine ***Mhrt***, myosin heavy chain associated RNA transcript (a long non-coding RNA); ***MIAT***, myocardial infarction associated transcript (a long non-coding RNA); ***MST1***, macrophage stimulating 1; ***MYH7/-9***, myosin heavy chain 7/-9; ***NACAD***, NAC alpha domain containing; ***NEAT1***, nuclear paraspeckle assembly transcript 1 (a long non-coding RNA); ***NFATC4***, nuclear factor of activated T cells 4; ***NF-κB***, nuclear factor kappa B; ***NLRP1/-3***, NLR family pyrin domain containing 1/-3; ***NPPA***, natriuretic peptide A; ***PARP***, Poly-(ADP-ribose) polymerase; ***PM***, pulmonary hypertension; ***PMD***, pulmonary microvascular dysfunction; ***RYR2***, ryanodine receptor 2; ***SERCA2A***, sarcoplasmic/endoplasmic reticulum Ca^{2+}ATPase 2a; ***SLC7A5***, solute carrier family 7 member 5; ***SLC16A3***, solute carrier family 16 member 3; ***STAT3***, signal transducer and activator of transcription 3; ***PARP10***, poly(ADP-ribose) polymerase family member 10; ***PCNA***, proliferating cell nuclear antigen; ***PFKFB2***, 6-phosphofructo-2-kinase/fructose-2,6-biphosphatase 2; ***PGAM2***, phosphoglycerate mutase 2; ***PGD_2***, prostaglandin D2; ***PIWIL4***, piwi-like RNA-mediated gene silencing 4; ***PM2.5***, fine particulate matter, diameter <2.5 μm; ***PRKCE,*** protein kinase C epsilon type; ***p300***, E1A binding protein p300; ***SIRT1***, sirtuin 1; ***SPHK1***, sphingosine kinase 1; ***SULF2***, sulfatase 2; ***TanIIA***, Tanshinone IIA, ***TCA***, tricarboxylic acid (cycle); ***TGF-β1***, transforming growth factor β1; ***TFEB***, transcription factor EB; ***TINCR***, terminal differentiation-induced non-coding RNA (a long non-coding RNA); ***TNF-α,*** Tumor necrosis factor α; ***TSP1***, thrombospondin-1; ***TWIST1***, twist family bHLH transcription factor 1; ***VCAM-1***, vascular cell adhesion molecule 1; ***WNT1***, wnt family member 1; ***WNT5A***, Wnt family member 5A; ***WTAP***, WT1 associated protein; ***YAP1***, Yes1 associated transcriptional regulator; ***YTHDC1***, YTH domain containing 1; ***YTHDF1/-2***, YTH N^6-methyladenosine RNA binding protein 1/-2.

Table S4 Select summary of identified molecular pathways regarding m^{6}A and A-to-I RNA modifications and their key regulators in CVDs

Class	Process		Pathway / molecular associations: Upstream m^{6}A or A-to-I regulators		Inter-stream (e.g. target RNA and reader complex)		Downstream RNAs / proteins	Reference
CARDIAC	Cardiogenesis	↑	ADAR1p150			⊣	MDA5 → MVAS → INF → ER stress	[130,172-174]
	Regeneration, CM proliferation	↑	ALKBH5 → *YTHDF1* → YTHDF1	→	(m^{6}A-*YAP1* / YTHDF1)	→	YAP1	Han et al.[54]
			FTO	⊣	(m^{6}A-*Cdc42* / miR-133a / IGF2BP2 / AGO2)	⊣	CDC42	Qian et al.[124]
			METTL3	→	(pri-miR-17-3p / DGCR8)	→	miR-17-3p	Zhao et al.[147]
			ADAR2	→	edited-pri-miR-43a ⊣ miR-43a	⊣	SIRT1, Cyclin D1, BCL2	Wu et al.[146]
		↓	METTL3	→	(m^{6}A-pri-miR-143 / DGCR8) → miR-143-3p ⊣ *Yap1* / *Ctnnd1*	→	YAP1 / CTNND1	Gong et al.[140]
		↔	METTL3	→	*Dhcr24, Nacad, Slc16a3, Slc7a5*			Yang et al.[55]
				⊣	*Ank2, Cmya5, Fbxo32, Pfkfb2*			
	Cardiogenesis	↔	ADAR2	→	let-7 miRNA family / miR-29b			Altaf et al.[132]
	Hypertrophy	↑	(CHAPIR / PIWIL4) ⊣ METTL3	→	(m^{6}A-*Parp10* / YTHDF2)	→	PARP10 ⊣ GSK3β ⊣ NFATC4	Gao et al.[123]
			MIAT → *Ythdf2* → YTHDF2	→	(m^{6}A-*Cpt-1a* / YTHDF2)	⊣	CPT-1A	Yang et al.[129]
			USP12 → p300	→	*Mettl3*	→	METTL3	Lu et al.[126]
			Leptin	→	p-JAK2 → p-STAT3 → CTSL → CUX1 p110	→	FTO	Gan et al.[118]
		↓	FTO	→	(miR-133a / IGF2BP2 / AGO2)			Qian et al.[124]
			Maslinic acid	⊣	*Mettl3*	→	METTL3	Fang et al.[128]
			Tanshinone IIA ⊣ *Alkbh5* → ALKBH5	⊣	m^{6}A-*Galectin-3*	⊣	Galectin-3	Zhang et al.[141]
	Myocardial infarction	↑	KDM5A → H3K4me3 → METTL3	→	(m^{6}A-pri-miR-503/ HNRNPA2B1) → EV-packed miR-503	⊣	PGC-1β , SIRT1 → ET chain, TCA cycle	Sun et al.[148]
		↓	FTO	⊣	m^{6}A-(*Nppa, Myh7, Serca2a, Myh9, Ryr2, Ttn, Chast, Mhrt*)	→	SERCA2A	Mathiyalagan et al.[107]
	I/R, H/R injury	↑	WTAP	→	m^{6}A-*ATF4*	→	ATF4 → WTAP	Wang et al.[136]
			METTL3	→	(m^{6}A-*Tfeb* / HNRNPD)	→	TFEB → *Alkbh5* → ALKBH5; ⊣ *Mettl3* → METTL3	Song et al.[134]
			METTL3	→	(m^{6}A-pri-miR-143-3p / DGCR8) → miR-143-3p	⊣	PRKCE	Wang et al.[142]
		↓	METTL14	→	m^{6}A-*Wnt1*	→	WNT1 → β-catenin	Pang et al.[143]
			FTO	⊣	m^{6}A-*Mhrt* (lncRNA)	⊣	*Mhrt* (lncRNA)	Shen et al.[135]
			FTO	⊣	m^{6}A-*Yap1*	⊣	YAP1	Ke et al.[145]
	Cardiac fibrosis	↑	AngII → circCELF1	→	FTO ⊣ (m^{6}A-*Dkk2* / miR-363)	⊣	DKK2	Li et al.[149]
	HFpEF	(↓)	FTO	⊣	m^{6}A-*Pgam2*	⊣	PGAM2	Zhang et al.[175]
				→	p-AKT → *Glut4*	→	GLUT4	
	HFrEF	↔	FTO	⊣	m^{6}A-*Mhrt* (lncRNA)	⊣	*Mhrt* (lncRNA)	Shen et al.[135]
	DCM	↓			(m^{6}A-*Titin* / YTHDC1) → (alternative splicing) → *N2B-Titin*	→	N2B-Titin	Gao et al.[150]
	Diabetic cardiomyopathy	↑	ALKBH5	⊣	(m^{6}A-*Foxo3* / YTHDF2) ⊣ FOXO3	⊣	*CDR1as* (lncRNA) → MST1 → LATS1/2 ⊣ YAP1	Shao et al.[153]
		↓	METTL14	→	(m^{6}A-*TINCR* / YTHDF2) ⊣ *TINCR* (lncRNA)	→	*Nlrp3* → NLRP3	Meng et al.[152]
	AVC	↑	METTL3	→	(m^{6}A-*TWIST1* / YTHDF2)	⊣	TWIST1	Zhou et al.[157]
	CM inflammation	↑	Palmitic acid	→	CD36 [⊢ (*Cd36*-m^{6}A) ⊢ FTO]	→	TNF-α, IL-6, p-P65	Yu et al.[154]

Cont'd

Cont'd Table S4

Class	Process		Pathway / molecular associations: Upstream m^6A or A-to-I regulators		Inter-stream (e.g. target RNA and reader complex)		Downstream RNAs / proteins	Reference
**VASCULAR**	**Atherosclerosis, endotheliopathy**	↑	TNFα → METTL14	→	(m^6A-*FOXO1* / YTHDF1)	→	FOXO1 → VCAM-1, ICAM-1	Jian et al.[90]
			Oscillatory shear → METTL3*	→	(m^6A-*NLRP* / YTHDF1)	→	NLRP, (+ NF-κB)	Chien et al.[92]
				→	(m^6A-*KLF4* / YTHDF2)	⊣	KLF4	
			oxLDL → METTL3	→	(m^6A-*JAK2* / IGF2BP1)	→	JAK2 → p-STAT3	Dong et al.[97]
			METTL14	→	(m^6A-*pri-miR-19a* +DGCR8)	→	miR-19a	Zhang et al.[91]
			TNF-α → ADAR1	→	(edited-*NEAT1* (lncRNA) / AUF1)	→	*CCL2, CXL8, ICAM-1, VCAM-1*	Vlachogiannis et al.[103]
		↓	Oscillatory shear ⊣ METTL3*	→	m^6A-*EGFR*	⊣	EGFR	Li et al.[95]
		↔	TNF-α / INF-γ / hypoxia → ADAR1	→	(edited-*CTSS* / HuR)	→	CTSS	Stellos et al.[50]
	**Hypertension**	↑	Obesity → FTO	⊣	*L-Pdgs*	→	L-PDGS → PGD_2 → myogenic tone	Krüger et al.[117]
	**PH**	↑	WTAP	→	m^6A-*Gpx4*	→	GPX4	Wei et al.[79]
			METTL3	→	(m^6A-*MAGED / YTHDF1*)	→	MAGED1 → PCNA	Hu et al.[80]
	**PMD**	↑	PM2.5 → METTL16	→	m^6A-*Sulf2*	→	SULF2	Guo et al.[81]
	**Angiogenesis**	↑	ADAR1	→	(edited-CTSS / HuR)	→	CTSS	Stellos et al.[50]
			METTL3	→	mature let-7e-5p / miR-17-92 clusters ⊣ *TSP1*	⊣	TSP1	Chamorro-Jorganes et al.[56]
			METTL3	→	(m^6A-*DTXL3* / IGF2BP1,-3)	→	DTXL3 → DTX1/DTXL3 heterodimer	Wang et al.[106]
			WTAP, METTL3	→	(m^6A-*DSP* / IGF2BP1-3)	→	DSP	Wang et al.[105]
			WTAP	⊣	Wnt signaling (WT1-TBL1)	⊣	β-catenin	Wang et al.[105]
			LPS + hypoxia → ALKBH5	⊣	m^6A-*SPHK1*	⊣	SPHK1→ p-AKT → p-eNOS	Kumari et al.[109]
			FTO	⊣	(m^6A-*FAK / YTHDF2*)	⊣	FAK	Shan et al.[58]
		↓	Hypoxia → ALKBH5	⊣	m^6A-*Wnt5a*	→	WNT5A	Zhao et al.[104]
	**Aortic aneurysm**	↑	METTL3	→	(m^6A-pri-miR-34a / DGCR8)	→	miR-34a ⊣ SIRT1	Zhong et al.[86]
			ALKBH5	⊣	(m^6A-pri-miR-143-3p / DGCR8)	→	miR-143-3p ⊣ DDX6	Wang et al.[88]
			FTO	⊣	m^6A-*Klf5*	→	KLF5	Ma et al.[87]
				→	GSK3β phosphorylation (inactivation)	⊣	KLF5	
		↓	KIAA1429	→	(m^6A-pri-miR-143-3p / DGCR8)	→	miR-143-3p ⊣ DDX6	Wang et al.[88]

PREPRINT

TABLE S5. Selected summary of identified small molecules targeting m^{6}A regulators.

Abbreviations: ***A375***, human amelanotic melanoma cell line (RRID:CVCL_0132); A549, human lung adenocarcinoma cell line (RRID:CVCL_0023); ***ALKBH5***, alkB homolog 5, RNA demethylase; ***AML***, acute myeloid leukemia; ***ASB2***, ankyrin repeat and SOCS box containing 2; ***CEBPA***, CCAAT/enhancer-binding protein alpha; ***CCRF-CEM***, childhood T cell acute lymphoblastic leukemia cell line (RRID:CVCL_0207); ***FTO***, FTO alpha-ketoglutarate dependent dioxygenase; ***G0***, resting phase (cell cycle), ***G1***, period from mitosis to replication (cell cycle); ***HeLa***, human papillomavirus-related endocervical adenocarcinoma cell line (RRID:CVCL_0030); ***HL-60***, human adult acute myeloid leukemia cell line (RRID:CVCL_0002); ***K-562***, adult chronic myeloid leukemia cell line (RRID:CVCL_0004); ***KNS81***, human glioblastoma cell line (RRID:CVCL_2799); ***LN229***, human glioblastoma cell line (RRID:CVCL_0393); ***LSCs***, leukemia stem cells; ***MDA-MB-231***, human breast adenocarcinoma cell line (RRID:CVCL_0062); ***METTL3***, methyltransferase 3, N^6-adenosine-methyltransferase complex catalytic subunit; ***METTL14***, methyltransferase 14, N^6-adenosine-methyltransferase subunit; ***MIA PaCa-2***, adult human pancreatic cancer cell line (RRID:CVCL_0428); ***MOLM-13***, adult acute human myeloid cell line (RRID:CVCL_2119); ***MONOMAC6***, adult human acute monocytic leukemia cell line (RRID:CVCL_1426); ***MYC***, MYC proto-oncogene; ***NB4***, childhood human acute promyelocytic leukemia cell line (RRID:CVCL_0005); ***NOMO-1***, adult human acute monocytic leukemia cell line (RRID:CVCL_1609); ***PDX-AML***, patient-derived xenograft acute myeloid leukemia; ***RARA***, retinoic acid receptor alpha; ***SOCS1***, suppressor of cytokine signaling 1.

Table S5. A selection of identified compounds targeting enzymes regulating m^{6}A

Compound	Specific target	Disease / Model	Results	Reference
- STM2457	METTL3-14 inhibitor	Acute myeloid leukemia	Demethylation of leukaemogenic mRNAs inducing translational defect. Prolonged survival of various mice models of AML.	Yankova et al.[1]
- Eltrombopag	METTL3-14 inhibitor	AML cell line (MOLM-13)	Inhibited proliferation	Lee et al.[2]
- Quercetin	METTL3 inhibitor	MIA PaCa-2 Pancreatric cancer cell line	Dose-dependently decreased of m6A and inhibited proliferation	Du et al.[3]
- Methyl Piperidine-3-carboxylate Hydrochloride<br>- Tert-butyl 6-methylpiperidine-3-carboxylate<br>- Methyl 6-methylpiperidine-3-carboxylate<br>- Methyl Piperazine-2-carboxylate	METTL3-14-WTAP activators	HEK293 cells	Increased (1pM, **1-4**; 1µM; **2,4**) and decreased (10µM, **1-4**) RNA m^{6}A content, increase of G0/G1 cells (**1-3**), increased proliferation (**3-4**), delayed increase in DNA synthesis (**4**)	Selberg et al.[4]
- 4-amino-8-chloroquinoline-3-carboxylic acid<br>- 8-aminoquinoline-3-carboxylic acid	FTO inhibitors	Midbrain mouse dopaminergic neurons	Enhanced survival during growth factor deprivation, good penetrance through blood-brain barrier	Selberg et al.[5]
- Meclofenamic acid (MA)	FTO inhibitor	HeLa cells	Selective FTO inihibition and increased m^{6}A content	Huang et al.[6]
- Fluorescein derivatives	FTO inhibitor			Wang et al.[7]
- FB23 (designed MA)<br>- Benzohydroxamic acid (FB23-2)	FTO inhibitors	NB4 + MONOMAC6 cells<br><br>Mice (xeno-MONOMAC6)<br>Mice (PDX AML)	Weak effect on proliferation (low cellular uptake)<br>Anti-proliferative, prodifferentiative, proapototic, m6A increase<br>Suppressed leukemia progression and enhanced survival<br>Prolonged latency and survival, elimination of LSCs	Huang et al.[8]
- Rhein<br>- FB23-2	FTO inhibitors	Human obesity and hypertensive arteries	Increased myogenic tonus	Kruger et al.[9]
- 18077<br>- 18097	FTO inhibitors	Cancer cell lines (HeLa, MDA-MB-231, A549, A375)<br>Mice (xeno-MDA-MB-231)	Increase m6A and FTO stability, chemosensitivity, suppress proliferation, lipogenesis and invasive properties involving SOCS1<br>Smaller tumor volumes, reduced pulmonary colonization	Xie et al.[10]
- CS1 (Hydrophobic with micelles / β-cyclodextrin)<br>- CS2	FTO inhibitors	AML cell lines (multiple)<br>Mice (Leukemia stem cells)<br>NOMO-1 cells<br>Mice (PDX AML)	Reduced viability, increased apoptosis, differentiation, m6A level<br>Decreased levels, inhibited repopulation capacity<br>Decreased *MYC* + *CEBPA*, increased *RARA* + *ASB2* translation<br>Prolonged survival, reduced infiltratiton (above FB23-2)	Su et al.[11]
- FTO-4	FTO inhibitor	Patient-derived glioblastoma stem cells	Prevented neurosphere formation while no effect on neurosphere formation of healthy stem cells	Huff et al.[12]
- 2-[(1-hydroxy-2-oxo-2-phenylethyl)sulfanyl]acetic acid<br>- 4-{[(furan-2-yl)methyl]amino}-1,2-diazinane-3,6-dione	ALKBH5 inhibitors	Leukemia cells (HL-60, CCRF-CEM, K562)	Inhibited cell proliferation	Selberg et al.[13]
- Ena15<br>- Ena21	ALKBH5 inhibitors	Human glioblastoma multiforme cell lines KNS81 and LN229	Inhibited growth activity	Takahashi et al.[14]

A review discussing both FTO and ALKBH5 inhibitors identified by 22.5.2022: You et al.[15]

Supplemental references

1. Yankova, E., Blackaby, W., Albertella, M., Rak, J., De Braekeleer, E., Tsagkogeorga, G., Pilka, E.S., Aspris, D., Leggate, D., Hendrick, A.G., et al. (2021). Small-molecule inhibition of METTL3 as a strategy against myeloid leukaemia. Nature 593, 597-601. 10.1038/s41586-021-03536-w.
2. Lee, J.H., Choi, N., Kim, S., Jin, M.S., Shen, H., and Kim, Y.C. (2022). Eltrombopag as an Allosteric Inhibitor of the METTL3-14 Complex Affecting the m(6)A Methylation of RNA in Acute Myeloid Leukemia Cells. Pharmaceuticals (Basel) 15. 10.3390/ph15040440.
3. Du, Y., Yuan, Y., Xu, L., Zhao, F., Wang, W., Xu, Y., and Tian, X. (2022). Discovery of METTL3 Small Molecule Inhibitors by Virtual Screening of Natural Products. Front Pharmacol 13, 878135. 10.3389/fphar.2022.878135.
4. Selberg, S., Blokhina, D., Aatonen, M., Koivisto, P., Siltanen, A., Mervaala, E., Kankuri, E., and Karelson, M. (2019). Discovery of Small Molecules that Activate RNA Methylation through Cooperative Binding to the METTL3-14-WTAP Complex Active Site. Cell Rep 26, 3762-3771 e3765. 10.1016/j.celrep.2019.02.100.
5. Selberg, S., Yu, L.Y., Bondarenko, O., Kankuri, E., Seli, N., Kovaleva, V., Herodes, K., Saarma, M., and Karelson, M. (2021). Small-Molecule Inhibitors of the RNA M6A Demethylases FTO Potently Support the Survival of Dopamine Neurons. Int J Mol Sci 22. 10.3390/ijms22094537.
6. Huang, Y., Yan, J., Li, Q., Li, J., Gong, S., Zhou, H., Gan, J., Jiang, H., Jia, G.F., Luo, C., et al. (2015). Meclofenamic acid selectively inhibits FTO demethylation of m6A over ALKBH5. Nucleic Acids Res 43, 373-384. 10.1093/nar/gku1276.
7. Wang, T., Hong, T., Huang, Y., Su, H., Wu, F., Chen, Y., Wei, L., Huang, W., Hua, X., Xia, Y., et al. (2015). Fluorescein Derivatives as Bifunctional Molecules for the Simultaneous Inhibiting and Labeling of FTO Protein. J Am Chem Soc 137, 13736-13739. 10.1021/jacs.5b06690.
8. Huang, Y., Su, R., Sheng, Y., Dong, L., Dong, Z., Xu, H., Ni, T., Zhang, Z.S., Zhang, T., Li, C., et al. (2019). Small-Molecule Targeting of Oncogenic FTO Demethylase in Acute Myeloid Leukemia. Cancer Cell 35, 677-691 e610. 10.1016/j.ccell.2019.03.006.
9. Kruger, N., Biwer, L.A., Good, M.E., Ruddiman, C.A., Wolpe, A.G., DeLalio, L.J., Murphy, S., Macal, E.H., Jr., Ragolia, L., Serbulea, V., et al. (2020). Loss of Endothelial FTO Antagonizes Obesity-Induced Metabolic and Vascular Dysfunction. Circ Res 126, 232-242. 10.1161/CIRCRESAHA.119.315531.
10. Xie, G., Wu, X.N., Ling, Y., Rui, Y., Wu, D., Zhou, J., Li, J., Lin, S., Peng, Q., Li, Z., et al. (2022). A novel inhibitor of N (6)-methyladenosine demethylase FTO induces mRNA methylation and shows anti-cancer activities. Acta Pharm Sin B 12, 853-866. 10.1016/j.apsb.2021.08.028.
11. Su, R., Dong, L., Li, Y., Gao, M., Han, L., Wunderlich, M., Deng, X., Li, H., Huang, Y., Gao, L., et al. (2020). Targeting FTO Suppresses Cancer Stem Cell Maintenance and Immune Evasion. Cancer Cell 38, 79-96 e11. 10.1016/j.ccell.2020.04.017.
12. Huff, S., Tiwari, S.K., Gonzalez, G.M., Wang, Y., and Rana, T.M. (2021). m(6)A-RNA Demethylase FTO Inhibitors Impair Self-Renewal in Glioblastoma Stem Cells. ACS Chem Biol 16, 324-333. 10.1021/acschembio.0c00841.
13. Selberg, S., Seli, N., Kankuri, E., and Karelson, M. (2021). Rational Design of Novel Anticancer Small-Molecule RNA m6A Demethylase ALKBH5 Inhibitors. ACS Omega 6, 13310-13320. 10.1021/acsomega.1c01289.
14. Takahashi, H., Hase, H., Yoshida, T., Tashiro, J., Hirade, Y., Kitae, K., and Tsujikawa, K. (2022). Discovery of two novel ALKBH5 selective inhibitors that exhibit uncompetitive or competitive type and suppress the growth activity of glioblastoma multiforme. Chem Biol Drug Des. 10.1111/cbdd.14051.
15. You, Y., Fu, Y., Huang, M., Shen, D., Zhao, B., Liu, H., Zheng, Y., and Huang, L. (2022). Recent Advances of m6A Demethylases Inhibitors and Their Biological Functions in Human Diseases. Int J Mol Sci. 23, 5815. 10.3390/ijms2310581
